# Design, Synthesis, and Antitumor Activity of a Series of Novel 4-(Aromatic Sulfonyl)-1-oxa-4-azaspiro[4.5]deca-6,9-dien-8-ones

**DOI:** 10.3390/molecules25225459

**Published:** 2020-11-21

**Authors:** Naiguo Xing, Chen Chen, Qiu Zhong, Shilong Zheng, Guangdi Wang, Ling He

**Affiliations:** 1 Key Laboratory of Drug-Targeting and Drug Delivery System of the Education Ministry, Department of Medicinal Chemistry, West China School of Pharmacy, Sichuan University, Chengdu 610041, Sichuan, China; xingng1982@163.com (N.X.); cc_45@live.cn (C.C.); 2 Sichuan Engineering Laboratory for Plant–Sourced Drug and Sichuan Research Center for Drug Precision Industrial Technology, Department of Medicinal Chemistry, West China School of Pharmacy, Sichuan University, Chengdu 610041, Sichuan, China; 3 RCMI Cancer Research Center and Department of Chemistry, Xavier University of Louisiana, New Orleans, LA 70125, USA; qzhong@xula.edu (Q.Z.); szheng@xula.edu (S.Z.)

**Keywords:** sulfonylazaspirodienone, metal-catalyzed cascade cyclization, antitumor activity

## Abstract

Many sulfonamides show anticancer activity. Based on benzenesulfonylazaspirodienone (HL-X9) identified in our previous work, we optimized the lead compound for better efficacy, thereby synthesizing a series of novel 4-(aromatic sulfonyl)-1-oxa-4-azaspiro[4.5]deca-6,9-dien-8-one derivatives through a key step of metal-catalyzed cascade cyclization. The preliminary antiproliferative tests have shown that the anticancer activities of acetyl-protected mannose-linked sulfonylazaspirodienone derivatives (**7i**–**7l**) have been greatly improved. Among them, **7j** is the most potent derivative, with IC_50_ values of 0.17 µM, 0.05 µM, and 0.07 µM for A549, MDA-MB-231, and HeLa cell lines, respectively. Flow cytometry analysis shows that **7j** arrests MDA-MB-231 cells in the G2/M phase and has a certain effect on the apoptosis of MDA-MB-231 cells. In addition, the acute toxicity of **7j** was lower than that of adriamycin.

## 1. Introduction

**Figure 1:**
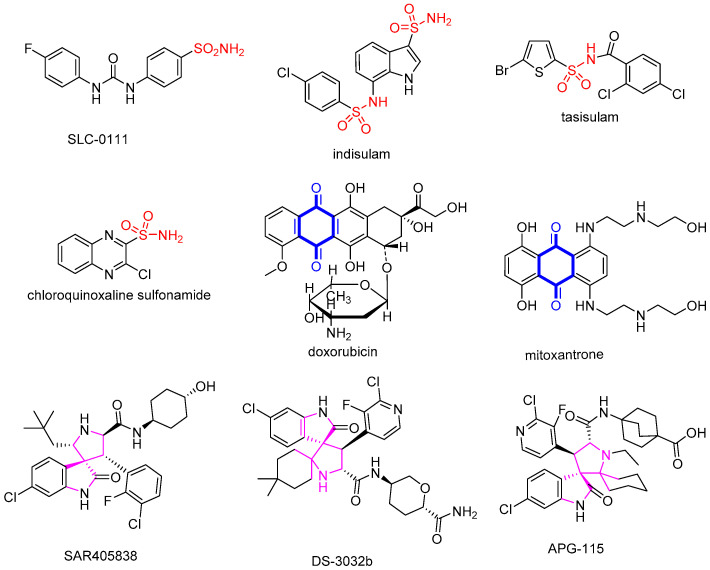
Sulfonamide, quinone, and spirocyclic antitumor agents.

Sulfonamide is a functional moiety of several types of drugs with a variety of biological activities [1,2]. Although there are no clinically approved sulfonamide anticancer drugs, many reports have shown that many sulfonamides have anticancer activity [3,4,5]. Recently, carbonic anhydrase (CA) isozymes II, IX, and XII have been shown to be involved in tumor formation, sulfonamide-based CA IX and CA XII inhibitor have become a new research topic of anticancer drug development [6,7,8,9]. Many sulfonamides have been identified as low nanomolar CA IX inhibitors, SLC-0111 of which has entered the clinical trial (NCT02215850) (Figure 1). The research also found that CUL4-DCAF15 E3 ubiquitin ligase can be recruited by sulfonamide compounds such as indisulam, tasisulam, and chloroquinoxaline sulfonamide (CQS) (Figure 1) to trigger ubiquitination and degradation of RBM39 protein, resulting in an antitumor effect [10]. Besides, sulfonamides can be selectively concentrated in tumor tissues [11,12,13] and are expected to be useful for the development of tumor-targeting drugs.

Quinone is also a key moiety of many biologically active molecules [14]. Among them, doxorubicin and mitoxantrone (Figure 1) have been the first-line anticancer drugs for solid tumors for many years, and the research on quinone-based anticancer drugs is still ongoing [15,16]. In addition, the inclusion of a spiro ring structure is one of the important strategies for drug development in recent years [17,18]. Some antitumor compounds with a spiro ring moiety such as SAR405838, DS-3032b, and APG-115 are undergoing clinical trials (Figure 1) [19,20,21]. Spirodienone derivatives of hybridized quinone and spiro scaffolds have a wide range of biological activities [22].

**Figure 2:**
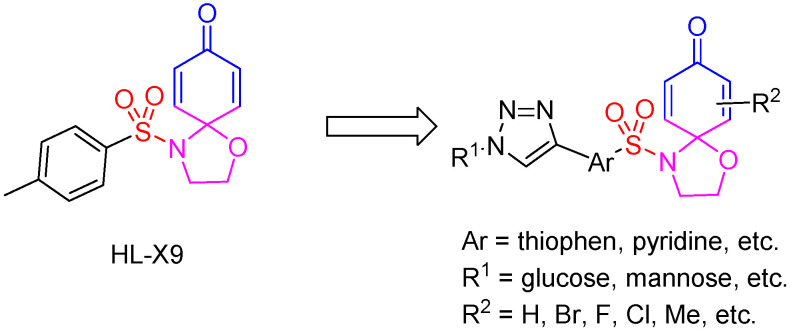
Design of novel sulfonylazaspirodienone derivatives from 4-tosyl-1-oxa-4-azaspire[4.5]deca-6,9-dien-8-one (HL-X9).

Previously, we have hybridized these pharmacologically active structural elements (sulfonamide, quinone, and spirocyclic moieties) and obtained a series of benzenesulfonylazaspirodienones. [23]. The preliminary screening proved that these compounds have a certain inhibitory effect on cancer cell lines [24]. However, the aqueous solubility is low, and the in vitro activity is mostly at the μM level. At the same time, because the *α,β*-dienone structure in the spiro compound is a Michael acceptor, it is easy to have Michael’s addition reaction with active nucleophilic molecules in vivo, resulting in toxic side effects. To improve the efficacy, the water solubility of the compounds, and improve the Michael acceptor characteristics of α,β-dienone structure in the spirodienone, reduce the possibility of addition reaction with active molecules in vivo to reduce toxicity and side effects. Therefore we further optimize the sulfonamide azaspirodienone by introducing the water-soluble 1,2,3-triazole moiety [25,26] through “Click Reaction” [27,28], replacing the benzene ring with a more polar heterocycle, and introducing glucose [29] or mannose [30] to affect the inhibitory effect of tumor growth and tumor targeting and so on (Figure 2). Herein, we report the synthesis of these newly designed sulfonamide azaspirocyclodienone derivatives and their preliminary antiproliferative activity on cancer cell lines and flow cytometry analysis.

## 2. Results and Discussion

### 2.1. Chemical Synthesis

**Scheme 1:**
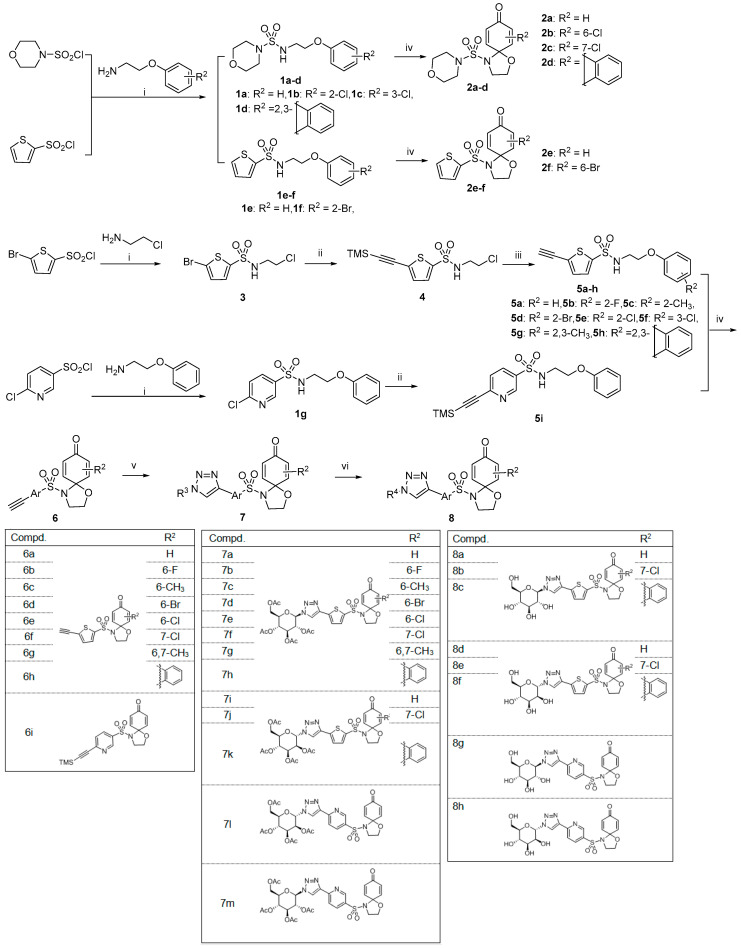
Synthesis route of sulfonylazaspirodienone derivatives. Reagents and conditions: (**i**) Et_3_N, CH_2_Cl_2_, r.t.; (**ii**)Trimethyl silyl acetylene, Pd(PPh_3_)_2_Cl_2_, CuI, Et_3_N, THF, r.t; (**iii**) Phenol derivatives, NaH, DMF, 50 °C, 2 h; (**iv**) PhI(CF_3_COO)_2_, Rh_2_(OAc)_4_, CH_2_Cl_2_, r.t., 5 h; (**v**) Sodium l-Ascorbate, CuSO_4_.5H_2_O, R_3_N_3_, DMSO, H_2_O, r.t., 3 h; (**vi**) K_2_CO_3_, MeOH, r.t., 30 min.

The sulfonylazaspirodienone derivatives were prepared according to the synthetic route of Scheme 1. Sulfonylation of chloroethylamine hydrochloride by bromo-substituted aromatic sulfonyl chloride afforded the sulfonamides **3,** which were converted to **4** by a Sonogashira coupling reaction. *O*-Alkylation of phenols with **4** formed the intermediates **5**. Rhodium acetate-catalyzed oxidation and concomitant intramolecular amidation of **5** resulted in the formation of **6** by bis(trifluoroacetoxy)iodobenzene under mild conditions [23]. The copper-catalyzed click reaction of **6** with the azides [31,32] produced the derivatives **7**. Further, **7** were hydrolyzed to the derivatives **8** with potassium carbonate in methanol.

The reaction of morpholine-4-sulfonyl chloride and thiophene-2-sulfonyl chloride with (substituted) phenoxyethylamine in dichloromethane gave **1a–f**. Compounds **2** were formed by rhodium acetate-catalyzed oxidative amidation of **1a**–**f** [23]. Intermediate **5i** for the synthesis of compounds **8g**–**h** was obtained from **1g** by a Sonogashira reaction. **1g** was the product of the reaction of 2-chloropyridine-5-sulfonyl chloride with 2-phenoxyethylamine.

### 2.2. Inhibition of Tumor Cell Proliferation In Vitro

To determine the cytotoxicity of the synthesized sulfonylazaspirodienones, we used three well-established cancer cell lines that are relevant to the study of in vitro anticancer properties. A549 is one of the most abundant human non-small cell lung cancer that has been widely used in screening anticancer agents. HeLa is a cervical cancer cell line commonly used in in vitro cancer research. MDA-MB-231 is a triple-negative breast cancer cell line that is widely used in cytotoxicity assays. The three cell lines have been shown to be viable cell lines for tumor xenografts in C57BL/6 nude mice and can be subsequently used to examine the in vivo effects of cytotoxicity on cancer.

**Scheme 2:**
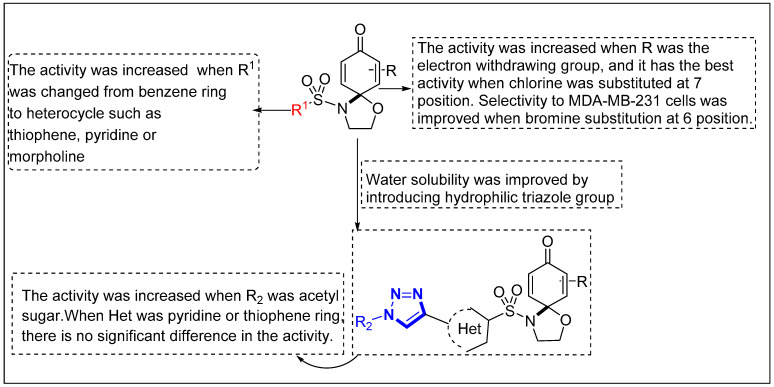
Structure-activity relationship of sulfonylazaspirodienone derivatives.

We used 4-tosyl-1-oxa-4-azaspire[4.5]deca-6,9-dien-8-one (HL-X9) as positive controls to investigate the inhibitory activity of the new sulfonylazaspirodienone derivatives on lung carcinoma A549, breast adenocarcinoma MDA-MB-231, and cervical cancer HeLa cells. The results (Table 1) showed that the replacement of benzene ring of HL-X9 with thiophene and morpholine heterocycle increased the inhibitory activity of **2a** and **2e** from HL-X9 by 2–4 times on MDA-MB-231 and HeLa cell lines. It was also found that the 7-chloro-substituted (on core structure) derivatives (**2c** and **7f**) were more potent than the 6-chloro-substituted derivatives (**2b** and **7e**) against A549, MDA-MB-231 and HeLa cell lines. The introduction of triazole-bridged acetyl-protected mannose further improved the antiproliferative potency of **7i** and **7l** from **2e**, especially in A549 cell line. Comparison of IC_50_ values of **7a**, **7f**, and **7h** with **7i**, 7**j**, **7k**, and **7l** indicates that the acetyl-protected mannose derivatives are better than the acetyl-protected glucose derivatives for anticancer activity. Undesirably, the activity of the acetyl-deprotected derivatives (**8a**-**h**) decreased significantly compared with the corresponding **7a**, **7f**, **7h**, **7i**, 7**j**, **7k**, and **7l**. The most potent derivative **7j** has IC_50_ values of 0.17 µM, 0.05 µM, and 0.07 µM against A549, MDA-MB-231, and HeLa cancer cell lines, respectively. **7j,** together with **2a**, **2c**, **2e**, **7f**, **7i**, and **7l** are all more effective than HL-X9 in inhibiting all three cancer cell lines. In addition, IC_50_ values of the derivatives **2c**, **7d**, **7h**, **7k,** and **7l**, together with **7f** and **7j**, reached a double-digit nanomolar concentration level for the MDA-MB-231 cell line. Derivative **7j** is the only compound with an IC_50_ value of a double-digit nanomolar concentration level against the HeLa cell line. The structure-activity relationship of sulfonylazaspirodienone derivatives is summarized in Scheme 2**.**

### 2.3. Cell Cycle Arresting

**Figure 3:**
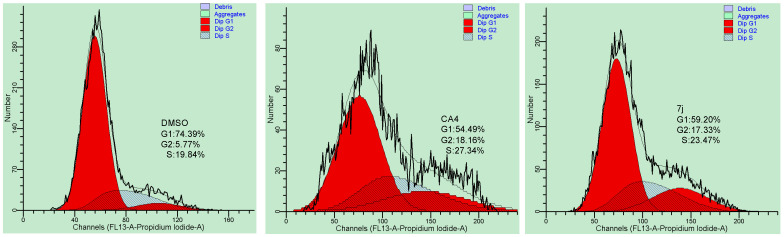
MDA-MB-231 cell cycle distribution of CA4 (1 μM) and **7j** (1 μM).

Next, we explored the effect of compound **7j** in the regulation of cell cycle distribution by flow cytometry. Combretastatin A4 (CA4) was used as a positive control. As shown in Figure 3, compound **7j** at the concentration of 1 μM was sufficient to arrest MDA-MB-231 cells in the G2/M phase, equivalent to CA4.

### 2.4. Apoptosis

**Figure 4:**
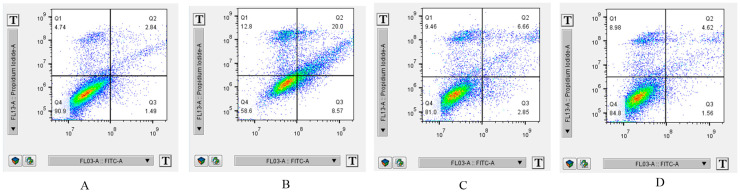
MDA-MB-231 cell apoptosis. **A**: DMSO; **B**: CA4 (1 μM); **C**: **7j** (1 μM); **D**: **7j** (0.1 μM).

To explore the potential mechanisms of the antiproliferative effect induced by sulfonylazaspirodienone derivatives, annexin V-FITC and PI staining were used, and flow cytometry (FCM) was performed to quantify cell apoptosis (Figure 4). MDA-MB-231 cells were cultured with compound **7j** at concentrations of 0.1 and 1 μM, respectively. Combretastatin A4 (CA4) was used as a positive control. Treatment with a low concentration (0.1 μM) of **7j** altered the number of apoptotic cells compared to DMSO. The apoptosis percentage increased after treatment with higher concentrations (1 μM) of compounds **7j**.

### 2.5. Acute Toxicity

**Figure 5:**
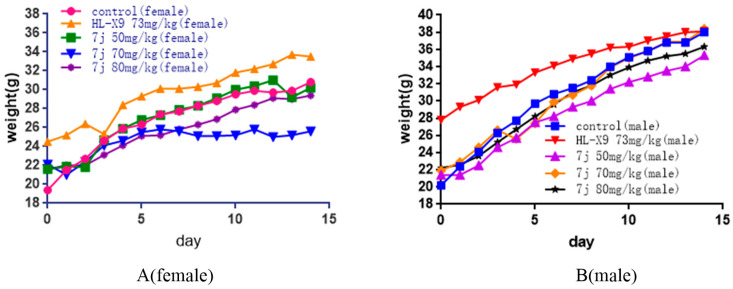
Weight change. **A**: Female; **B**: Male.

The acute toxicity of compound HL-X9 and **7j** were evaluated preliminarily in Kun Ming mice. All animals did not lose weight after drug treatment. Intraperitoneal injection of 30 mg/kg **7j** or 60 mg/kg HL-X9 did not cause death within 14 days. The mortality after administration of 80 mg/kg **7j** and 73 mg/kg HL-X9 was 40% and 10%, respectively, and the toxicity was lower than that of adriamycin (LD_50_ = 10.7 mg/kg i.p., Hazardous Substances Data Bank) [33]. The tolerance of female mice to compound **7j** was poor, and the mortality rate of female mice was 4 times higher than that of male mice. Different doses of compound HL-X9 and **7j** did not cause weight loss. Except that the weight gain of female mice was affected by compound **7j**, the weight gain of other groups was similar to that of the control group (Figure 5).

**Figure 6:**
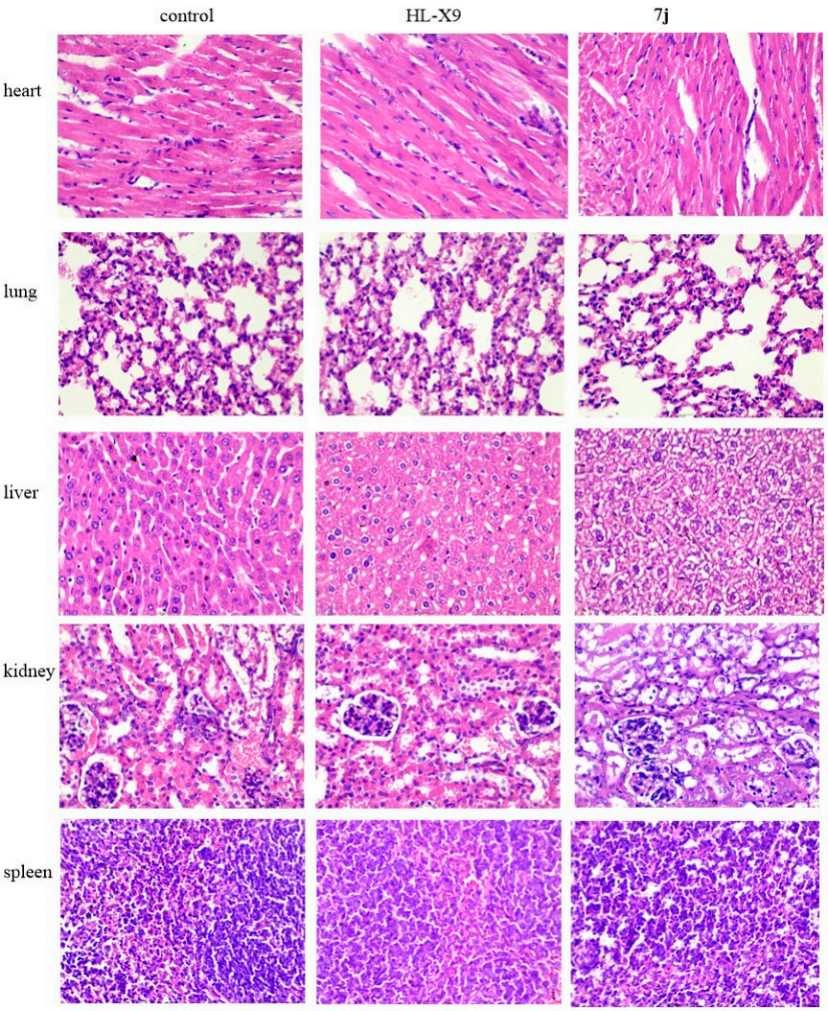
H&E staining of main tissues female mice after administration.

In addition, the main tissues (heart, liver, spleen, lung, and kidney) of mice in each group were stained with Hematoxylin and Eosin (H&E). The results showed that compared with the negative control group, no obvious damage was found in the sections of compound HL-X9 (73 mg/kg) in all tissues; the sections of compound **7j** (80 mg/kg) showed some damage in the liver and kidney, and no obvious damage was found in other tissues. In particular, the liver and kidney damage was more serious in female mice (Figure 6).

## 3. Experimental Section

### 3.1. General Chemistry

2,3,4,6-*O*-tetraacetoxy-1-azido-β-d-glucose and 2,3,4,6-tetraacetoxy-1-azido-α-d-mannose were synthesized according to literature methods [31,32]. Commercially available materials were purchased from Energy Chemical (Shanghai, China), Bidepharm (Shanghai, China), Titan (Shanghai, China), and were used as received without further purification. Solvents were purchased from Titan. Dichloromethane, THF, and triethyl amine were distilled after the treatment with P_2_O_5_, metal sodium, and calcium hydride, respectively. Reactions were monitored by thin-layer chromatography (TLC) carried out on commercial silica gel plates(Yantai Jiangyou Silica gel Development Co., LTD, Yantai, China) using UV light as a visualizing agent. Commercial silica gel (Qingdao Haiyang Chemical Co., LTD, Qingdao, China) was used for column chromatography. ^1^H- and ^13^C-NMR spectra were recorded on 400 MHz or 600 MHz spectrometers(Agilent Tech., Palo Alto, USA). ^1^H-NMR spectra were referenced to Chloroform-*d* (7.26 ppm) or DMSO-*d6* (2.50 ppm), and reported as follows: Chemical shift, multiplicity (s = singlet, d = doublet, t = triplet, q = quartet, m = multiplet). Chemical shifts of the ^13^C-NMR spectra were measured relative to chloroform-*d* (77.23 ppm) or DMSO-*d6* (39.51 ppm). Mass spectral data were obtained from Bruker Daltonics Data analysis 3.2 mass spectrometer(Bruker, Beijing, China). Unless specified otherwise, all tested compounds were confirmed to be >95% pure by HPLC. ^1^H-NMR and ^13^C-NMR spectra of compounds can be found in the Supplementary Material.

### 3.2. Chemical Synthesis

#### 3.2.1. Procedure for the Synthesis of Compound **1**

To a round-bottom flask were added 2-phenoxy ethylamine derivative (1.2 mmol) and sulfonyl chloride (1 mmol), then dichloromethane (3 mL) and triethylamine (1.5 mmol) were added at 0 °C. The mixture was stirred at room temperature and was monitored by TLC. Upon completion, the reaction solution was washed with saturated aqueous NaCl and dried with Na_2_SO_4_. The solvent was removed under vacuum, and the residue was purified by column chromatography on silica gel with hexane-ethyl acetate (3:1 to 2:1) as eluent to afford the desired product **1**.

*N-(2-Phenoxyethyl)morpholine-4-sulfonamide* (**1a**): 92% yield as a yellowish solid. ^1^H-NMR (400 MHz, Chloroform-*d*) δ 7.30 (m, 2H), 6.99 (t, *J* = 7.4 Hz, 1H), 6.92–6.86 (m, 2H), 4.67 (s, 1H), 4.10 (t, *J* = 5.0 Hz, 2H), 3.77–3.68 (m, 4H), 3.49 (q, *J* = 5.0 Hz, 2H), 3.25–3.18 (m, 4H). ^13^C-NMR (100 MHz, Chloroform-*d*) δ 158.1, 129.7, 121.5, 114.4, 66.4, 66.2, 46.2, 43.2.

*N-(2-(2-Chlorophenoxy)ethyl)morpholine-4-sulfonamide* (**1b**): 94% yield as a yellowish solid. ^1^H-NMR (400 MHz, Chloroform-*d*) δ 7.38 (dd, *J* = 7.9, 1.6 Hz, 1H), 7.26–7.20 (m, 1H), 6.98–6.90 (m, 2H), 4.77 (t, *J* = 5.4 Hz, 1H), 4.20–4.12 (m, 2H), 3.75–3.67 (m, 4H), 3.54 (q, *J* = 5.4 Hz, 2H), 3.26–3.19 (m, 4H). ^13^C-NMR (100 MHz, Chloroform-*d*) δ 153.6, 130.5, 127.9, 123.1, 122.4, 113.9, 67.8, 66.2, 46.2, 43.2.

*N-(2-(3-Chlorophenoxy)ethyl)morpholine-4-sulfonamide* (**1c**): 92% yield as a yellowish solid. ^1^H-NMR (400 MHz, Chloroform-*d*) δ 7.22 (t, *J* = 8.2 Hz, 1H), 7.00–6.95 (m, 1H), 6.89 (t, *J* = 2.2 Hz, 1H), 6.78 (dd, *J* = 8.2, 2.2 Hz, 1H), 4.65 (s, 1H), 4.09 (t, *J* = 5.0 Hz, 2H), 3.78–3.69 (m, 4H), 3.49 (q, *J* = 5.0 Hz, 2H), 3.26–3.19 (m, 4H). ^13^C-NMR (100 MHz, Chloroform-*d*) δ 158.8, 135.1, 130.5, 121.7, 114.9, 112.9, 66.8, 66.2, 46.2, 43.1.

*N-(2-(Naphthalen-1-yloxy)ethyl)morpholine-4-sulfonamide* (**1d**): 93% yield as a yellowish solid. ^1^H-NMR (400 MHz, Chloroform-*d*) δ 8.19 (d, *J* = 9.1 Hz, 1H), 7.82 (d, *J* = 9.1 Hz, 1H), 7.57–7.45 (m, 3H), 7.38 (t, *J* = 7.9 Hz, 1H), 6.82 (d, *J* = 7.9 Hz, 1H), 4.71 (t, *J* = 5.4 Hz, 1H), 4.31 (t, *J* = 5.4 Hz, 2H), 3.72 (m, 4H), 3.66 (q, *J* = 5.4 Hz, 2H), 3.24 (m, 4H). ^13^C-NMR (100 MHz, Chloroform-*d*) δ 153.8, 134.6, 127.7, 126.7, 125.7, 125.5, 125.3, 121.5, 121.2, 104.9, 66.8, 66.1, 46.2, 43.4.

*N-(2-Phenoxyethyl)thiophene-2-sulfonamide* (**1e**): 95% yield as a yellowish solid. ^1^H-NMR (400 MHz, Chloroform-*d*) δ 7.67–7.63 (m, 1H), 7.61–7.58 (m, 1H), 7.29 (t, *J* = 7.7 Hz, 2H), 7.12–7.07 (m, 1H), 6.97 (t, *J* = 7.4 Hz, 1H), 6.82 (d, *J* = 8.0 Hz, 2H), 5.04 (t, *J* = 5.3 Hz, 1H), 4.03 (t, *J* = 5.3 Hz, 2H), 3.46 (q, *J* = 5.3 Hz, 2H). ^13^C-NMR (100 MHz, Chloroform-*d*) δ 158.0, 140.8, 132.3, 132.1, 129.6, 127.5, 121.5, 114.5, 66.1, 42.9.

*N-(2-(2-Bromophenoxy)ethyl)thiophene-2-sulfonamide* (**1f**): 93% yield as a yellowish solid. ^1^H-NMR (400 MHz, Chloroform-*d*) δ 7.65 (dd, *J* = 3.7, 1.0 Hz, 1H), 7.58 (dd, *J* = 5.0, 1.0 Hz, 1H), 7.53 (dd, *J* = 7.9, 1.3 Hz, 1H), 7.25–7.21 (m, 1H), 7.11–7.04 (m, 1H), 6.91–6.85 (m, 1H), 6.84–6.80 (m, 1H), 5.17 (t, *J* = 5.4 Hz, 1H), 4.09 (t, *J* = 5.4 Hz, 2H), 3.50 (q, *J* = 5.4 Hz, 2H). ^13^C-NMR (100 MHz, Chloroform-*d*) δ 154.3, 140.8, 133.4, 132.3, 132.1, 128.7, 127.5, 122.9, 113.9, 112.5, 67.7, 42.9.

#### 3.2.2. General Procedure for the Synthesis of Compound **5a**–**h**

To a round-bottom flask were added 2-chloroethylamine hydrochloride (1.2 mmol) and 5-bromothiophene-2-sulfonyl chloride (261 mg, 1 mmol), then dichloromethane (3 mL) and triethylamine (2.5 mmol) were added at 0 °C. The mixture was stirred at room temperature and was monitored by TLC. Upon completion, the reaction solution was washed with 2 N HCl and saturated aqueous NaCl and dried with Na_2_SO4. The solvent was evaporated to give the desired compounds 5-bromo-*N*-(2-chloroethyl)thiophene-2-sulfonamide (**3**, 289 mg, 95% yield) as a yellowish solid. ^1^H-NMR (400 MHz, Chloroform-*d*) δ 7.32 (d, *J* = 4.0 Hz, 1H), 7.02 (d, *J* = 4.0 Hz, 1H), 5.11 (s, 1H), 3.56 (t, *J* = 5.7 Hz, 2H), 3.33 (q, *J* = 5.7 Hz, 2H).^13^C-NMR (150 MHz, Chloroform-*d*) δ 141.4, 132.5, 130.5, 120.3, 44.9, 43.3.

To an oven-dried reaction tube were added PdCl_2_(PPh_3_) (0.02 mmol), CuI (0.04 mmol) and compounds **3** (1 mmol) under N_2_. Then THF (2 mL), Et_3_N (1.5 mmol) and trimethylsilylacetylene (1.25 mmol) were added successively by syringe. The mixture was stirred at room temperature and was monitored by TLC. Upon completion, the reaction solution was condensed and purified by column chromatography on silica gel with hexane-ethyl acetate as eluent to afford the desired *N*-(2-chloroethyl)-5-((trimethylsilyl)ethynyl)thiophene-2-sulfonamide (**4**, 258 mg, 80% yield) as a yellowish solid. ^1^H-NMR (600 MHz, Chloroform-*d*) δ 7.47 (d, *J* = 3.9 Hz, 1H), 7.16 (d, *J* = 3.9 Hz, 1H), 5.06 (t, *J* = 5.9 Hz, 1H), 3.62 (t, *J* = 5.9 Hz, 2H), 3.41 (q, *J* = 5.9 Hz, 2H), 0.28 (s, 9H).^13^C-NMR (150 MHz, Chloroform-*d*) δ 140.6, 132.2, 131.8, 130.2, 103.6, 95.2, 44.9, 43.3, -0.4.

To an oven-dried round-bottom flask were added THF (10 mL) and phenol derivatives (2 mmol). The solution was cooled to 0 °C and NaH (5 mmol) was added in portions. The mixture was stirred for 2 h at room temperature, and then the solvent was removed under vacuum. DMF (5 mL), **4** (1 mmol), and Benzyltriethylammonium chloride(TEBA) (0.3 mmol) were added successively to the residue. The mixture was stirred at 50 °C and was monitored by TLC. Upon completion, water was added, and the reaction was extracted twice with CH_2_Cl_2_. The combined organic layer was washed with saturated aqueous NaCl and dried with Na_2_SO_4_. The solvent was removed under vacuum, and the residue was purified by column chromatography on silica gel with hexane-ethyl acetate (4:1) as eluent to afford compounds **5a**–**h**.

#### 3.2.3. Procedure for the Synthesis of Compound **5i**

To a round-bottom flask were added 2-phenoxy ethylamine(1.2 mmol) and 2-chloropyridine-5-sulfonyl chloride (1 mmol), then dichloromethane (3 mL) and triethylamine (1.5 mmol) were added at 0 °C. The mixture was stirred at room temperature and was monitored by TLC. Upon completion, the reaction solution was washed with saturated aqueous NaCl and dried with Na_2_SO4. The solvent was removed under vacuum, and the residue was purified by column chromatography on silica gel with hexane-ethyl acetate as eluent to afford the desired product **1g** (75% yield) as a white solid. ^1^H-NMR (400 MHz, Chloroform-*d*) δ 8.88 (d, *J* = 2.5 Hz, 1H), 8.08 (dd, *J* = 8.4, 2.5 Hz, 1H), 7.44 (d, *J* = 8.4 Hz, 1H), 7.29 (t, *J* = 7.7 Hz, 2H), 6.98 (t, *J* = 7.4 Hz, 1H), 6.79 (d, *J* = 8.0 Hz, 2H), 5.13 (t, *J* = 5.3 Hz, 1H), 4.03 (t, *J* = 5.3 Hz, 2H), 3.45 (q, *J* = 5.3 Hz, 2H). ^13^C-NMR (100 MHz, Chloroform-*d*) δ 157.8, 155.6, 148.3, 137.2, 135.8, 129.7, 124.8, 121.7, 114.3, 66.0, 42.7.

To an oven-dried reaction tube were added PdCl_2_(PPh_3_) (0.1 mmol), CuI (0.2 mmol), and compound **1g** (1 mmol) under N_2_. Then THF (2 mL), Et_3_N (1.5 mmol), and trimethylsilylacetylene (1.25 mmol) were added successively by syringe. The mixture was stirred at room temperature and was monitored by TLC. Upon completion, the reaction solution was condensed and purified by column chromatography on silica gel with hexane -ethyl acetate (2:1) as eluent to afford the desired **5i**.

*5-Ethynyl-N-(2-phenoxyethyl)thiophene-2-sulfonamide* (**5a**): 49% yield as a yellowish solid. ^1^H-NMR (400 MHz, Chloroform-d) δ7.47 (d, J = 3.9 Hz, 1H), 7.31–7.27 (m, 2H), 7.19 (d, J = 3.9 Hz, 1H), 7.01–6.97 (m, 1H), 6.84–6.82 (m, 2H), 5.11 (t, J = 6.1 Hz, 1H), 4.05 (t, J = 6.0 Hz, 2H), 3.50-3.45 (m, 3H). ^13^C-NMR (150 MHz, Chloroform-*d*) δ157.9, 141.5, 132.8, 131.5, 129.6, 128.7, 121.6, 114.4, 84.8, 75.1, 66.0, 42.9.

*5-Ethynyl-N-(2-(2-fluorophenoxy)ethyl)thiophene-2-sulfonamide* (**5b**): 31% yield as a yellowish solid. ^1^H-NMR (400 MHz, Chloroform-*d*) δ 7.47 (d, *J* = 3.9 Hz, 1H), 7.18 (d, *J* = 3.9 Hz, 1H), 7.12–7.02 (m, 2H), 6.99–6.87 (m, 2H), 5.27 (s, 1H), 4.12 (t, *J* = 5.1 Hz, 2H), 3.60–3.39 (m, 3H). ^13^C-NMR (150 MHz, Chloroform-*d*) δ 152.7 (d, *J* = 246.0 Hz), 145.9 (d, *J* = 10.2 Hz), 141.6, 132.8, 131.5, 128.6, 124.5 (d, *J* = 4.5 Hz), 122.4 (d, *J* = 7.0 Hz), 116.4 (d, *J* = 18.4 Hz), 115.6, 84.7, 75.1, 68.0, 42.8.

*5-Ethynyl-N-(2-(o-tolyloxy)ethyl)thiophene-2-sulfonamide* (**5c**): 42% yield as a yellowish solid. ^1^H-NMR (400 MHz, Chloroform-*d*) δ 7.48 (d, *J* = 3.9 Hz, 1H), 7.19 (d, *J* = 3.9 Hz, 1H), 7.14 (dt, *J* = 7.3, 3.6 Hz, 2H), 6.90 (t, *J* = 7.3 Hz, 1H), 6.73 (d, *J* = 8.2 Hz, 1H), 5.08 (t, *J* = 5.7 Hz, 1H), 4.05 (t, *J* = 5.1 Hz, 2H), 3.55–3.38 (m, 3H), 2.18 (s, 3H). ^13^C-NMR (100 MHz, Chloroform-*d*) δ 156.0, 141.5, 132.8, 131.6, 130.9, 128.7, 126.9, 126.7, 121.3, 111.2, 84.8, 75.2, 66.2, 43.1, 16.2.

*N-(2-(2-Bromophenoxy)ethyl)-5-ethynylthiophene-2-sulfonamide* (**5d**): 27% yield as a yellowish solid. ^1^H-NMR (400 MHz, Chloroform-*d*) δ 7.53 (dd, *J* = 7.9, 1.5 Hz, 1H), 7.48 (d, *J* = 3.9 Hz, 1H), 7.28–7.22 (m, 1H), 7.17 (d, *J* = 3.9 Hz, 1H), 6.89 (td, *J* = 7.8, 1.3 Hz, 1H), 6.83 (dd, *J* = 8.2, 1.1 Hz, 1H), 5.24 (t, *J* = 5.7 Hz, 1H), 4.11 (t, *J* = 5.0 Hz, 2H), 3.55–3.46 (m, 3H). ^13^C-NMR (100 MHz, Chloroform-*d*) δ 154.3, 141.4, 133.4, 132.9, 131.6, 128.7, 128.6, 123.0, 113.9, 112.4, 84.9, 75.2, 67.6, 42.8.

*N-(2-(2-Chlorophenoxy)ethyl)-5-ethynylthiophene-2-sulfonamide* (**5e**): 29% yield as a yellowish solid. ^1^H-NMR (400 MHz, Chloroform-*d*) δ 7.47 (d, *J* = 3.9 Hz, 1H), 7.36 (dd, *J* = 7.9, 1.5 Hz, 1H), 7.24–7.15 (m, 2H), 6.95 (td, *J* = 7.8, 1.3 Hz, 1H), 6.86 (dd, *J* = 8.2, 1.1 Hz, 1H), 5.17 (t, *J* = 5.8 Hz, 2H), 4.11 (t, *J* = 5.0 Hz, 2H), 3.54–3.47 (m, 3H). ^13^C-NMR (100 MHz, Chloroform-*d*) δ 153.5, 141.5, 132.9, 131.6, 130.5, 128.7, 127.9, 123.2, 122.6, 114.1, 84.8, 75.1, 67.6, 42.9.

*N-(2-(3-Chlorophenoxy)ethyl)-5-ethynylthiophene-2-sulfonamide* (**5f**): 33% yield as a yellowish solid. ^1^H-NMR (400 MHz, Chloroform-*d*) δ 7.47 (d, *J* = 3.9 Hz, 1H), 7.23–7.17 (m, 2H), 6.99–6.94 (m, 1H), 6.83 (t, *J* = 2.1 Hz, 1H), 6.72 (dd, *J* = 8.1, 2.1 Hz, 1H), 5.04 (s, 1H), 4.04 (t, *J* = 5.0 Hz, 2H), 3.50 (s, 1H), 3.46 (q, *J* = 5.4 Hz, 2H). ^13^C-NMR (100 MHz, Chloroform-*d*) δ 158.6, 141.4, 135.0, 132.8, 131.6, 130.4, 128.8, 121.8, 114.9, 112.9, 84.9, 75.1, 66.4, 42.8.

*N-(2-(2,3-Dimethylphenoxy)ethyl)-5-ethynylthiophene-2-sulfonamide* (**5g**): 53% yield as a yellowish solid. ^1^H-NMR (400 MHz, Chloroform-*d*) δ 7.47 (d, *J* = 3.9 Hz, 1H), 7.18 (d, *J* = 3.9 Hz, 1H), 7.03 (t, *J* = 7.9 Hz, 1H), 6.81 (d, *J* = 7.6 Hz, 1H), 6.61 (d, *J* = 8.2 Hz, 1H), 5.10 (t, *J* = 5.7 Hz, 1H), 4.03 (t, *J* = 5.1 Hz, 2H), 3.50–3.46 (m, 3H), 2.26 (s, 3H), 2.09 (s, 3H). ^13^C-NMR (100 MHz, Chloroform-*d*) δ 155.8, 141.5, 138.4, 132.8, 131.6, 128.7, 125.9, 125.2, 123.2, 109.2, 84.8, 75.2, 66.5, 43.2, 20.1, 11.7.

*5-Ethynyl-N-(2-(naphthalen-1-yloxy)ethyl)thiophene-2-sulfonamide* (**5h**): 47% yield as a yellowish solid. ^1^H-NMR (400 MHz, DMSO-*d*_6_) δ 8.51 (t, *J* = 5.7 Hz, 1H), 8.17 (d, *J* = 8.1 Hz, 1H), 7.86 (d, *J* = 8.6 Hz, 1H), 7.59 (d, *J* = 3.9 Hz, 1H), 7.51–7.45 (m, 2H), 7.42–7.36 (m, 2H), 6.91 (d, *J* = 7.5 Hz, 1H), 4.87 (s, 1H), 4.17 (t, *J* = 5.2 Hz, 2H), 3.42 (q, *J* = 5.2 Hz, 2H), 3.30 (s, 1H). ^13^C-NMR (100 MHz, DMSO-*d*_6_) δ 154.2, 143.0, 134.5, 134.0, 131.8, 127.8, 127.1, 126.9, 126.6, 125.6, 125.4, 122.4, 120.6, 105.6, 88.6, 75.8, 67.1, 42.9.

*N-(2-Phenoxyethyl)-6-((trimethylsilyl)ethynyl)pyridine-3-sulfonamide* (**5i**): 74% yield as a white solid. ^1^H-NMR (400 MHz, Chloroform-*d*) δ 9.03 (d, *J* = 1.9 Hz, 1H), 8.10 (dd, *J* = 8.2, 1.9 Hz, 1H), 7.54 (d, *J* = 8.2 Hz, 1H), 7.31–7.27 (m, 2H), 6.98 (t, *J* = 7.4 Hz, 1H), 6.79 (d, *J* = 7.9 Hz, 2H), 5.10 (t, *J* = 6.1 Hz, 1H), 4.06–3.99 (t, *J* = 5.8 Hz, 2H), 3.43 (q, *J* = 5.5 Hz, 2H), 0.29 (s, 9H). ^13^C-NMR (100 MHz, Chloroform-*d*) δ 157.8, 148.1, 146.6, 135.4, 134.9, 129.6, 127.1, 121.6, 114.4, 102.3, 99.5, 66.1, 42.7, −0.5.

#### 3.2.4. General Procedure for the Synthesis of Compound **2** and **6**

To an oven-dried round-bottom flask were added compound **1a**–**f**, or **5** (0.1 mmol), PhI(CF_3_CO_2_)_2_ (0.25 mmol), Rh_2_(OAc)_4_ (0.01 mmol), CaO (1 mmol), and CH_2_Cl_2_ (5 mL). The mixture was stirred for 5 h at room temperature and was monitored by TLC. Upon completion, the reaction solution was filtered, and the filtrate was washed with saturated aqueous Na_2_S_2_O_3_ and saturated aqueous NaCl and dried with Na_2_SO_4_. The solvent was removed under vacuum, and the residue was purified by column chromatography on silica gel with hexane-ethyl acetate (3:1 to 1:1) to afford compound **2** and **6**.

*4-(Morpholinosulfonyl)-1-oxa-4-azaspiro[4.5]deca-6,9-dien-8-one* (**2a**): 37% yield as a yellowish solid. ^1^H-NMR (400 MHz, Chloroform-*d*) δ 6.73 (d, *J* = 10.1 Hz, 2H), 6.26 (d, *J* = 10.1 Hz, 2H), 4.23 (t, *J* = 6.3 Hz, 2H), 3.79–3.61 (m, 6H), 3.25–3.20 (m, 4H). ^13^C-NMR (100 MHz, Chloroform-*d*) δ 184.9, 143.3, 129.6, 86.5, 66.2, 65.7, 47.4, 46.3. HRMS (ESI) calculated for C_12_H_16_N_2_NaO_5_S: [M + Na]^+^ 323.0678, found 323.0673.

*6-Chloro-4-(morpholinosulfonyl)-1-oxa-4-azaspiro[4.5]deca-6,9-dien-8-one* (**2b**): 42% yield as a yellowish solid. ^1^H-NMR (400 MHz, Chloroform-*d*) δ 6.82 (d, *J* = 10.0 Hz, 1H), 6.47 (d, *J* = 1.9 Hz, 1H), 6.28 (dd, *J* = 10.0, 1.9 Hz, 1H), 4.48–4.39 (m, 1H), 4.33–4.24 (m, 1H), 3.82–3.66 (m, 6H), 3.35–3.24 (m, 4H). ^13^C-NMR (100 MHz, Chloroform-*d*) δ 183.2, 152.5, 142.9, 129.9, 128.4, 87.9, 67.2, 66.3, 47.5, 46.3. HRMS (ESI) calculated for C_12_H_15_ClN_2_NaO_5_S: [M + Na]^+^: 357.0288, found 357.0282.

*7-Chloro-4-(morpholinosulfonyl)-1-oxa-4-azaspiro[4.5]deca-6,9-dien-8-one* (**2c**): 35% yield as a yellowish solid. ^1^H-NMR (400 MHz, Chloroform-*d*) δ 6.88 (d, *J* = 2.9 Hz, 1H), 6.76 (dd, *J* = 10.0, 2.9 Hz, 1H), 6.34 (d, *J* = 10.0 Hz, 1H), 4.23 (t, *J* = 6.2 Hz, 2H), 3.70 (m, 6H), 3.25–3.18 (m, 4H).^13^C-NMR (100 MHz, Chloroform-*d*) δ 177.9, 143.9, 139.5, 133.8, 128.3, 87.9, 66.2, 65.8, 47.4, 46.4. HRMS (ESI) calculated for C_12_H_15_ClN_2_NaO_5_S: [M + Na]^+^ 357.0288, found 357.0285.

*3′-(Morpholinosulfonyl)-4H-spiro[naphthalene-1,2′-oxazolidin]-4-one* (**2d**): 51% yield as a yellowish solid. ^1^H-NMR (400 MHz, Chloroform-*d*) δ 8.09 (d, *J* = 7.7 Hz, 1H), 7.65–7.61 (m, 2H), 7.51 (m, 1H), 6.98 (d, *J* = 10.3 Hz, 1H), 6.47 (d, *J* = 10.3 Hz, 1H), 4.51–4.30 (m, 2H), 3.97–3.77 (m, 2H), 3.54–3.37 (m, 4H), 3.07–2.86 (m, 4H). ^13^C-NMR (100 MHz, Chloroform-*d*) δ 183.7, 142.5, 141.2, 132.9, 131.4, 129.6, 129.5, 126.9, 126.5, 88.4, 66.0, 65.7, 47.4, 46.1. HRMS (ESI) calculated for C_16_H_19_N_2_O_5_S: [M + H]^+^ 351.1015, found 351.1009.

*4-(Thiophen-2-ylsulfonyl)-1-oxa-4-azaspiro[4.5]deca-6,9-dien-8-one* (**2e**): 32% yield as a yellowish solid. ^1^H-NMR (400 MHz, Chloroform-*d*) δ 7.65 (dd, *J* = 5.1, 1.4Hz, 1H), 7.58 (dd, *J* = 3.7, 1.4 Hz, 1H), 7.11 (dd, *J* = 5.1, 3.7 Hz, 1H), 6.54 (d, *J* = 10.1 Hz, 2H), 6.23 (d, *J* = 10.1 Hz, 2H), 4.21 (t, *J* = 6.2 Hz, 2H), 3.80 (t, *J* = 6.2 Hz, 2H). ^13^C-NMR (100 MHz, Chloroform-*d*) δ 184.6, 142.6, 139.6, 133.5, 132.7, 129.9, 127.4, 86.5, 65.5, 47.1. HRMS (ESI) calculated for C_12_H_12_NO_4_S_2_: [M + H]^+^ 298.0208, found 298.0202.

*6-Bromo-4-(thiophen-2-ylsulfonyl)-1-oxa-4-azaspiro[4.5]deca-6,9-dien-8-one* (**2f**): 50% yield as a yellowish solid. ^1^H-NMR (400 MHz, Chloroform-*d*) δ 7.66 (dd, *J* = 5.0, 1.3 Hz, 1H), 7.61 (dd, *J* = 3.8, 1.3 Hz, 1H), 7.12 (dd, *J* = 5.0, 3.8 Hz, 1H), 6.72 (d, *J* = 2.0 Hz, 1H), 6.64 (d, *J* = 10.0 Hz, 1H), 6.29 (dd, *J* = 10.0, 2.0 Hz, 1H), 4.48 (m, 1H), 4.24 (m, 1H), 3.95–3.79 (m, 2H). ^13^C-NMR (100 MHz, Chloroform-*d*) δ 182.5, 145.6, 142.9, 139.3, 134.1, 133.6, 132.9, 128.5, 127.5, 88.0, 66.9, 47.4. HRMS (ESI) calculated for C_12_H_10_BrNNaO_4_S_2_: [M + Na]^+^ 397.9132, found 397.9132.

*4-((5-Ethynylthiophen-2-yl)sulfonyl)-1-oxa-4-azaspiro[4.5]deca-6,9-dien-8-one* (**6a**): 35% yield as a yellowish solid. ^1^H-NMR (400 MHz, Chloroform-*d*) δ 7.41 (d, *J* = 3.9 Hz, 1H), 7.21 (d, *J* = 3.9 Hz, 1H), 6.54 (d, *J* = 10.1 Hz, 2H), 6.25 (d, *J* = 10.1 Hz, 2H), 4.22 (t, *J* = 6.2 Hz, 2H), 3.79 (t, *J* = 6.2 Hz, 2H), 3.54 (s, 1H). ^13^C-NMR (100 MHz, Chloroform-*d*) δ 184.6, 142.4, 139.9, 132.8, 132.6, 130.0, 129.4, 86.6, 85.5, 74.9, 65.5, 47.2.

*4-((5-Ethynylthiophen-2-yl)sulfonyl)-6-fluoro-1-oxa-4-azaspiro[4.5]deca-6,9-dien-8-one* (**6b**): 27% yield as a yellowish solid. ^1^H-NMR (400 MHz, Chloroform-*d*) δ 7.42 (d, *J* = 3.9 Hz, 1H), 7.22 (d, *J* = 3.9 Hz, 1H), 6.46 (t, *J* = 9.8 Hz, 1H), 6.25 (d, *J* = 10.8 Hz, 1H), 5.94 (dd, *J* = 12.8, 1.8 Hz, 1H), 4.39–4.25 (m, 2H), 3.88–3.81 (m, 2H), 3.55 (s, 1H). ^13^C-NMR (100 MHz, Chloroform-*d*) δ 185.8 (d, *J* = 15.8 Hz), 169.6 (d, *J* = 294.1 Hz), 140.1 (d, *J* = 3.6 Hz), 139.1, 132.7, 132.7, 129.7, 129.6 (d, *J* = 1.6 Hz), 110.5 (d, *J* = 8.3 Hz), 85.8, 85.5, 74.9, 66.9, 47.5.

*4-((5-Ethynylthiophen-2-yl)sulfonyl)-6-methyl-1-oxa-4-azaspiro[4.5]deca-6,9-dien-8-one* (**6c**): 57% yield as a yellowish solid. ^1^H-NMR (400 MHz, Chloroform-*d*) δ 7.39 (d, *J* = 3.9 Hz, 1H), 7.21 (d, *J* = 3.9 Hz, 1H), 6.43 (d, *J* = 9.9 Hz, 1H), 6.22–6.13 (m, 2H), 4.33–4.28 (m,1H), 4.23–4.17 (m, 1H), 3.88–3.76 (m, 2H), 3.55 (s, 1H), 1.95 (s, 3H). ^13^C-NMR (150 MHz, Chloroform-*d*) δ 184.6, 154.1, 141.1, 139.6, 132.6, 132.4, 129.3, 129.1, 128.9, 88.3, 85.2, 74.7, 65.8, 47.2, 17.5.

*6-Bromo-4-((5-ethynylthiophen-2-yl)sulfonyl)-1-oxa-4-azaspiro[4.5]deca-6,9-dien-8-one* (**6d**): 36% yield as a yellowish solid. ^1^H-NMR (400 MHz, Chloroform-*d*) δ 7.44 (d, *J* = 3.9 Hz, 1H), 7.22 (d, *J* = 3.9 Hz, 1H), 6.73 (d, *J* = 1.8 Hz, 1H), 6.65 (d, *J* = 9.9 Hz, 1H), 6.30 (dd, *J* = 9.9, 1.8 Hz, 1H), 4.54–4.44 (m, 1H), 4.29–4.20 (m, 1H), 3.94–3.78 (m, 2H), 3.54 (s, 1H). ^13^C-NMR (100 MHz, Chloroform-*d*) δ 182.4, 145.2, 142.7, 139.7, 134.2, 132.9, 132.7, 129.6, 128.6, 88.2, 85.5, 74.9, 66.9, 47.4.

*6-Chloro-4-((5-ethynylthiophen-2-yl)sulfonyl)-1-oxa-4-azaspiro[4.5]deca-6,9-dien-8-one* (**6e**): 31% yield as a yellowish solid. ^1^H-NMR (400 MHz, Chloroform-*d*) δ 7.43 (d, *J* = 3.9 Hz, 1H), 7.22 (d, *J* = 3.9 Hz, 1H), 6.58 (d, *J* = 10.0 Hz, 1H), 6.47 (d, *J* = 1.9 Hz, 1H), 6.28 (dd, *J* = 10.0, 1.9 Hz, 1H), 4.50–4.41 (m, 1H), 4.30–4.22 (m, 1H), 3.93–3.80 (m, 2H), 3.54 (s, 1H). ^13^C-NMR (100 MHz, Chloroform-*d*) δ 183.1, 152.0, 142.7, 139.6, 132.8, 132.7, 129.9, 129.6, 128.8, 87.8, 85.5, 74.9, 67.0, 47.5.

*7-Chloro-4-((5-ethynylthiophen-2-yl)sulfonyl)-1-oxa-4-azaspiro[4.5]deca-6,9-dien-8-one* (**6f**): 23% yield as a yellowish solid. ^1^H-NMR (400 MHz, Chloroform-*d*) δ 7.40 (d, *J* = 3.9 Hz, 1H), 7.24 (d, *J* = 3.9 Hz, 1H), 6.64 (dd, *J* = 10.0, 2.9 Hz, 1H), 6.57 (d, *J* = 2.9 Hz, 1H), 6.35 (d, *J* = 10.0 Hz, 1H), 4.28–4.16 (m, 2H), 3.89–3.80 (m, 1H), 3.77–3.70 (m, 1H), 3.56 (s, 1H). ^13^C-NMR (100 MHz, Chloroform-*d*) δ 177.5, 143.7, 139.6, 138.2, 134.5, 132.9, 132.8, 129.8, 128.6, 87.9, 85.7, 74.8, 65.7, 47.1.

*4-((5-Ethynylthiophen-2-yl)sulfonyl)-6,7-dimethyl-1-oxa-4-azaspiro[4.5]deca-6,9-dien-8-one* (**6g**): 42% yield as a yellowish solid. ^1^H-NMR (400 MHz, Chloroform-*d*) δ 7.33 (d, *J* = 3.9 Hz, 1H), 7.19 (d, *J* = 3.9 Hz, 1H), 6.43 (d, *J* = 9.9 Hz, 1H), 6.20 (d, *J* = 9.9 Hz, 1H), 4.38–4.27 (m, 1H), 4.22–4.12 (m, 1H), 3.88–3.78 (m, 2H), 3.53 (s, 1H), 1.90 (s, 3H), 1.83 (s, 3H).^13^C-NMR (100 MHz, Chloroform-*d*) δ 184.6, 147.4, 140.5, 140.1, 134.7, 132.6, 132.5, 129.2, 129.1, 89.1, 85.3, 74.9, 65.5, 47.4, 14.6, 11.3.

*3′-((5-Ethynylthiophen-2-yl)sulfonyl)-4H-spiro[naphthalene-1,2′-oxazolidin]-4-one* (**6h**): 51% yield as a yellowish solid. ^1^H-NMR (400 MHz, DMSO-*d*_6_) δ 7.92 (dd, *J* = 7.6, 1.1 Hz, 1H), 7.58–7.41 (m, 3H), 7.30 (d, *J* = 4.0 Hz, 1H), 7.13 (d, *J* = 10.2 Hz, 1H), 7.09 (d, *J* = 4.0 Hz, 1H), 6.44 (d, *J* = 10.2 Hz, 1H), 4.91 (s, 1H), 4.50–4.35 (m, 2H), 3.94–3.87 (m, 2H). ^13^C-NMR (100 MHz, DMSO-*d*_6_) δ 183.9, 144.4, 140.1, 139.8, 133.9, 133.3, 132.7, 131.3, 130.1, 129.0, 128.1, 128.0, 125.8, 89.2, 88.1, 75.5, 66.0, 47.6.

*4-((6-((Trimethylsilyl)ethynyl)pyridin-3-yl)sulfonyl)-1-oxa-4-azaspiro[4.5]deca-6,9-dien-8-one* (**6i**): 47% yield as a yellowish solid. ^1^H-NMR (400 MHz, Chloroform-*d*) δ 8.96 (d, *J* = 1.9 Hz, 1H), 8.02 (dd, *J* = 8.3, 1.9 Hz, 1H), 7.59 (d, *J* = 8.3 Hz, 1H), 6.47 (d, *J* = 10.1 Hz, 2H), 6.25 (d, *J* = 10.1 Hz, 2H), 4.21 (t, *J* = 6.1 Hz, 2H), 3.75 (t, *J* = 6.1 Hz, 2H), 0.30 (s, 9H). ^13^C-NMR (100 MHz, Chloroform-*d*) δ 184.4, 148.4, 147.1, 142.5, 135.4, 134.2, 130.0, 127.0, 102.1, 100.5, 86.6, 65.6, 47.1, −0.5.

#### 3.2.5. General Procedure for the Synthesis of Compound **7**

To a round-bottom flask were added compound **6** (0.025 mmol), azide (0.03 mmol), sodium l-ascorbate (0.0075 mmol), CuSO_4_·5H_2_O (0.005 mmol), DMSO (1 mL), and H_2_O (5 mL). The mixture was stirred for 3 h at room temperature and was monitored by TLC. Upon completion, ethyl acetate was added, and the mixture was washed with saturated aqueous NaCl and dried with Na_2_SO_4_. The solvent was removed under vacuum, and the residue was purified by column chromatography on silica gel with hexane-ethyl acetate (1:2) as eluent to afford compound **7**.

*(2R,3R,4S,5R,6R)-2-(Acetoxymethyl)-6-(4-(5-((8-oxo-1-oxa-4-azaspiro[4.5]deca-6,9-dien-4-yl)sulfonyl)thiophen-2-yl)-1H-1,2,3-triazol-1-yl)tetrahydro-2H-pyran-3,4,5-triyl triacetate* (**7a**): 58% yield as a yellowish solid. ^1^H-NMR (400 MHz, Chloroform-*d*) δ 8.04 (s, 1H), 7.52 (d, *J* = 3.9 Hz, 1H), 7.33 (d, *J* = 3.9 Hz, 1H), 6.58 (d, *J* = 10.0 Hz, 2H), 6.30–6.21 (m, 2H), 5.96–5.88 (m, 1H), 5.48–5.41 (m, 2H), 5.30–5.23 (m, 1H), 4.35 (m, 1H), 4.23 (t, *J* = 6.2 Hz, 2H), 4.17 (m, 1H), 4.08–4.00 (m, 1H), 3.83 (t, *J* = 6.2 Hz, 2H), 2.10 (s, 3H), 2.09 (s, 3H), 2.05 (s, 3H), 1.93 (s, 3H). ^13^C-NMR (150 MHz, Chloroform-*d*) δ 180.6, 166.4, 165.8, 165.3, 165.1, 138.5, 137.7, 135.6, 134.4, 130.0, 125.9, 120.0, 114.6, 82.5, 81.9, 71.4, 68.4, 66.3, 63.5, 61.5, 57.4, 43.2, 16.7, 16.5, 16.4, 16.2. HRMS (ESI) calculated for C_28_H_31_N_4_O_13_S_2_: [M + H]^+^ 695.1329, found 695.1323.

*(2R,3R,4S,5R,6R)-2-(Acetoxymethyl)-6-(4-(5-((6-fluoro-8-oxo-1-oxa-4-azaspiro[4.5]deca-6,9-dien-4-yl)sulfonyl)thiophen-2-yl)-1H-1,2,3-triazol-1-yl)tetrahydro-2H-pyran-3,4,5-triyl triacetate* (**7b**): 52% yield as a yellowish solid. ^1^H-NMR (400 MHz, DMSO-*d*_6_) δ 9.14 (s, 1H), 7.76–7.68 (m, 1H), 7.65–7.54 (m, 1H), 6.89–6.80 (m, 1H), 6.49–6.43 (m, 1H), 6.32–6.22 (m, 1H), 6.12 (m, 1H), 5.68–5.59 (m, 2H), 5.19 (m, 1H), 4.49–4.37 (m, 1H), 4.35 (m, 2H), 4.17 (m, 1H), 4.13–4.07 (m, 1H), 3.90–3.80 (m, 1H), 3.74 (m, 1H), 2.05 (s, 3H), 2.02 (s, 3H), 1.99 (s, 3H), 1.84 (m, 3H). ^13^C-NMR (100 MHz, DMSO-*d*_6_) δ 184.5, 170.5, 170.0, 169.9, 169.2, 154.1, 141.8, 141.3, 141.2, 140.1, 136.8, 135.2, 135.1, 128.8, 125.4, 122.0, 115.8, 86.6, 84.6, 79.6, 73.8, 72.4, 70.8, 67.9, 66.7, 47.2, 20.9, 20.8, 20.7, 20.4. HRMS (ESI) calculated for: C_28_H_30_FN_4_O_13_S_2_ [M + H]^+^ 713.1235, found 713.1234.

*(2R,3R,4S,5R,6R)-2-(Acetoxymethyl)-6-(4-(5-((6-methyl-8-oxo-1-oxa-4-azaspiro[4.5]deca-6,9-dien-4-yl)sulfonyl)thiophen-2-yl)-1H-1,2,3-triazol-1-yl)tetrahydro-2H-pyran-3,4,5-triyl triacetate* (**7c**): 58% yield as a yellowish solid. ^1^H-NMR (400 MHz, DMSO-*d*_6_) δ 9.13 (s, 1H), 7.67 (d, *J* = 4.0 Hz, 1H), 7.56 (d, *J* = 4.0 Hz, 1H), 6.84 (d, *J* = 9.8 Hz, 1H), 6.46 (d, *J* = 8.9 Hz, 1H), 6.23–6.15 (m, 2H), 5.69–5.57 (m, 2H), 5.23–5.14 (m, 1H), 4.47–4.41 (m, 1H), 4.33–4.22 (m, 2H), 4.21–4.08 (m, 2H), 3.79 (m,2H), 2.05 (s, 3H), 2.02 (s, 3H), 1.99 (s, 3H), 1.90 (s, 3H), 1.85 (s, 3H). ^13^C-NMR (100 MHz, DMSO-*d*_6_) δ 185.2, 170.5, 170.0, 169.9, 169.2, 155.6, 143.6, 141.3, 139.6, 137.6, 135.0, 134.9, 128.8, 125.3, 122.0, 88.3, 84.6, 79.6, 73.8, 72.4, 70.8, 67.9, 66.4, 47.5, 20.9, 20.8, 20.7, 20.4, 17.6. HRMS (ESI) calculated for C_29_H_33_N_4_O_13_S_2_: [M + H]^+^ 709.1486, found 709.1488.

*(2R,3R,4S,5R,6R)-2-(Acetoxymethyl)-6-(4-(5-((6-bromo-8-oxo-1-oxa-4-azaspiro[4.5]deca-6,9-dien-4-yl)sulfonyl)thiophen-2-yl)-1H-1,2,3-triazol-1-yl)tetrahydro-2H-pyran-3,4,5-triyl triacetate* (**7d**): 31% yield as a yellowish solid. ^1^H-NMR (400 MHz, Chloroform-*d*) δ 8.06 (s, 1H), 7.55 (m, 1H), 7.35 (m, 1H), 6.79–6.62 (m, 2H), 6.32 (m, 1H), 5.93 (m, 1H), 5.46 (m, 2H), 5.27 (m, 1H), 4.49 (m, 1H), 4.35 (m, 1H), 4.25 (m, 1H), 4.20–4.11 (m, 1H), 4.05 (m, 1H), 3.98–3.82 (m, 2H), 2.10 (s, 3H), 2.09 (s, 3H), 2.05 (s, 3H), 1.93 (s, 3H). ^13^C-NMR (100 MHz, Chloroform-*d*) δ 182.5, 170.5, 169.9, 169.4, 169.1, 145.5, 142.8, 141.7, 139.8, 138.3, 134.1, 134.0, 128.6, 124.1, 118.6, 88.1, 86.0, 75.4, 72.4, 70.4, 67.6, 66.9, 61.5, 47.5, 20.7, 20.6, 20.5, 20.2. HRMS (ESI) calculated for C_28_H_29_BrN_4_NaO_13_S_2_: [M + Na]^+^ 795.0254, found 795.0251.

*(2R,3R,4S,5R,6R)-2-(Acetoxymethyl)-6-(4-(5-((6-chloro-8-oxo-1-oxa-4-azaspiro[4.5]deca-6,9-dien-4-yl)sulfonyl)thiophen-2-yl)-1H-1,2,3-triazol-1-yl)tetrahydro-2H-pyran-3,4,5-triyl triacetate* (**7e**): 50% yield as a yellowish solid. ^1^H-NMR (400 MHz, Chloroform-*d*) δ 8.04 (s, 1H), 7.54 (dd, *J* = 3.9, 2.6 Hz, 1H), 7.34 (dd, *J* = 3.9, 2.9 Hz, 1H), 6.60 (dd, *J* = 10.0, 2.1 Hz, 1H), 6.48 (t, *J* = 2.1 Hz, 1H), 6.29 (dt, *J* = 10.0, 2.1 Hz, 1H), 5.96–5.89 (m, 2H), 5.51–5.39 (m, 1H), 5.30–5.22 (m, 1H), 4.51–4.41 (m, 1H), 4.36–4.32 (m, 1H), 4.29–4.22 (m, 1H), 4.19–4.14 (m, 1H), 4.08–3.96 (m, 1H), 3.97–3.90 (m, 1H), 3.90–3.82 (m, 1H), 2.10 (s, 3H), 2.09 (s, 3H), 2.05 (s, 3H), 1.92 (s, 3H).^13^C-NMR (150 MHz, Chloroform-*d*) δ 183.5, 170.8, 170.2, 169.7, 169.5, 152.7, 143.1, 142.1, 140.2, 138.5, 134.3, 130.2, 129.2, 124.5, 119.0, 88.1, 86.4, 75.7, 72.8, 70.7, 67.9, 67.4, 61.8, 47.9, 21.0, 20.9, 20.8, 20.6. HRMS (ESI) calculated for C_28_H_29_ClN_4_NaO_13_S_2_: [M + Na]^+^ 751.0759, found 751.0758.

*(2R,3R,4S,5R,6R)-2-(Acetoxymethyl)-6-(4-(5-((7-chloro-8-oxo-1-oxa-4-azaspiro[4.5]deca-6,9-dien-4-yl)sulfonyl)thiophen-2-yl)-1H-1,2,3-triazol-1-yl)tetrahydro-2H-pyran-3,4,5-triyl triacetate* (**7f**): 71% yield as a yellowish solid. ^1^H-NMR (400 MHz, Chloroform-*d*) δ 8.00 (s, 1H), 7.44 (m, 1H), 7.30 (m, 1H), 6.61 (m, 1H), 6.53 (m, 1H), 6.30 (m, 1H), 5.90–5.81 (m, 1H), 5.43–5.34 (m, 2H), 5.30–5.15 (m, 1H), 4.28 (m, 1H), 4.22–4.11 (m, 2H), 4.05 (m, 1H), 3.98 (m, 1H), 3.85–3.66 (m, 2H), 2.03 (s, 3H), 2.02 (s, 3H), 1.98 (s, 3H), 1.86 (s,3H). ^13^C-NMR (100 MHz, Chloroform-*d*) δ 177.6, 170.5, 169.9, 169.4, 169.1, 143.8, 141.7, 139.9, 138.4, 138.1, 134.3, 134.1, 128.6, 124.3, 118.7, 87.9, 86.0, 75.4, 72.4, 70.4, 67.6, 65.7, 61.5, 47.2, 20.7, 20.6, 20.5, 20.2. HRMS (ESI) calculated for C_28_H_29_ClN_4_NaO_13_S_2_: [M + Na]^+^ 751.0759, found 751.0755.

*(2R,3R,4S,5R,6R)-2-(Acetoxymethyl)-6-(4-(5-((6,7-dimethyl-8-oxo-1-oxa-4-azaspiro[4.5]deca-6,9-dien-4-yl)sulfonyl)thiophen-2-yl)-1H-1,2,3-triazol-1-yl)tetrahydro-2H-pyran-3,4,5-triyl triacetate* (**7g**): 47% yield as a yellowish solid. ^1^H-NMR (400 MHz, DMSO-*d*_6_) δ 9.12 (s, 1H), 7.61 (m, 1H), 7.55 (d, *J* = 3.9Hz, 1H), 6.86 (d, *J* = 7.7 Hz, 1H), 6.46 (d, *J* = 7.7 Hz, 1H), 6.22–6.15 (m, 1H), 5.70–5.56 (m, 2H), 5.18 (m, 1H), 4.48–4.39 (m, 1H), 4.34–4.21 (m, 2H), 4.21–4.08 (m,2H), 3.88–3.73 (m, 2H), 2.05 (s, 3H), 2.02 (s, 3H), 1.99 (s, 3H), 1.84 (s, 3H), 1.79 (s, 3H), 1.75 (s, 3H). ^13^C-NMR (150 MHz, DMSO-*d*_6_) δ 184.7, 170.4, 169.9, 169.8, 169.1, 148.5, 142.9, 141.3, 139.4, 137.8, 134.6, 133.7, 128.2, 125.2, 121.8, 88.8, 84.6, 73.8, 72.3, 70.7, 67.9, 65.9, 62.1, 47.5, 20.9, 20.8, 20.7, 20.4, 14.8, 11.4. HRMS (ESI) calculated for C_30_H_35_N_4_O_13_S_2_: [M + H]^+^ 723.1642, found 723.1642.

*(2R,3R,4S,5R,6R)-2-(Acetoxymethyl)-6-(4-(5-((4-oxo-4H-spiro[naphthalene-1,2′-oxazolidin]-3′-yl)sulfonyl)thiophen-2-yl)-1H-1,2,3-triazol-1-yl)tetrahydro-2H-pyran-3,4,5-triyl triacetate* (**7h**): 77% yield as a yellowish solid. ^1^H-NMR (400 MHz, DMSO-*d*_6_) δ 9.06 (d, *J* = 4.6 Hz, 1H), 7.96–7.90 (m, 1H), 7.46 m, 2H), 7.40 (t, *J* = 4.0 Hz, 1H), 7.22–7.10 (m, 2H), 6.45 (d, *J* = 9.7 Hz, 2H), 5.69–5.58 (m, 2H), 5.19 (t, *J* = 9.4 Hz, 1H), 4.50–4.35 (m, 3H), 4.22–4.09 (m, 2H), 4.03 (m, 1H), 3.93 (m, 2H), 2.09 (s, 3H), 2.05 (s, 3H), 1.99 (s, 3H), 1.85 (s, 3H). ^13^C-NMR (150 MHz, DMSO-*d*_6_) δ 183.8, 170.4, 169.9, 169.8, 169.1, 144.5, 141.2, 140.3, 139.3, 137.7, 133.7, 133.3, 131.3, 129.9, 128.9, 128.0, 125.8, 124.9, 121.7, 88.0, 84.6, 73.8, 72.3, 70.8, 67.9, 65.9, 62.2, 47.5, 20.9, 20.8, 20.7, 20.4. HRMS (ESI) calculated for C_32_H_33_N_4_O_13_S_2_: [M + H]^+^ 745.1486, found 745.1477.

*(2R,3R,4S,5S,6S)-2-(Acetoxymethyl)-6-(4-(5-((8-oxo-1-oxa-4-azaspiro[4.5]deca-6,9-dien-4-yl)sulfonyl)thiophen-2-yl)-1H-1,2,3-triazol-1-yl)tetrahydro-2H-pyran-3,4,5-triyl triacetate* (**7i**): 56% yield as a yellowish solid. ^1^H-NMR (400 MHz, Chloroform-*d*) δ 8.00 (s, 1H), 7.53 (d, *J* = 3.9 Hz, 1H), 7.35 (d, *J* = 3.9 Hz, 1H), 6.63–6.53 (m, 2H), 6.25 (d, *J* = 9.5 Hz, 2H), 6.09 (d, *J* = 3.3 Hz, 1H), 5.97 (t, *J* = 3.3 Hz, 1H), 5.90–5.84 (m, 1H), 5.37 (t, *J* = 8.5 Hz, 1H), 4.49–4.40 (m, 1H), 4.23 (t, *J* = 6.1 Hz, 2H), 4.14–4.07 (m, 1H), 4.02–3.94 (m, 1H), 3.85 (t, *J* = 6.1 Hz, 2H), 2.18 (s, 3H), 2.13–2.06 (m, 9H). ^13^C-NMR (100 MHz, Chloroform-*d*) δ 184.6, 170.5, 169.7, 169.6, 169.3, 142.7, 142.5, 139.5, 138.7, 134.1, 129.9, 129.8, 124.2, 120.6, 86.5, 83.6, 72.8, 68.6, 68.1, 66.1, 65.5, 61.4, 47.2, 20.72, 20.69, 20.6. HRMS (ESI) calculated for C_28_H_31_N_4_O_13_S_2_: [M + H]^+^ 695.1329, found 695.1312.

*(2R,3R,4S,5S,6S)-2-(Acetoxymethyl)-6-(4-(5-((7-chloro-8-oxo-1-oxa-4-azaspiro[4.5]deca-6,9-dien-4-yl)sulfonyl)thiophen-2-yl)-1H-1,2,3-triazol-1-yl)tetrahydro-2H-pyran-3,4,5-triyl triacetate* (**7j**): 54% yield as a yellowish solid. M.p. 113–116 °C. ^1^H-NMR (400 MHz, Chloroform-*d*) δ 8.00 (d, *J* = 0.9 Hz, 1H), 7.52 (dd, *J* = 3.9, 0.9 Hz, 1H), 7.38 (d, *J* = 3.9 Hz, 1H), 6.71–6.64 (m, 1H), 6.59 (dd, *J* = 7.4, 2.9 Hz, 1H), 6.35 (dd, *J* = 10.0, 2.9 Hz, 1H), 6.09 (d, *J* = 3.5 Hz, 1H), 5.98 (t, *J* = 3.5 Hz, 1H), 5.91–5.85 (m, 1H), 5.41–5.34 (m, 1H), 4.49–4.42 (m, 1H), 4.29–4.20 (m, 2H), 4.14–4.08 (m, 1H), 4.02–3.86 (m, 2H), 3.84–3.77 (m, 1H), 2.18 (s, 3H), 2.10 (s, 3H), 2.09 (m, 6H). ^13^C-NMR (150 MHz, Chloroform-*d*) δ 177.9, 170.9, 170.0, 169.9, 169.7, 144.3, 144.2, 141.9, 140.2, 138.8, 138.6, 134.5, 128.9, 124.7, 121.0, 88.2, 83.9, 73.1, 68.9, 68.4, 66.4, 66.1, 61.7, 47.6, 21.1, 20.9. HRMS (ESI) calculated for C_28_H_29_ClN_4_NaO_13_S_2_: [M + Na]^+^ 751.0759, found 751.0749.

*(2R,3R,4S,5S,6S)-2-(Acetoxymethyl)-6-(4-(5-((4-oxo-4H-spiro[naphthalene-1,2′-oxazolidin]-3′-yl)sulfonyl)thiophen-2-yl)-1H-1,2,3-triazol-1-yl)tetrahydro-2H-pyran-3,4,5-triyl triacetate* (**7k**): 66% yield as a yellowish solid. ^1^H-NMR (400 MHz, DMSO-*d*_6_) δ 8.91 (d, *J* = 1.6 Hz, 1H), 7.94 (d, *J* = 7.9 Hz, 1H), 7.54–7.45 (m, 4H), 7.20–7.12 (m, 2H), 6.48–6.43 (m, 2H), 5.89–5.86 (m,1H), 5.77–5.73 (m, 1H), 5.28 (t, *J* = 9.4 Hz, 1H), 4.50–4.45 (m, 1H), 4.42–4.36 (m,1H), 4.27–4.23 (m, 1H), 4.08–4.04 (m, 1H), 3.95–3.88 (m, 3H), 2.17 (s, 3H), 2.04 (s, 3H), 2.02 (d, *J* = 2.2 Hz, 3H), 2.01 (s, 3H). ^13^C-NMR (150 MHz, DMSO-*d*_6_) δ 184.2, 170.8, 170.3, 170.2(2C), 144.8, 141.6, 140.7, 139.6, 138.3, 134.1, 133.7, 131.7, 130.3, 129.4, 128.4, 126.1, 125.6, 123.8, 88.4, 84.2, 72.2, 69.2, 68.2, 66.3, 66.0, 62.1, 47.9, 21.34, 21.32, 21.23, 21.15. HRMS (ESI) calculated for C_32_H_33_N_4_O_13_S_2_: [M + H]^+^ 745.1486, found 745.1471.

*(2R,3R,4S,5S,6S)-2-(Acetoxymethyl)-6-(4-(5-((8-oxo-1-oxa-4-azaspiro[4.5]deca-6,9-dien-4-yl)sulfonyl)pyridin-2-yl)-1H-1,2,3-triazol-1-yl)tetrahydro-2H-pyran-3,4,5-triyl triacetate* (**7l**): 52% yield as a yellowish solid. ^1^H-NMR (400 MHz, Chloroform-*d*) δ 8.97 (s, 1H), 8.50 (s, 1H), 8.38 (d, *J* = 8.2 Hz, 1H), 8.19 (d, *J* = 8.2 Hz, 1H), 6.51 (d, *J* = 9.6 Hz, 2H), 6.27 (d, *J* = 9.6 Hz, 2H), 6.12 (m, 1H), 6.02 (m, 1H), 5.89 (m, 1H), 5.38 (m, 1H), 4.42 (m, 1H), 4.22 (t, *J* = 6.1 Hz, 2H), 4.08 (m, 1H), 3.96 (t, *J* = 6.1 Hz, 2H), 3.81–3.72 (m, 1H), 2.19 (s, 3H), 2.10 (s, 3H), 2.08 (s, 3H), 2.07 (s, 3H). ^13^C-NMR (100 MHz, Chloroform-*d*) δ 184.4, 170.5, 169.7, 169.6, 169.4, 153.6, 148.5, 147.4, 142.7, 142.6, 136.3, 134.5, 129.9, 123.9, 120.1, 86.6, 83.8, 72.6, 68.6, 68.1, 66.1, 65.6, 61.4, 47.1, 20.72, 20.70, 20.68, 20.6. HRMS (ESI) calculated for C_29_H_31_N_5_NaO_13_S: [M + Na]^+^ 712.1537, found 712.1530.

*(2R,3R,4S,5R,6R)-2-(Acetoxymethyl)-6-(4-(5-((8-oxo-1-oxa-4-azaspiro[4.5]deca-6,9-dien-4-yl)sulfonyl)pyridin-2-yl)-1H-1,2,3-triazol-1-yl)tetrahydro-2H-pyran-3,4,5-triyl triacetate* (**7m**): 45% yield as a yellowish solid. ^1^H-NMR (400 MHz, Chloroform-*d*) δ 8.99 (d, *J* = 2.0 Hz, 1H), 8.51 (s, 1H), 8.31 (d, *J* = 8.3 Hz, 1H), 8.15 (dd, *J* = 8.3, 2.0 Hz, 1H), 6.57–6.47 (m, 2H), 6.33–6.25 (m, 2H), 5.98–5.91 (m, 1H), 5.51–5.43 (m, 2H), 5.31–5.24 (m, 1H), 4.38–4.30 (m, 1H), 4.26–4.15 (m, 3H), 4.09–4.01 (m, 1H), 3.77 (t, *J* = 6.1 Hz, 2H), 2.11 (s, 3H), 2.09 (s, 3H), 2.05 (s, 3H), 1.92 (s, 3H). ^13^C-NMR (150 MHz, Chloroform-*d*) δ 184.5, 170.5, 169.9, 169.3, 168.9, 153.6, 148.4, 147.4, 142.7, 142.6, 136.2, 134.3, 129.9, 122.3, 119.9, 86.5, 85.9, 75.2, 72.4, 70.6, 67.6, 65.6, 61.5, 47.0, 20.7, 20.5, 20.5, 20.2. HRMS (ESI) calculated for C_29_H_31_N_5_NaO_13_S: [M + Na]^+^ 712.1537, found 712.1529.

#### 3.2.6. General Procedure for the Synthesis of Compound **8**

To a round-bottom flask were added acetyl glycosyl triazole compound **7** (0.02 mmol), K_2_CO_3_ (0.002 mmol), CH_3_OH (1 mL). The mixture was stirred 0.5 h at room temperature and was monitored by TLC. Upon completion, the reaction solution was purified by column chromatography on silica gel with chloroform-methanol (15:1) as eluent to afford compound **8**.

*4-((5-(1-((2R,3R,4S,5S,6R)-3,4,5-Trihydroxy-6-(hydroxymethyl)tetrahydro-2H-pyran-2-yl)-1H-1,2,3-triazol-4-yl)thiophen-2-yl)sulfonyl)-1-oxa-4-azaspiro[4.5]deca-6,9-dien-8-one* (**8a**): 72% yield as a white solid. ^1^H-NMR (400 MHz, DMSO-*d*_6_) δ 9.02 (s, 1H), 7.69 (d, *J* = 3.9 Hz, 1H), 7.56 (d, *J* = 3.9 Hz, 1H), 6.81 (d, *J* = 10.2 Hz, 2H), 6.24 (d, *J* = 10.2 Hz, 2H), 5.62 (m, 1H), 5.53 (m, 1H), 5.41 (m, 1H), 5.25 (m, 1H), 4.69 (m, 1H), 4.23 (t, *J* = 6.2 Hz, 2H), 3.81–3.73 (m, 2H), 3.71 (m, 1H), 3.54–3.44 (m, 4H), 3.16 (m, 1H). ^13^C-NMR (100 MHz, DMSO-*d*_6_) δ 185.3, 144.4, 140.7, 140.4, 137.3, 134.9, 129.5, 124.9, 122.1, 88.3, 86.5, 80.5, 77.2, 72.8, 70.0, 65.9, 61.2, 47.4. HRMS (ESI) calculated for C_20_H_22_N_4_NaO_9_S_2_: [M + Na]^+^ 549.0726, found 549.0727.

*7-Chloro-4-((5-(1-((2R,3R,4S,5S,6R)-3,4,5-trihydroxy-6-(hydroxymethyl)tetrahydro-2H-pyran-2-yl)-1H-1,2,3-triazol-4-yl)thiophen-2-yl)sulfonyl)-1-oxa-4-azaspiro[4.5]deca-6,9-dien-8-one* (**8b**): 82% yield as a white solid. ^1^H-NMR (400 MHz, DMSO-*d*_6_) δ 9.02 (s, 1H), 7.67 (d, *J* = 3.9 Hz, 1H), 7.58 (d, *J* = 3.9 Hz, 1H), 7.21 (s, 1H), 6.96–6.86 (m, 2H), 6.42 (d, *J* = 10.0 Hz, 1H), 5.62 (m, 1H), 5.53 (m, 1H), 5.40 (m,1H), 5.24 (m, 1H), 4.68 (m, 1H), 4.32–4.23 (m, 2H), 4.14 (m, 2H), 3.83–3.75 (m, 2H), 3.75–3.66 (m, 4H). ^13^C-NMR (100 MHz, DMSO-*d*_6_) δ 178.1, 145.7, 140.9, 140.8, 140.7, 136.9, 134.9, 132.8, 128.5, 124.9, 122.1, 88.3, 80.5, 79.6, 77.2, 72.8, 70.0, 66.2, 61.2, 47.3. HRMS (ESI) calculated for C_20_H_22_ClN_4_O_9_S_2_: [M + H]^+^ 561.0517, found 561.0506.

*3′-((5-(1-((2R,3R,4S,5S,6R)-3,4,5-Trihydroxy-6-(hydroxymethyl)tetrahydro-2H-pyran-2-yl)-1H-1,2,3-triazol-4-yl)thiophen-2-yl)sulfonyl)-4H-spiro[naphthalene-1,2′-oxazolidin]-4-one* (**8c**): 90% yield as a white solid. ^1^H-NMR (400 MHz, DMSO-*d*_6_) δ 8.96 (s, 1H), 7.93 (d, *J* = 7.4 Hz, 1H), 7.52 (m, 1H), 7.46 (m, 2H), 7.39 (t, *J* = 3.6 Hz, 1H), 7.17–7.09 (m, 2H), 6.44 (d, *J* = 10.2 Hz, 1H), 5.61 (m, 1H), 5.50 (m, 1H), 5.37 (m, 1H), 5.21 (m, 1H), 4.65 (m, 1H), 4.51–4.33 (m, 2H), 3.97–3.89 (m, 2H), 3.81–3.67 (m, 2H), 3.55–3.40 (m, 4H). ^13^C-NMR (150 MHz, DMSO-*d*_6_) δ 183.9, 144.5, 140.7, 140.2, 140.0, 137.2, 133.8, 133.3, 131.3, 129.9, 128.9, 128.0, 125.7, 124.5, 121.9, 88.2, 87.9, 80.4, 77.1, 72.8, 69.9, 65.9, 61.2, 47.5. HRMS (ESI) calculated for C_24_H_25_N_4_O_9_S_2_: [M + H]^+^ 577.1063, found 577.1057.

*4-((5-(1-((2R,3S,4S,5S,6R)-3,4,5-Trihydroxy-6-(hydroxymethyl)tetrahydro-2H-pyran-2-yl)-1H-1,2,3-triazol-4-yl)thiophen-2-yl)sulfonyl)-1-oxa-4-azaspiro[4.5]deca-6,9-dien-8-one* (**8d**): 61% yield as a white solid. ^1^H-NMR (400 MHz, DMSO-*d*_6_) δ 8.93 (s, 1H), 7.69 (d, *J* = 4.0 Hz, 1H), 7.60 (d, *J* = 4.0 Hz, 1H), 6.82 (d, *J* = 10.1 Hz, 2H), 6.24 (d, *J* = 10.1 Hz, 2H), 5.96 (m, 1H), 5.38 (m, 1H), 5.18 (m, 1H), 5.10 (m, 1H), 4.65 (m, 1H), 4.44 (m, 1H), 4.23 (t, *J* = 6.2 Hz, 2H), 3.87–3.81 (m, 1H), 3.76 (t, *J* = 6.2 Hz, 2H), 3.61 (m, 4H). ^13^C-NMR (100 MHz, DMSO-*d*_6_) δ 185.2, 144.4, 140.7, 140.4, 137.3, 134.9, 129.5, 129.4, 125.0, 122.6, 86.6, 86.5, 79.2, 71.8, 68.4, 68.3, 65.9, 61.1, 47.4. HRMS (ESI) calculated for C_20_H_22_N_4_NaO_9_S_2_: [M + Na]^+^ 549.0726, found 549.0729.

*7-Chloro-4-((5-(1-((2R,3S,4S,5S,6R)-3,4,5-trihydroxy-6-(hydroxymethyl)tetrahydro-2H-pyran-2-yl)-1H-1,2,3-triazol-4-yl)thiophen-2-yl)sulfonyl)-1-oxa-4-azaspiro[4.5]deca-6,9-dien-8-one* (**8e**): 64% yield as a white solid. ^1^H-NMR (400 MHz, DMSO-*d*_6_) δ 8.93 (s, 1H), 7.64 (m,2H), 7.26–7.16 (m, 1H), 6.92 (d, *J* = 9.9 Hz,1H), 6.42 (d, *J* = 9.9 Hz, 1H), 5.96 (m, 1H), 5.47–5.30 (m, 1H), 5.18 (m, 1H), 5.12–5.02 (m, 1H), 4.72–4.59 (m,1H), 4.44 (m, 1H), 4.27 (m, 2H), 3.84 (m, 1H), 3.77 (m, 2H), 3.69–3.54 (m, 4H). ^13^C-NMR (100 MHz, DMSO-*d*_6_) δ 178.2, 145.7, 140.9, 140.8, 137.0, 135.0, 134.9, 132.8, 128.5, 125.1, 122.7, 88.0, 86.5, 79.6, 79.2, 71.8, 68.4, 68.2, 66.2, 47.3. HRMS (ESI) calculated for C_20_H_21_ClN_4_NaO_9_S_2_: [M + Na]^+^ 583.0336, found 583.0331.

*3′-((5-(1-((2R,3S,4S,5S,6R)-3,4,5-Trihydroxy-6-(hydroxymethyl)tetrahydro-2H-pyran-2-yl)-1H-1,2,3-triazol-4-yl)thiophen-2-yl)sulfonyl)-4H-spiro[naphthalene-1,2′-oxazolidin]-4-one* (**8f**): 73% yield as a white solid. ^1^H-NMR (400 MHz, DMSO-*d*_6_) δ 8.87 (s, 1H), 7.93 (d, *J* = 7.4 Hz, 1H), 7.47 (m, 4H), 7.29–7.04 (m, 2H), 6.44 (d, *J* = 10.1 Hz, 1H), 5.96 (m, 1H), 5.39 (m, 1H), 5.19 (m, 1H), 5.11 (m, 1H), 4.73–4.59 (m, 1H), 4.52–4.41 (m, 2H), 4.38 (m, 1H), 3.97–3.90 (m, 2H), 3.88–3.79 (m, 1H), 3.70–3.52 (m, 4H). ^13^C-NMR (100 MHz, DMSO-*d*_6_) δ 183.9, 144.5, 140.7, 140.4, 140.1, 137.3, 133.8, 133.3, 131.3, 129.9, 129.0, 128.1, 125.8, 124.7, 122.5, 88.1, 86.5, 79.2, 71.8, 68.4, 68.3, 65.9, 61.1, 47.5. HRMS (ESI) calculated for C_24_H_25_N_4_O_9_S_2_: [M + H]^+^ 577.1063, found 577.1061.

*4-((6-(1-((2R,3R,4S,5S,6R)-3,4,5-Trihydroxy-6-(hydroxymethyl)tetrahydro-2H-pyran-2-yl)-1H-1,2,3-triazol-4-yl)pyridin-3-yl)sulfonyl)-1-oxa-4-azaspiro[4.5]deca-6,9-dien-8-one* (**8g**): 69% yield as a white solid. ^1^H-NMR (400 MHz, DMSO-*d*_6_) δ 9.02 (s, 1H), 8.91 (s, 1H), 8.28 (m, 2H), 6.85–6.72 (m, 2H), 6.23 (d, *J* = 10.0 Hz, 2H), 5.65 (m, 1H), 5.49 (m, 1H), 5.35 (m, 1H), 5.22 (m, 1H), 4.67 (m, 1H), 4.21 (t, *J* = 6.2 Hz, 2H), 3.85 (m, 1H), 3.77 (t, *J* = 6.2 Hz, 2H), 3.75–3.69 (m, 1H), 3.52–3.41 (m, 4H). ^13^C-NMR (100 MHz, DMSO-*d*_6_) δ 185.2, 153.9, 148.5, 146.3, 144.8, 144.7, 137.2, 134.2, 129.4, 124.8, 120.4, 88.3, 86.4, 80.5, 77.3, 72.6, 69.9, 65.9, 61.3, 47.3. HRMS (ESI) calculated for C_21_H_23_N_5_NaO_9_S: [M + Na]^+^ 544.1114, found 544.1114.

*4-((6-(1-((2R,3S,4S,5S,6R)-3,4,5-Trihydroxy-6-(hydroxymethyl)tetrahydro-2H-pyran-2-yl)-1H-1,2,3-triazol-4-yl)pyridin-3-yl)sulfonyl)-1-oxa-4-azaspiro[4.5]deca-6,9-dien-8-one* (**8h**): 77% yield as a white solid. ^1^H-NMR (400 MHz, DMSO-*d*_6_) δ 8.93 (s, 1H), 8.91 (s, 1H), 8.31–8.22 (m, 2H), 6.80 (d, *J* = 8.1 Hz, 2H), 6.24 (d, *J* = 8.1 Hz, 2H), 6.04 (m, 1H), 5.40 (m, 1H), 5.15 (m, 1H), 5.07 (m, 1H), 4.70 (m, 1H), 4.55–4.45 (m, 1H), 4.21 (t, *J* = 6.2 Hz, 2H), 3.88–3.82 (m, 1H), 3.77 (t, *J* = 6.2 Hz, 2H), 3.64 (m, 1H), 3.56 (m, 3H). ^13^C-NMR (100 MHz, DMSO-*d*_6_) δ 185.2, 153.9, 148.5, 146.3, 144.8, 137.3, 134.3, 129.4, 125.2, 120.4, 86.7, 86.5, 79.0, 71.8, 68.5, 68.3, 65.9, 61.3, 47.3. HRMS (ESI) calculated for C_21_H_23_N_5_NaO_9_S: [M + Na]^+^ 544.1114, found 544.1118.

#### 3.2.7. In Vitro Antiproliferative Assay

A549, HeLa, and MDA-MB-231 cell lines were initially purchased from the American Type Culture Collection (ATCC, Manassas, VA, USA,). For growth assay in the presence of sulfonylazaspirocyclodienone derivatives, the cancer cells were plated in plates at a density of 50,000 each well in 5% FBS DMEM medium. The cells were then treated with sulfonylazaspirocyclodienone derivatives separately at 6 different doses for 4 days, while equal treatment volume of DMSO and HL-X9 was used as vehicle control and positive control, respectively. Cell numbers were counted with a Coulter instrument (Beckman-Coulter). The ratio of drug-treated cell numbers to vehicle-treated cell numbers was defined as the survival ratio. IC_50_ values were obtained from dose-response curves for each sulfonylazaspirocyclodienone derivatives. Experiments were conducted in triplicate, and data represented as mean ± SD.

#### 3.2.8. Cell Cycle Assay

MDA-MB-231 Cells (5 × 10^5^ cells/mL) were seeded in 6-well plates and treated with compounds at different concentrations for 24 h. The cells were then harvested by trypsinization and washed twice with cold PBS. After centrifugation and removal of the supernatants, cells were resuspended in 400 μL of 1 × PBS buffer. After adding 10 μL of PI the cells were incubated at room temperature for 15 min in the dark. The stained cells were analyzed by a flow cytometer (BD Accuri C6, BD Biosciences, San Jose, CA, USA).

#### 3.2.9. Apoptosis Detection Assay

MDA-MB-231 cells (5 × 10^5^ cells/mL) were seeded in 6-well plates and treated with compounds at different concentrations for 24 h. The cells were then harvested by trypsinization and washed twice with cold PBS. After centrifugation and removal of the supernatants, cells were resuspended in 400 μL of 1 × binding buffer, which was then added to 5 μL of annex in V-FITC and incubated at room temperature for 15 min. After adding 10 μL of PI the cells were incubated at room temperature for another 15 min in the dark. The stained cells were analyzed by a flow cytometer (BD Accuri C6).

#### 3.2.10. Acute Toxicity Experiments

Each group included 10 KM mice (~20 g), sex in half. Compounds HL-X9 or **7j** were administrated as a single i.p. injection of 0.2 mL solutions of different concentrations prepared immediately prior to use by dissolving compounds in DMSO (330 μL), tween80 (70 μL), and 0.9% NaCl (2.1 mL). Animals were observed daily for evidence of toxicity and survival within 14 days after administration. At the end of the experiment, all surviving animals were sacrificed by cervical dislocation and dissected to observe the changes of main organs (heart, liver, spleen, lung, and kidney).

All animal experiments were conducted under a protocol (Protocol SYXK2013-113) approved by Sichuan University Animal Care and Use Committee. All animal procedures are in compliance with the guidelines established by the Institutional Ethics Committee of Sichuan University.

## 4. Conclusions

We have designed and synthesized a series of novel 4-(aromatic sulfonyl)-1-oxa-4-azaspiro[4.5]deca-6,9-dien-8-ones by optimizing the lead compound HL-X9 for improved efficacy. The preliminary antiproliferative assay showed that most of the sulfonylazaspirodienone derivatives have moderate to potent activity against A549, MDA-MB-231, and HeLa cell lines. The acetyl-protected mannose-linked sulfonylazaspirodienones (**7i**–**7l**) have been greatly improved and are more potent than the lead HL-X9. Among them, **7j** is the most potent derivative with IC_50_ values of 0.17 µM, 0.05 µM, and 0.07 µM against A549, MDA-MB-231, and HeLa cell lines, respectively. The efficacy assay of **7j** in vivo is undergoing.

## Figures and Tables

**Table 1:** Antiproliferation activity of sulfonylazaspirodienones against cancer cell lines in vitro.

NO.	IC_50_ (μM)		
	A549	MDA-MB-231	HeLa
HL-X9	0.33 ± 0.09	0.39 ± 0.02	0.71 ± 0.06
**2a**	0.29 ± 0.08	0.19 ± 0.02	0.32 ± 0.03
**2b**	5.28 ± 0.14	0.24 ± 0.15	4.91 ± 0.47
**2c**	0.24 ± 0.04	0.07 ± 0.02	0.36 ± 0.06
**2d**	>10	0.52 ± 0.25	>10
**2e**	0.32 ± 0.02	0.12 ± 0.02	0.22 ± 0.03
**2f**	1.22 ± 0.39	0.33 ± 0.06	0.22 ± 0.04
**7a**	0.68 ± 0.02	0.15 ± 0.03	0.20 ± 0.02
**7b**	4.60 ± 0.10	0.72 ± 0.02	1.22 ± 0.40
**7c**	>10	>10	>10
**7d**	2.43 ± 0.95	0.08 ± 0.00	1.38 ± 0.52
**7e**	0.33 ± 0.11	0.18 ± 0.03	0.21 ± 0.07
**7f**	0.24 ± 0.07	0.05 ± 0.00	0.19 ± 0.02
**7g**	>10	3.39 ± 0.32	>10
**7h**	0.42 ± 0.01	0.08 ± 0.01	0.58 ± 0.09
**7i**	0.20 ± 0.01	0.10 ± 0.08	0.19 ± 0.01
**7j**	0.17 ± 0.08	0.05 ± 0.00	0.07 ± 0.01
**7k**	0.40 ± 0.03	0.08 ± 0.00	0.32 ± 0.07
**7l**	0.24 ± 0.03	0.08 ± 0.00	0.28 ± 0.09
**8a**	4.69 ± 2.90	3.28 ± 1.30	>10
**8b**	>10	>10	6.89 ± 0.53
**8c**	>10	>10	>10
**8d**	>10	5.05 ± 1.50	9.59 ± 3.60
**8e**	>10	1.50 ± 0.74	>10
**8f**	8.87 ± 0.52	0.54 ± 0.24	7.23 ± 0.50
**8g**	0.61 ± 0.12	>10	8.96 ± 1.60
**8h**	1.87 ± 0.97	>10	>10

± SD in triplicate.

## Supplementary Materials

The following are available online, ^1^H-NMR and ^13^C-NMR Spectra.
