# Supplementary material for: Design, Synthesis, and Antitumor Activity of a Series of Novel 4-(Aromatic Sulfonyl)-1-oxa-4-azaspiro[4.5]deca-6,9-dien-8-ones
Source: Molecules. 2020 Nov 21;25(22):5459. doi: 10.3390/molecules25225459 (PMC7700525; doi:10.3390/molecules25225459)

Design, Synthesis, and Antitumor Activity of a Series of Novel 4-(Aromatic Sulfonyl)-1-oxa-4-azaspiro[4.5] deca-6,9-dien-8-ones

Naiguo Xing [1,2], Chen Chen [1,2], Qiu Zhong [3], Shilong Zheng [3], Guangdi Wang [3,*] and Ling He [1,2,*]

1 Key Laboratory of Drug-Targeting and Drug Delivery System of the Education Ministry, Department of Medicinal Chemistry, West China School of Pharmacy, Sichuan University, Chengdu, Sichuan 610041, China; xingng1982@163.com (N.X.); cc_45@live.cn (C.C.)

2 Sichuan Engineering Laboratory for Plant–Sourced Drug and Sichuan Research Center for Drug Precision Industrial Technology, Department of Medicinal Chemistry, West China School of Pharmacy, Sichuan University, Chengdu 610041, Sichuan, China

3 RCMI Cancer Research Center and Department of Chemistry, Xavier University of Louisiana, New Orleans, LA 70125, USA; qzhong@xula.edu (Q.Z.); szheng@xula.edu (S.Z.)

* Correspondence: gwang@xula.edu (G.W.); heling2012@scu.edu.cn (L.H.)

Table of Contents

^{1}H-NMR and ^{13}C-NMR Spectra

N-(2-Phenoxyethyl)morpholine-4-sulfonamide (1a)

N-(2-(2-Chlorophenoxy)ethyl)morpholine-4-sulfonamide (1b)

N-(2-(3-Chlorophenoxy)ethyl)morpholine-4-sulfonamide (1c)

N-(2-(Naphthalen-1-yloxy)ethyl)morpholine-4-sulfonamide (1d)

N-(2-Phenoxyethyl)thiophene-2-sulfonamide (1e)

N-(2-(2-Bromophenoxy)ethyl)thiophene-2-sulfonamide (1f)

N-(2-Phenoxyethyl)-6-chloropyridine-3-sulfonamide (1g)

5-Ethynyl-N-(2-phenoxyethyl)thiophene-2-sulfonamide (5a)

5-Ethynyl-N-(2-(2-fluorophenoxy)ethyl)thiophene-2-sulfonamide (5b)

5-Ethynyl-N-(2-(o-tolyloxy)ethyl)thiophene-2-sulfonamide (5c)

N-(2-(2-Bromophenoxy)ethyl)-5-ethynylthiophene-2-sulfonamide (5d)

N-(2-(2-Chlorophenoxy)ethyl)-5-ethynylthiophene-2-sulfonamide (5e)

N-(2-(3-Chlorophenoxy)ethyl)-5-ethynylthiophene-2-sulfonamide (5f)

N-(2-(2,3-Dimethylphenoxy)ethyl)-5-ethynylthiophene-2-sulfonamide (5g)

5-Ethynyl-N-(2-(naphthalen-1-yloxy)ethyl)thiophene-2-sulfonamide (5h)

N-(2-Phenoxyethyl)-6-((trimethylsilyl)ethynyl)pyridine-3-sulfonamide (5i)

4-(Morpholinosulfonyl)-1-oxa-4-azaspiro[4.5]deca-6,9-dien-8-one (2a)

6-Chloro-4-(morpholinosulfonyl)-1-oxa-4-azaspiro[4.5]deca-6,9-dien-8-one (2b)

7-Chloro-4-(morpholinosulfonyl)-1-oxa-4-azaspiro[4.5]deca-6,9-dien-8-one (2c)

3'-(Morpholinosulfonyl)-4H-spiro[naphthalene-1,2'-oxazolidin]-4-one (2d)

4-(Thiophen-2-ylsulfonyl)-1-oxa-4-azaspiro[4.5]deca-6,9-dien-8-one (2e)

6-Bromo-4-(thiophen-2-ylsulfonyl)-1-oxa-4-azaspiro[4.5]deca-6,9-dien-8-one (2f)

4-((5-Ethynylthiophen-2-yl)sulfonyl)-1-oxa-4-azaspiro[4.5]deca-6,9-dien-8-one (6a)

4-((5-Ethynylthiophen-2-yl)sulfonyl)-6-fluoro-1-oxa-4-azaspiro[4.5]deca-6,9-dien-8-one (6b)

4-((5-Ethynylthiophen-2-yl)sulfonyl)-6-methyl-1-oxa-4-azaspiro[4.5]deca-6,9-dien-8-one (6c)

6-Bromo-4-((5-ethynylthiophen-2-yl)sulfonyl)-1-oxa-4-azaspiro[4.5]deca-6,9-dien-8-one (6d)

6-Chloro-4-((5-ethynylthiophen-2-yl)sulfonyl)-1-oxa-4-azaspiro[4.5]deca-6,9-dien-8-one (6e)

7-Chloro-4-((5-ethynylthiophen-2-yl)sulfonyl)-1-oxa-4-azaspiro[4.5]deca-6,9-dien-8-one (6f)

4-((5-Ethynylthiophen-2-yl)sulfonyl)-6,7-dimethyl-1-oxa-4-azaspiro[4.5]deca-6,9-dien-8-one (6g)

3'-((5-Ethynylthiophen-2-yl)sulfonyl)-4H-spiro[naphthalene-1,2'-oxazolidin]-4-one (6h)

4-((6-((Trimethylsilyl)ethynyl)pyridin-3-yl)sulfonyl)-1-oxa-4-azaspiro[4.5]deca-6,9-dien-8-one (6i)

(2R,3R,4S,5R,6R)-2-(Acetoxymethyl)-6-(4-(5-((8-oxo-1-oxa-4-azaspiro[4.5]deca-6,9-dien-4-yl)sulfonyl)thiophen-2-yl)-1*H*-1,2,3-triazol-1-yl)tetrahydro-2*H*-pyran-3,4,5-triyl triacetate (7a)

(2R,3R,4S,5R,6R)-2-(Acetoxymethyl)-6-(4-(5-((6-fluoro-8-oxo-1-oxa-4-azaspiro[4.5]deca-6,9-dien-4-yl)sulfonyl)thiophen-2-yl)-1*H*-1,2,3-triazol-1-yl)tetrahydro-2*H*-pyran-3,4,5-triyl triacetate (7b)

(2R,3R,4S,5R,6R)-2-(Acetoxymethyl)-6-(4-(5-((6-methyl-8-oxo-1-oxa-4-azaspiro[4.5]deca-6,9-dien-4-yl)sulfonyl)thiophen-2-yl)-1*H*-1,2,3-triazol-1-yl)tetrahydro-2*H*-pyran-3,4,5-triyl triacetate (7c)

(2R,3R,4S,5R,6R)-2-(Acetoxymethyl)-6-(4-(5-((6-bromo-8-oxo-1-oxa-4-azaspiro[4.5]deca-6,9-dien-4-yl)sulfonyl)thiophen-2-yl)-1*H*-1,2,3-triazol-1-yl)tetrahydro-2*H*-pyran-3,4,5-triyl triacetate (7d)

(2R,3R,4S,5R,6R)-2-(Acetoxymethyl)-6-(4-(5-((6-chloro-8-oxo-1-oxa-4-azaspiro[4.5]deca-6,9-dien-4-yl)sulfonyl)thiophen-2-yl)-1*H*-1,2,3-triazol-1-yl)tetrahydro-2*H*-pyran-3,4,5-triyl triacetate (7e)

(2R,3R,4S,5R,6R)-2-(Acetoxymethyl)-6-(4-(5-((7-chloro-8-oxo-1-oxa-4-azaspiro[4.5]deca-6,9-dien-4-yl)sulfonyl)thiophen-2-yl)-1*H*-1,2,3-triazol-1-yl)tetrahydro-2*H*-pyran-3,4,5-triyl triacetate (7f)

(2R,3R,4S,5R,6R)-2-(Acetoxymethyl)-6-(4-(5-((6,7-dimethyl-8-oxo-1-oxa-4-azaspiro[4.5]deca-6,9-dien-4-yl)sulfonyl)thiophen-2-yl)-1*H*-1,2,3-triazol-1-yl)tetrahydro-2*H*-pyran-3,4,5-triyl triacetate (7g)

(2R,3R,4S,5R,6R)-2-(Acetoxymethyl)-6-(4-(5-((4-oxo-4*H*-spiro[naphthalene-1,2'-oxazolidin]-3'-yl)sulfonyl)thiophen-2-yl)-1*H*-1,2,3-triazol-1-yl)tetrahydro-2*H*-pyran-3,4,5-triyl triacetate (7h)

(2R,3R,4S,5S,6S)-2-(Acetoxymethyl)-6-(4-(5-((8-oxo-1-oxa-4-azaspiro[4.5]deca-6,9-dien-4-yl)sulfonyl)thiophen-2-yl)-1H-1,2,3-triazol-1-yl)tetrahydro-2H-pyran-3,4,5-triyl triacetate (7i)

(2R,3R,4S,5S,6S)-2-(Acetoxymethyl)-6-(4-(5-((7-chloro-8-oxo-1-oxa-4-azaspiro[4.5]deca-6,9-dien-4-

yl)sulfonyl)thiophen-2-yl)-1H-1,2,3-triazol-1-yl)tetrahydro-2H-pyran-3,4,5-triyl triacetate (7j)

(2R,3R,4S,5S,6S)-2-(Acetoxymethyl)-6-(4-(5-((4-oxo-4H-spiro[naphthalene-1,2'-oxazolidin]-3'-yl)sulfonyl)thiophen-2-yl)-1H-1,2,3-triazol-1-yl)tetrahydro-2H-pyran-3,4,5-triyl triacetate (7k)

(2R,3R,4S,5S,6S)-2-(Acetoxymethyl)-6-(4-(5-((8-oxo-1-oxa-4-azaspiro[4.5]deca-6,9-dien-4-yl)sulfonyl)pyridin-2-yl)-1H-1,2,3-triazol-1-yl)tetrahydro-2H-pyran-3,4,5-triyl triacetate (7l)

(2R,3R,4S,5R,6R)-2-(Acetoxymethyl)-6-(4-(5-((8-oxo-1-oxa-4-azaspiro[4.5]deca-6,9-dien-4-yl)sulfonyl)pyridin-2-yl)-1H-1,2,3-triazol-1-yl)tetrahydro-2H-pyran-3,4,5-triyl triacetate (7m)

4-((5-(1-((2R,3R,4S,5S,6R)-3,4,5-Trihydroxy-6-(hydroxymethyl)tetrahydro-2*H*-pyran-2-yl)-1*H*-1,2,3-triazol-4-yl)thiophen-2-yl)sulfonyl)-1-oxa-4-azaspiro[4.5]deca-6,9-dien-8-one (8a)

7-Chloro-4-((5-(1-((2R,3R,4S,5S,6R)-3,4,5-trihydroxy-6-(hydroxymethyl)tetrahydro-2*H*-pyran-2-yl)-1*H*-1,2,3-triazol-4-yl)thiophen-2-yl)sulfonyl)-1-oxa-4-azaspiro[4.5]deca-6,9-dien-8-one (8b)

3'-((5-(1-((2R,3R,4S,5S,6R)-3,4,5-Trihydroxy-6-(hydroxymethyl)tetrahydro-2*H*-pyran-2-yl)-1*H*-1,2,3-triazol-4-yl)thiophen-2-yl)sulfonyl)-4*H*-spiro[naphthalene-1,2'-oxazolidin]-4-one (8c)

4-((5-(1-((2R,3S,4S,5S,6R)-3,4,5-Trihydroxy-6-(hydroxymethyl)tetrahydro-2*H*-pyran-2-yl)-1*H*-1,2,3-triazol-4-yl)thiophen-2-yl)sulfonyl)-1-oxa-4-azaspiro[4.5]deca-6,9-dien-8-one (8d)

7-Chloro-4-((5-(1-((2R,3S,4S,5S,6R)-3,4,5-trihydroxy-6-(hydroxymethyl)tetrahydro-2*H*-pyran-2-yl)-1*H*-1,2,3-triazol-4-yl)thiophen-2-yl)sulfonyl)-1-oxa-4-azaspiro[4.5]deca-6,9-dien-8-one (8e)

3'-((5-(1-((2R,3S,4S,5S,6R)-3,4,5-Trihydroxy-6-(hydroxymethyl)tetrahydro-2H-pyran-2-yl)-1*H*-1,2,3-triazol-4-yl)thiophen-2-yl)sulfonyl)-4*H*-spiro[naphthalene-1,2'-oxazolidin]-4-one (8f)

4-((6-(1-((2R,3R,4S,5S,6R)-3,4,5-Trihydroxy-6-(hydroxymethyl)tetrahydro-2*H*-pyran-2-yl)-1*H*-1,2,3-triazol-4-yl)pyridin-3-yl)sulfonyl)-1-oxa-4-azaspiro[4.5]deca-6,9-dien-8-one (8g)

4-((6-(1-((2R,3S,4S,5S,6R)-3,4,5-Trihydroxy-6-(hydroxymethyl)tetrahydro-2*H*-pyran-2-yl)-1*H*-1,2,3-triazol-4-yl)pyridin-3-yl)sulfonyl)-1-oxa-4-azaspiro[4.5]deca-6,9-dien-8-one (8h)

N-(2-Phenoxyethyl)morpholine-4-sulfonamide (1a)

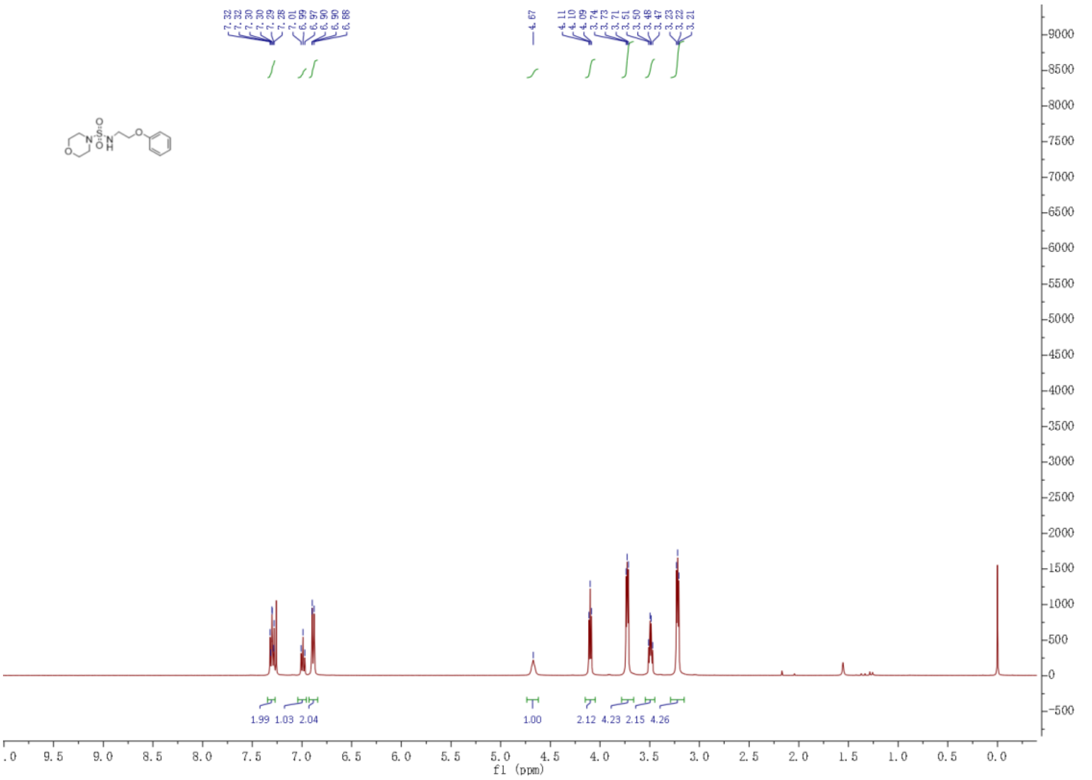

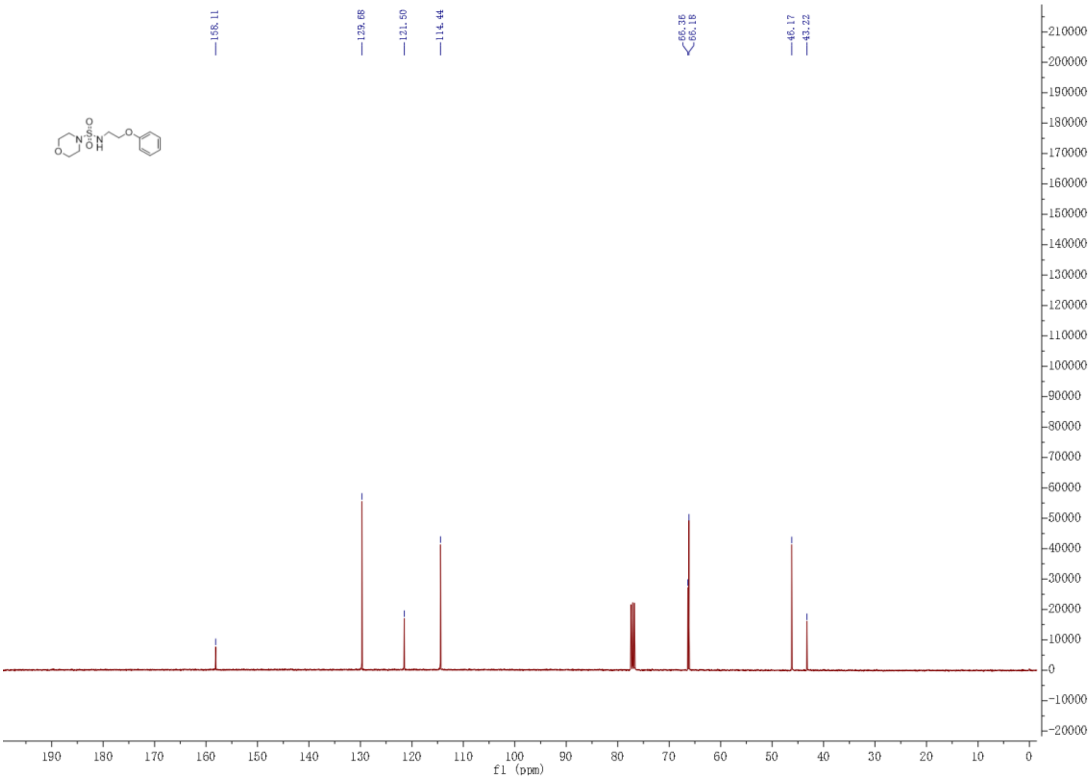

N-(2-(2-Chlorophenoxy)ethyl)morpholine-4-sulfonamide (1b)

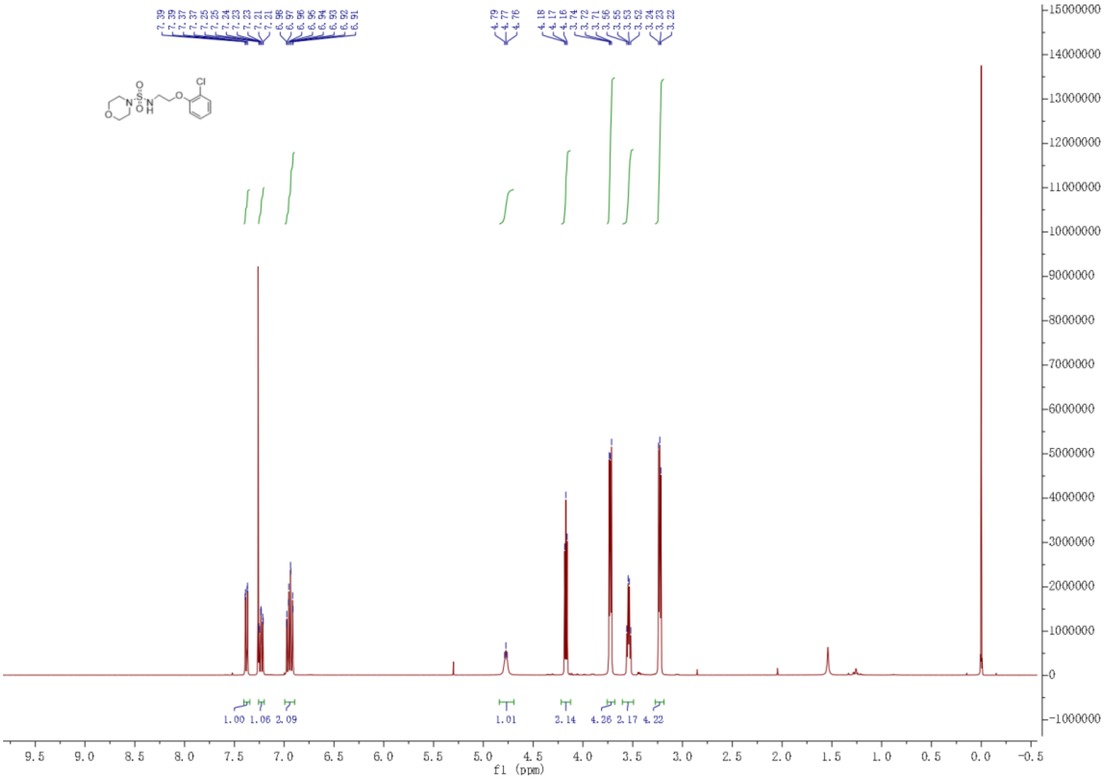

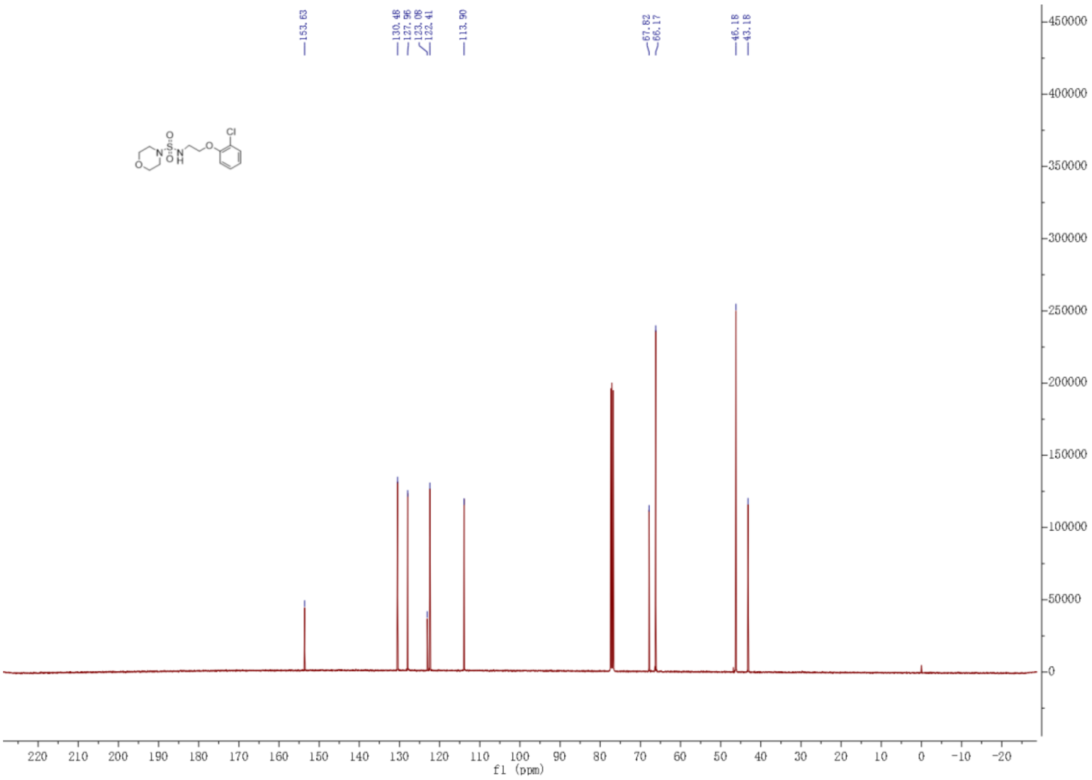

N-(2-(3-Chlorophenoxy)ethyl)morpholine-4-sulfonamide (1c)

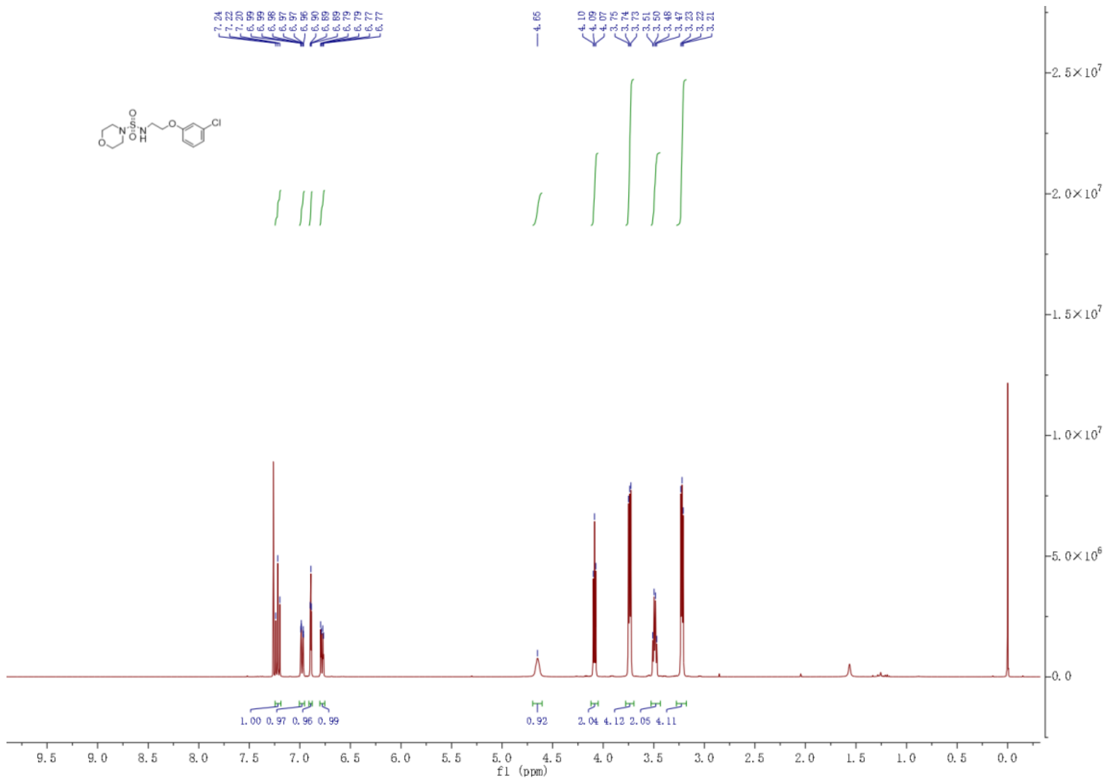

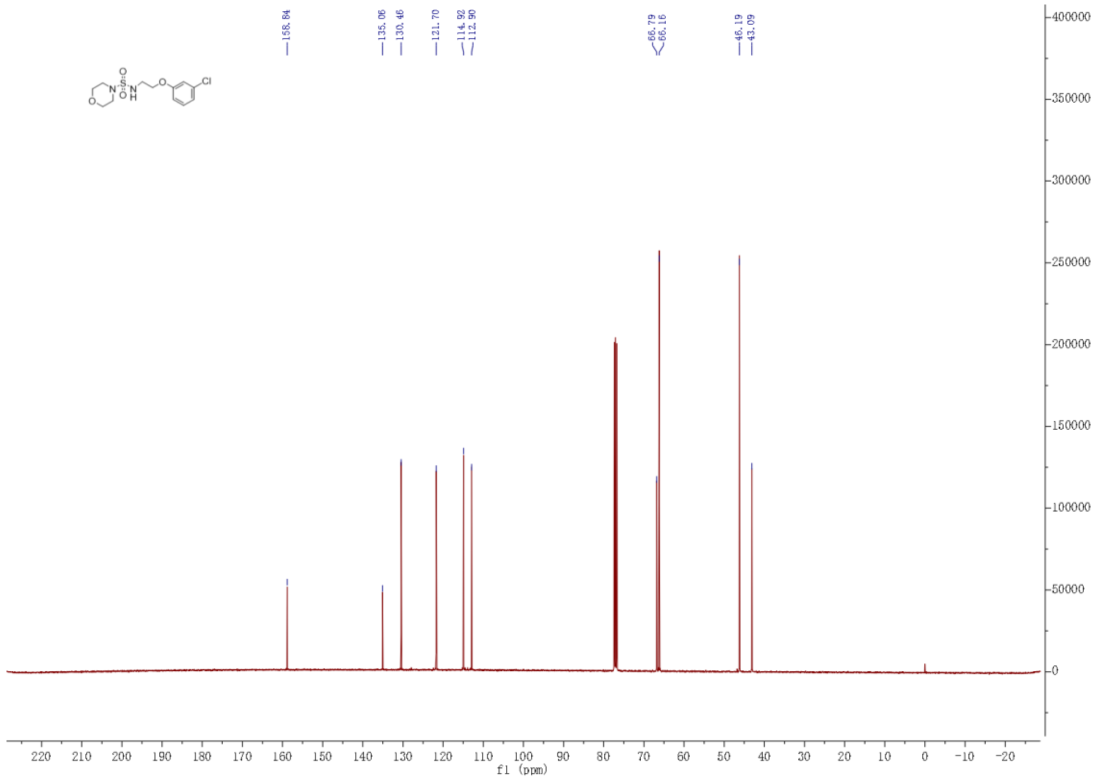

N-(2-(Naphthalen-1-yloxy)ethyl)morpholine-4-sulfonamide (1d)

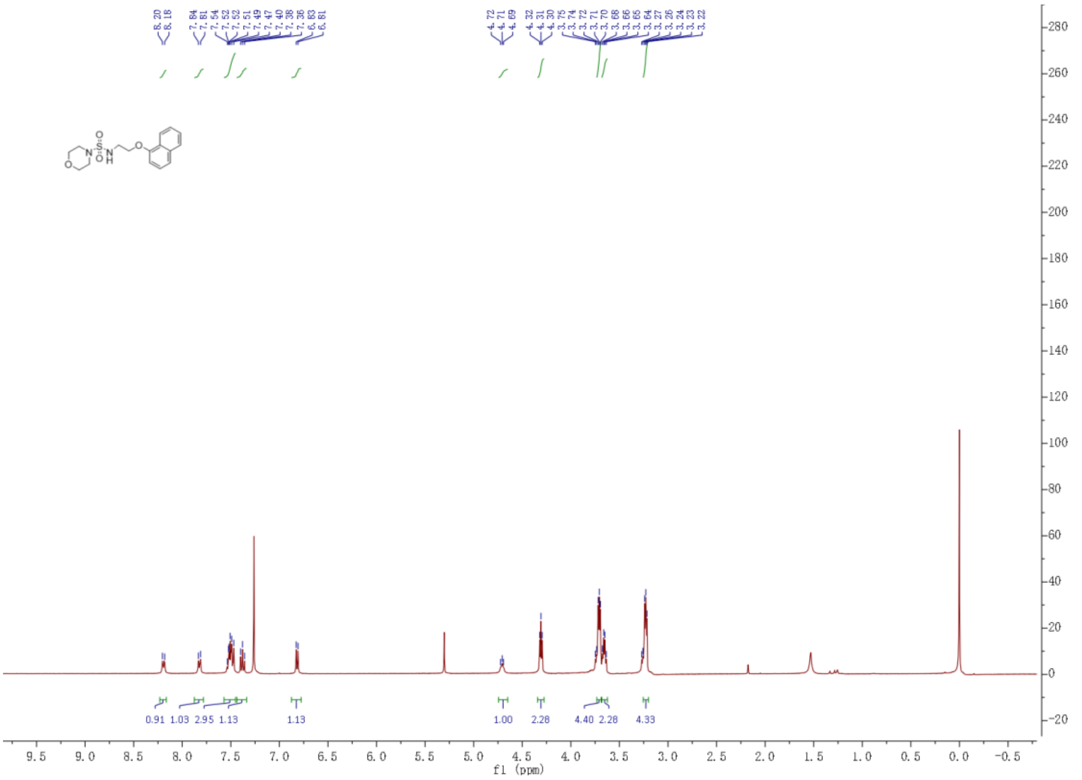

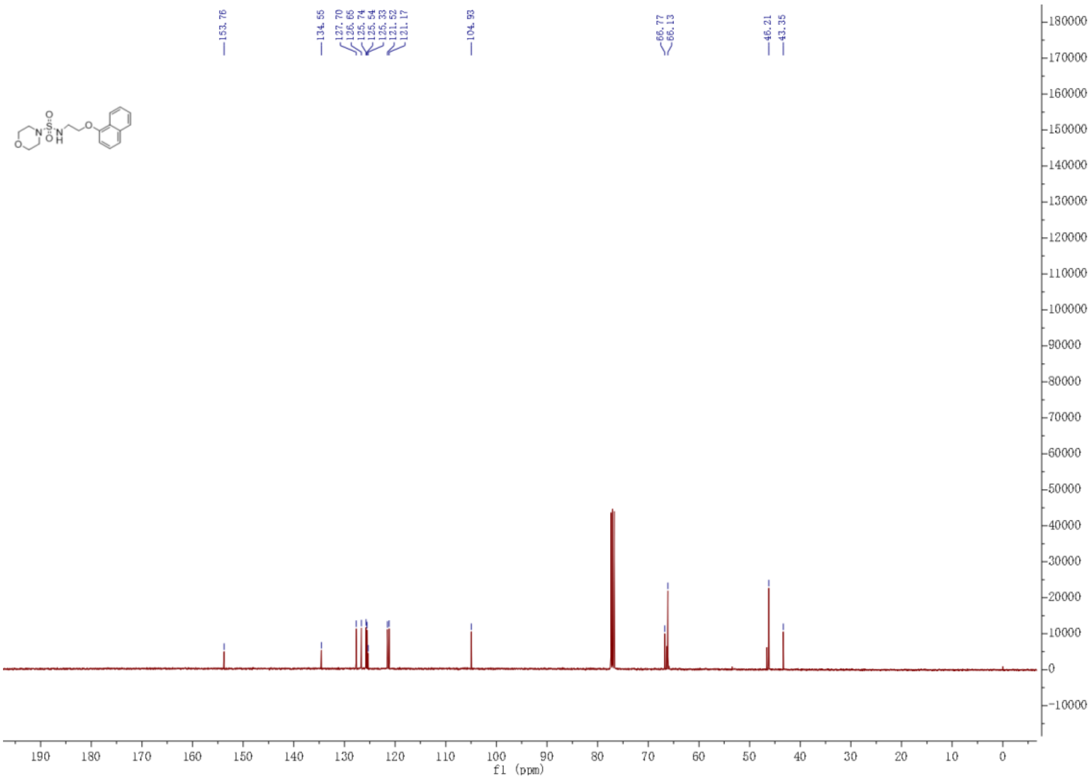

N-(2-Phenoxyethyl)thiophene-2-sulfonamide (1e)

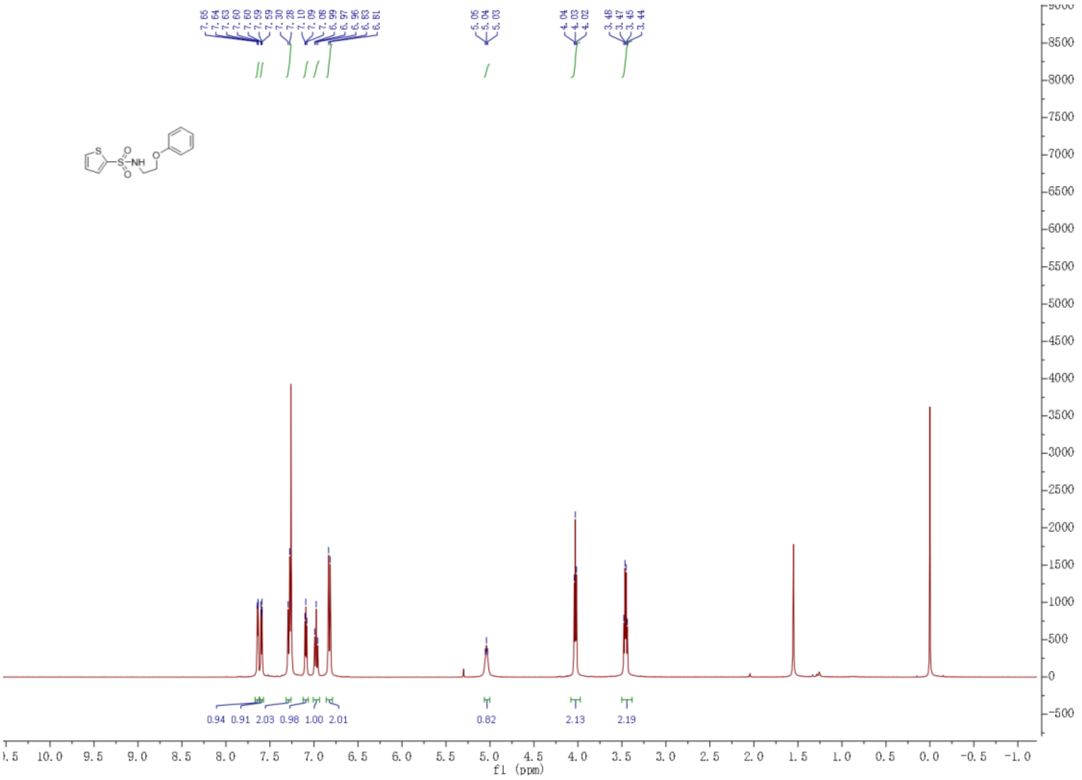

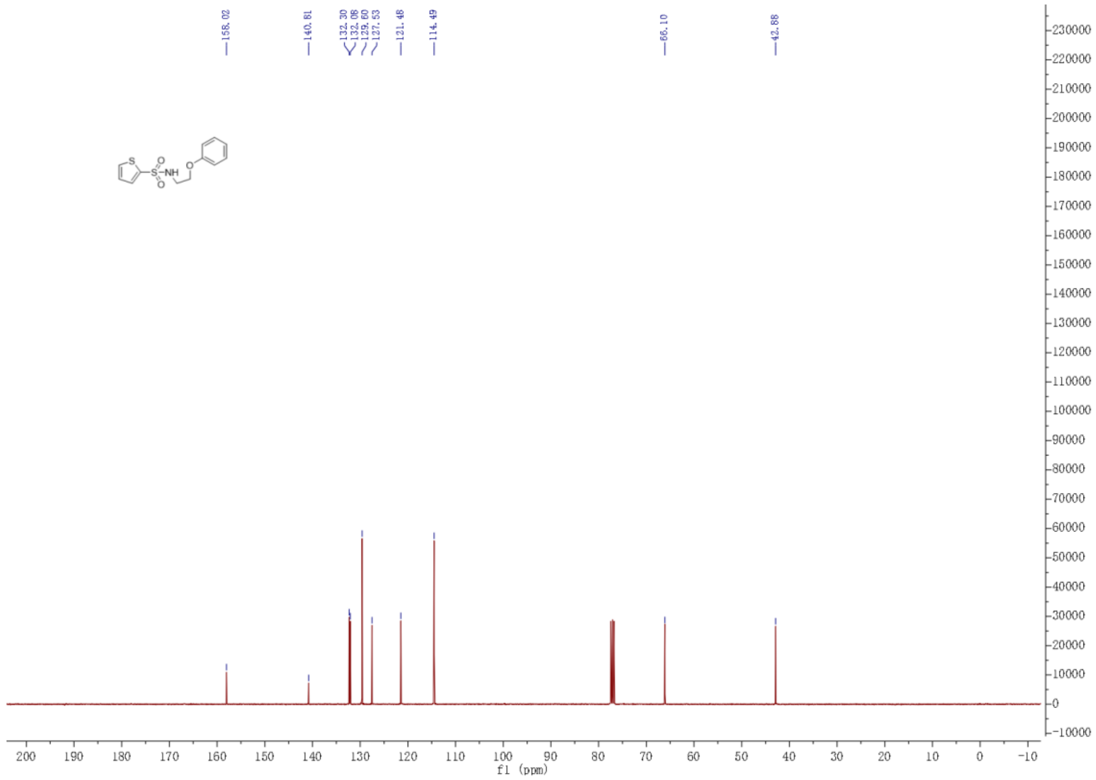

N-(2-(2-Bromophenoxy)ethyl)thiophene-2-sulfonamide (1f)

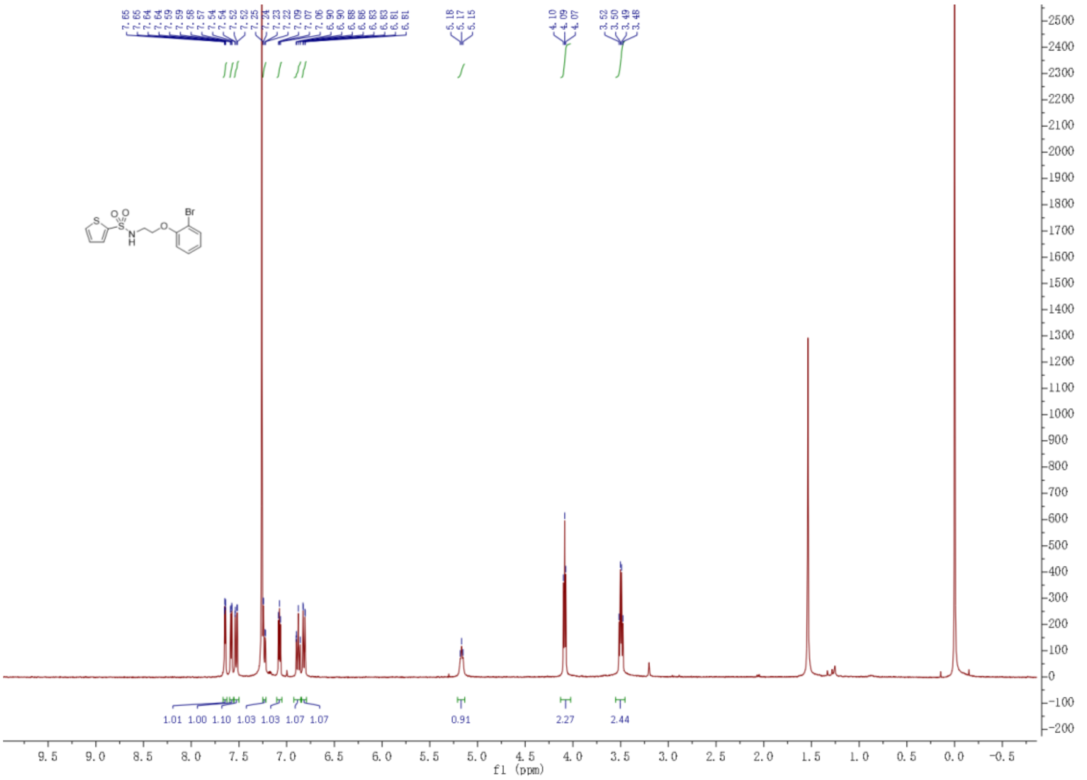
7.65 7.65 7.64 7.64 7.59 7.58 7.57 7.54 7.54 7.52 7.52 7.25 7.24 7.23 7.22 7.09 7.07 7.06 6.90 6.90 6.88 6.86 6.83 6.83 6.81 6.81
5.18 5.17 5.15
4.10 4.09 4.07
3.52 3.50 3.49 3.48
Br
S
O O
N
H
O
1.01 1.00 1.10 1.03 1.03 1.07 1.07
0.91
2.27
2.44
f1 (ppm)

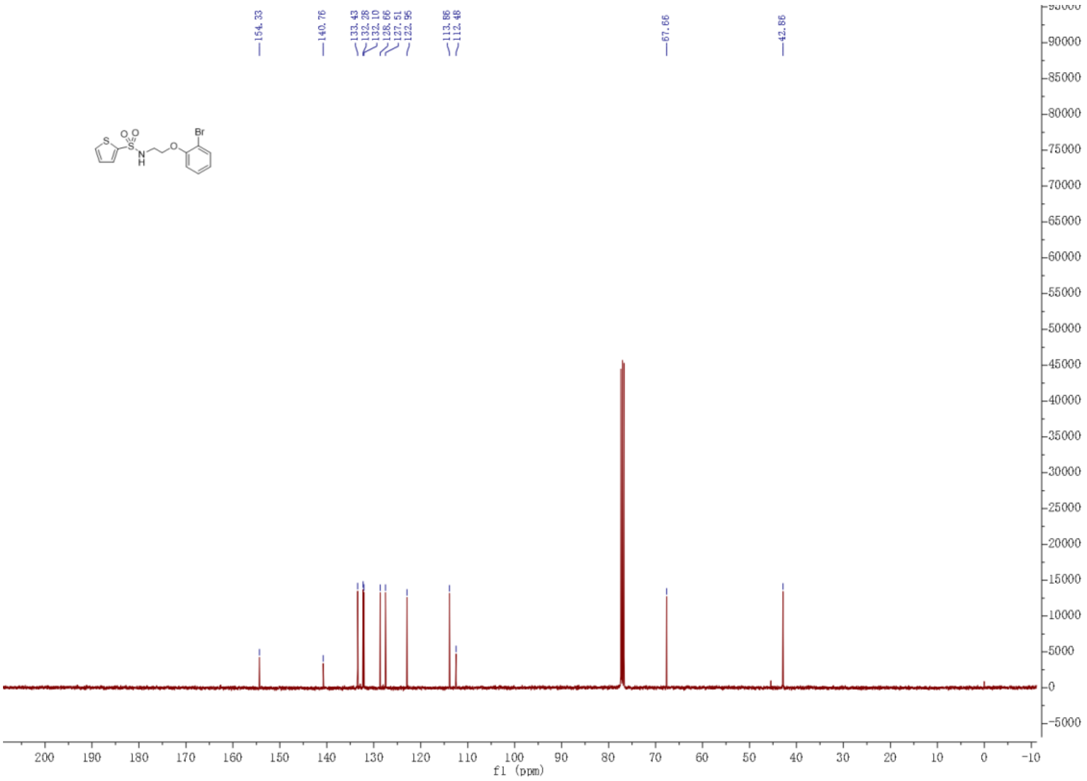
154.33
140.76
133.43 132.28 132.10 128.66 127.51 122.95
113.86 112.48
67.66
42.86
Br
S
O O
N
H
O
f1 (ppm)

N-(2-Phenoxyethyl)-6-chloropyridine-3-sulfonamide (1g)

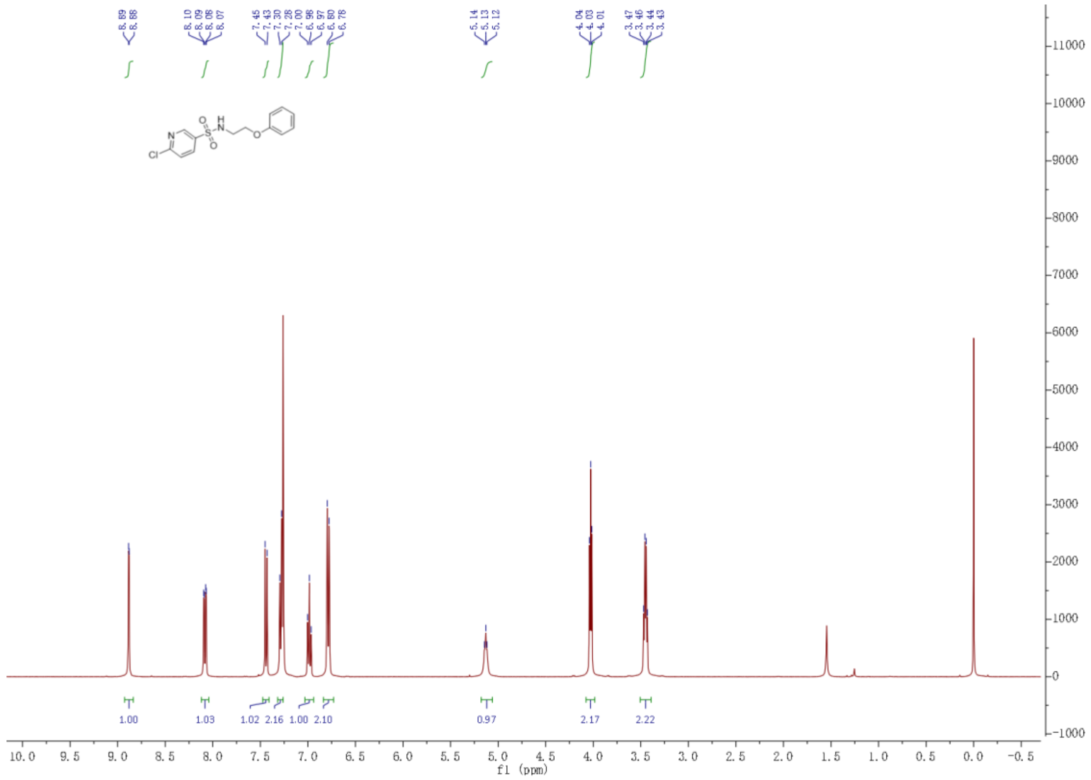

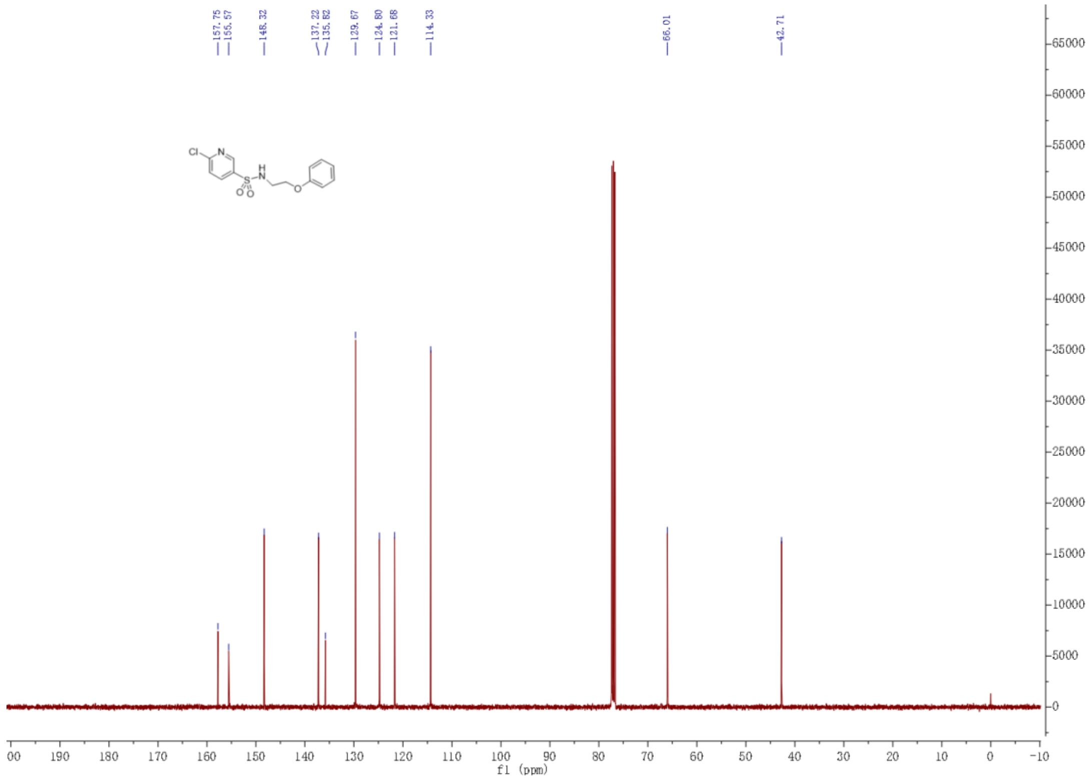

5-Ethynyl-N-(2-phenoxyethyl)thiophene-2-sulfonamide (5a)

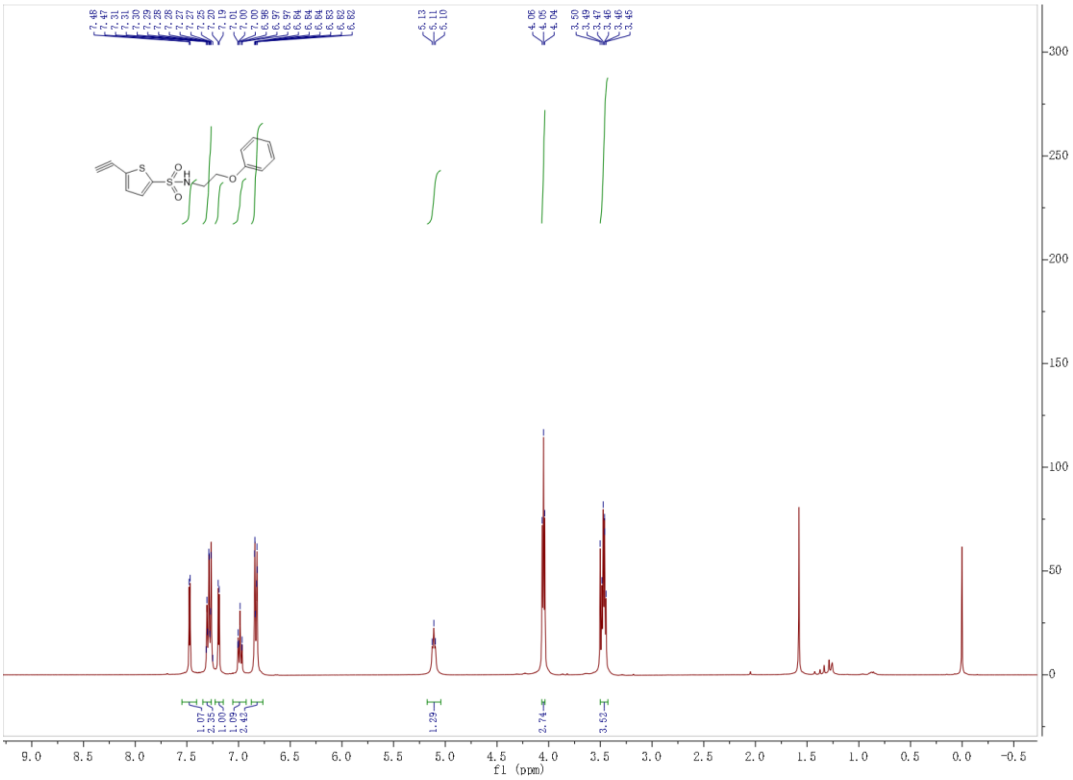

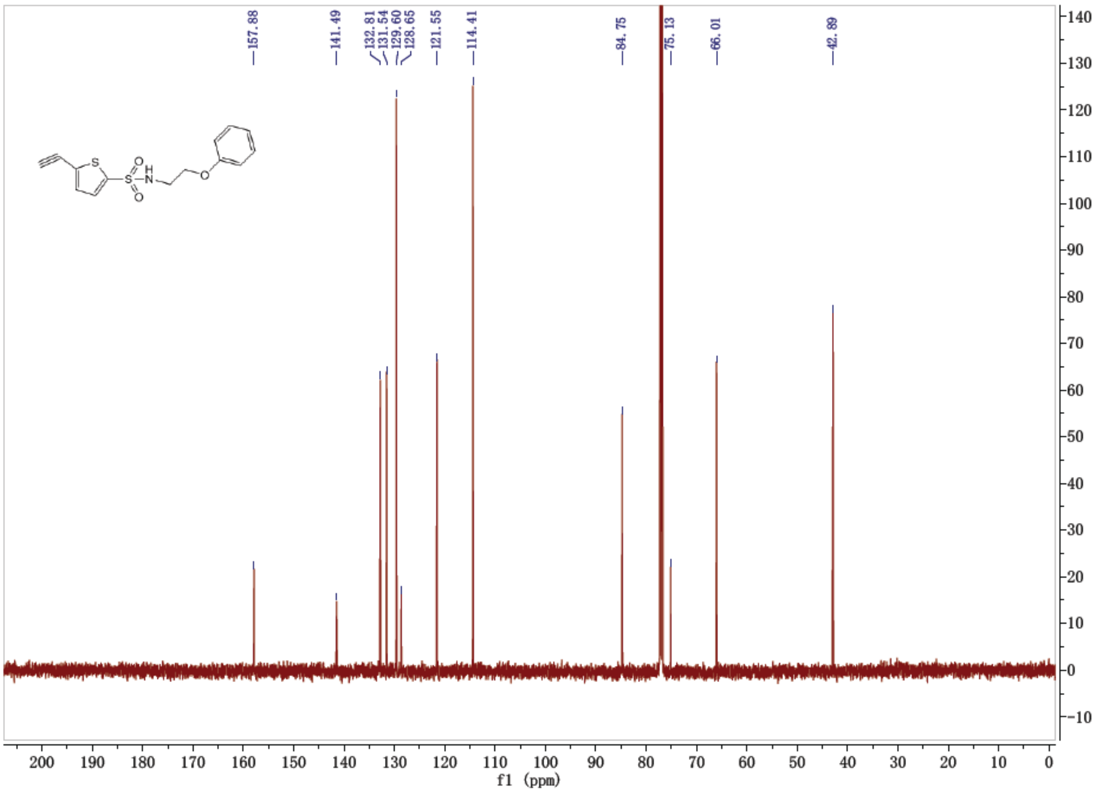

5-Ethynyl-N-(2-(2-fluorophenoxy)ethyl)thiophene-2-sulfonamide (5b)

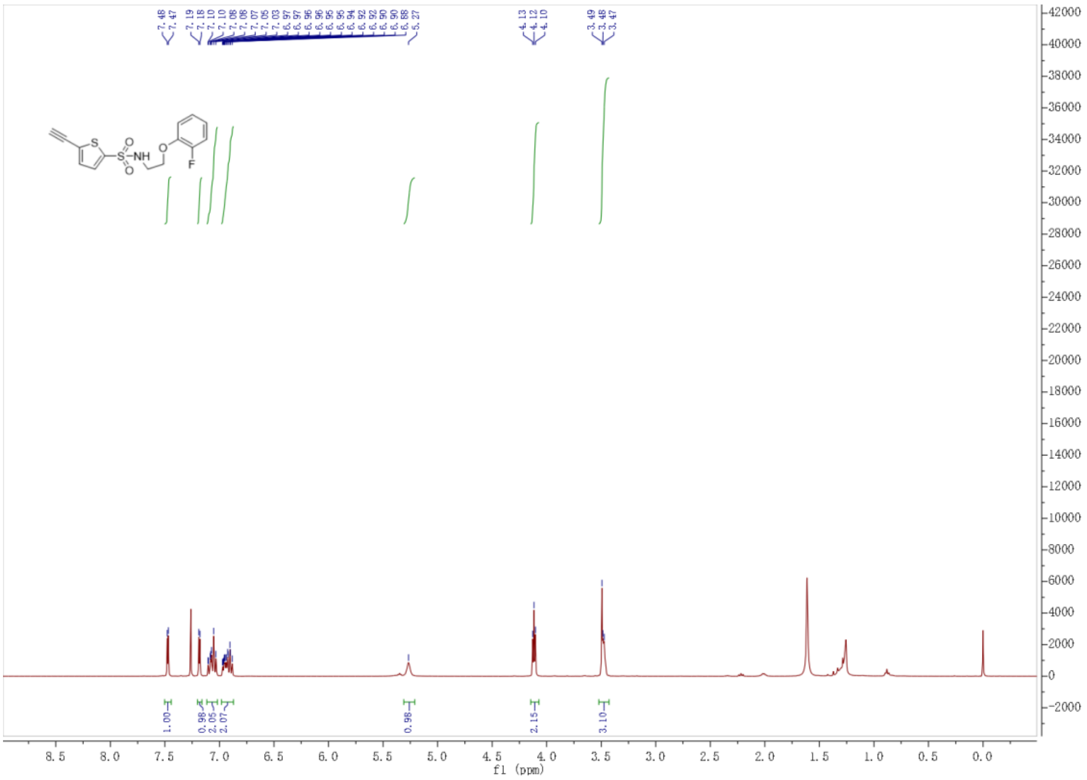

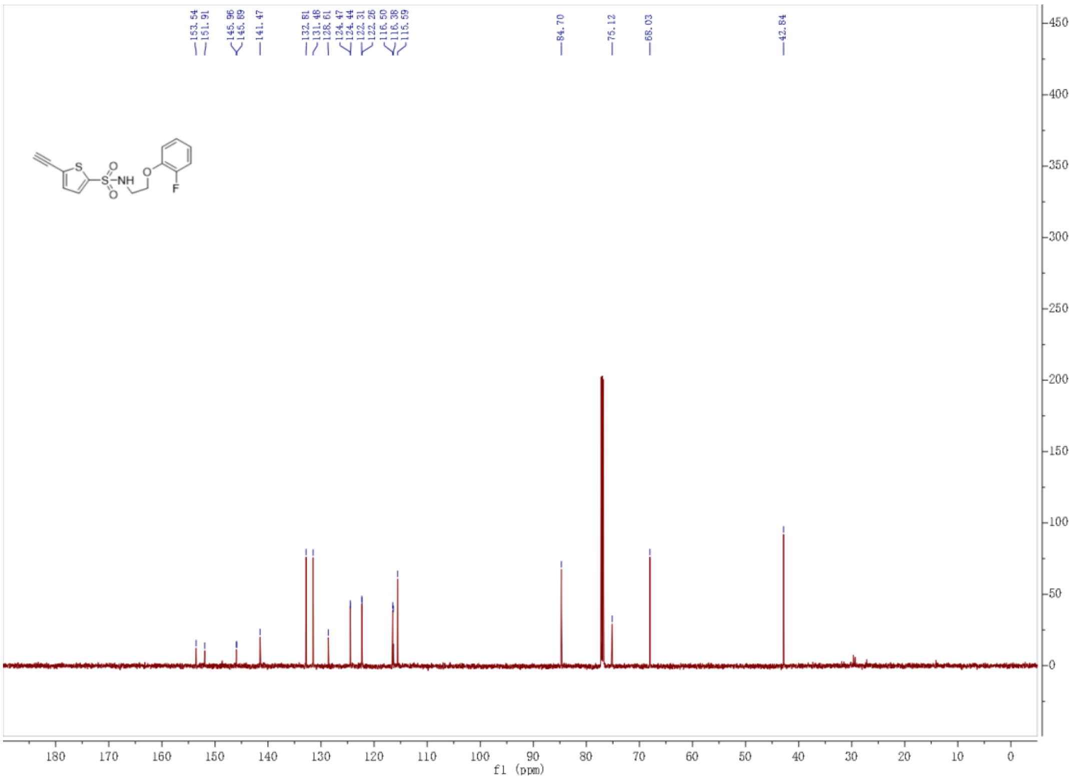

5-Ethynyl-N-(2-(o-tolyloxy)ethyl)thiophene-2-sulfonamide (5c)

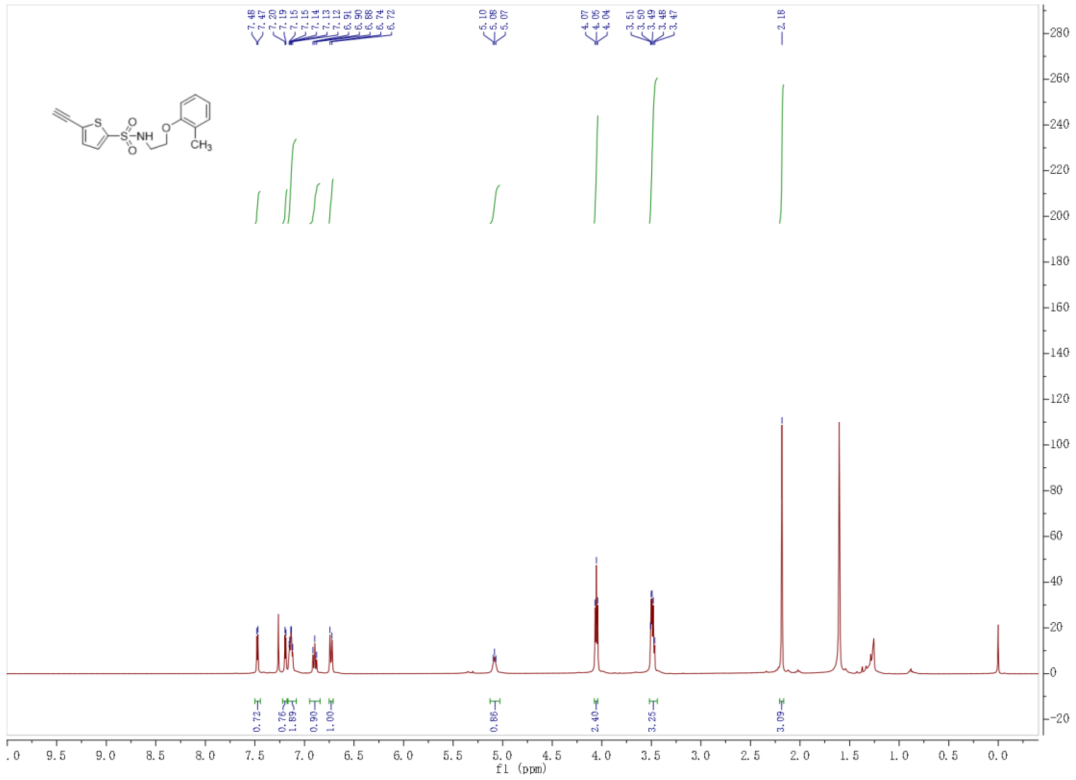

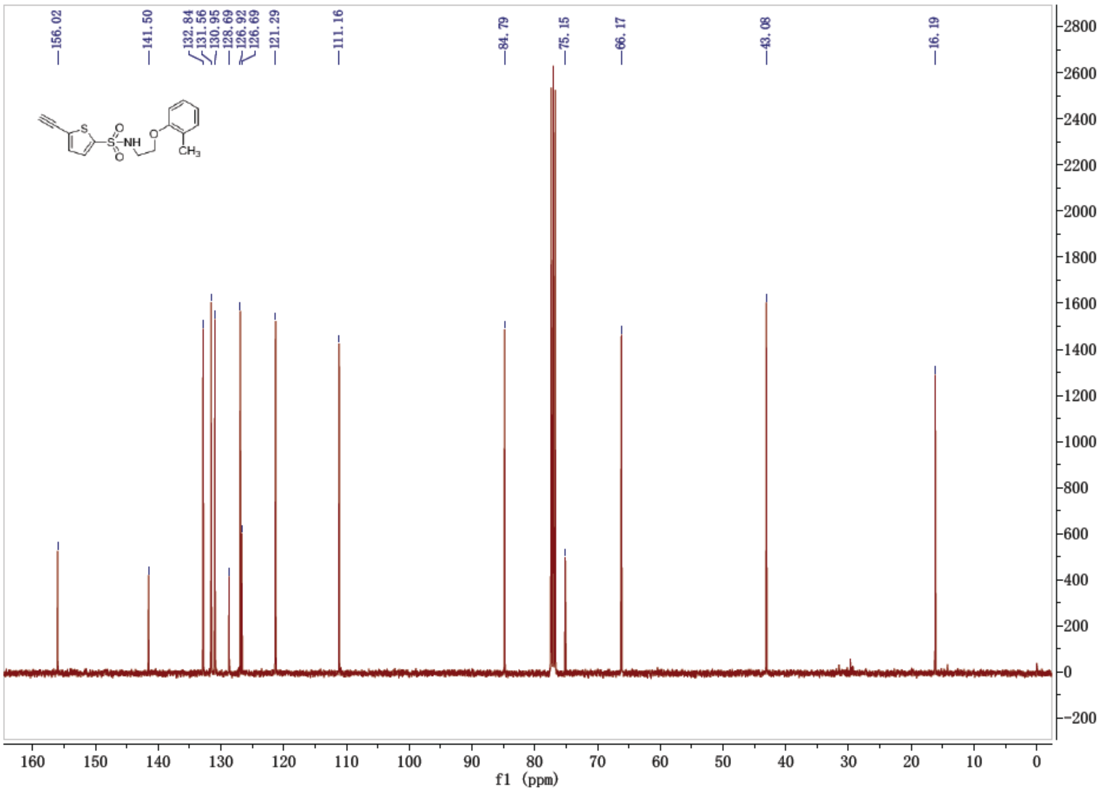

N-(2-(2-Bromophenoxy)ethyl)-5-ethynylthiophene-2-sulfonamide (5d)

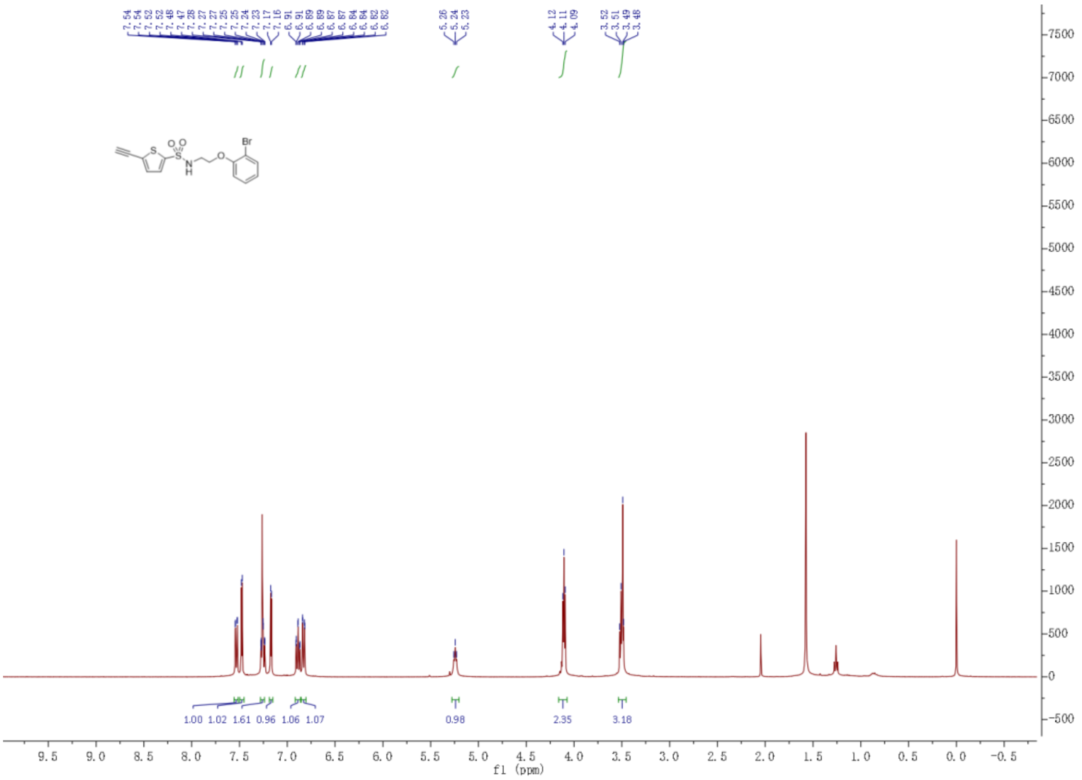

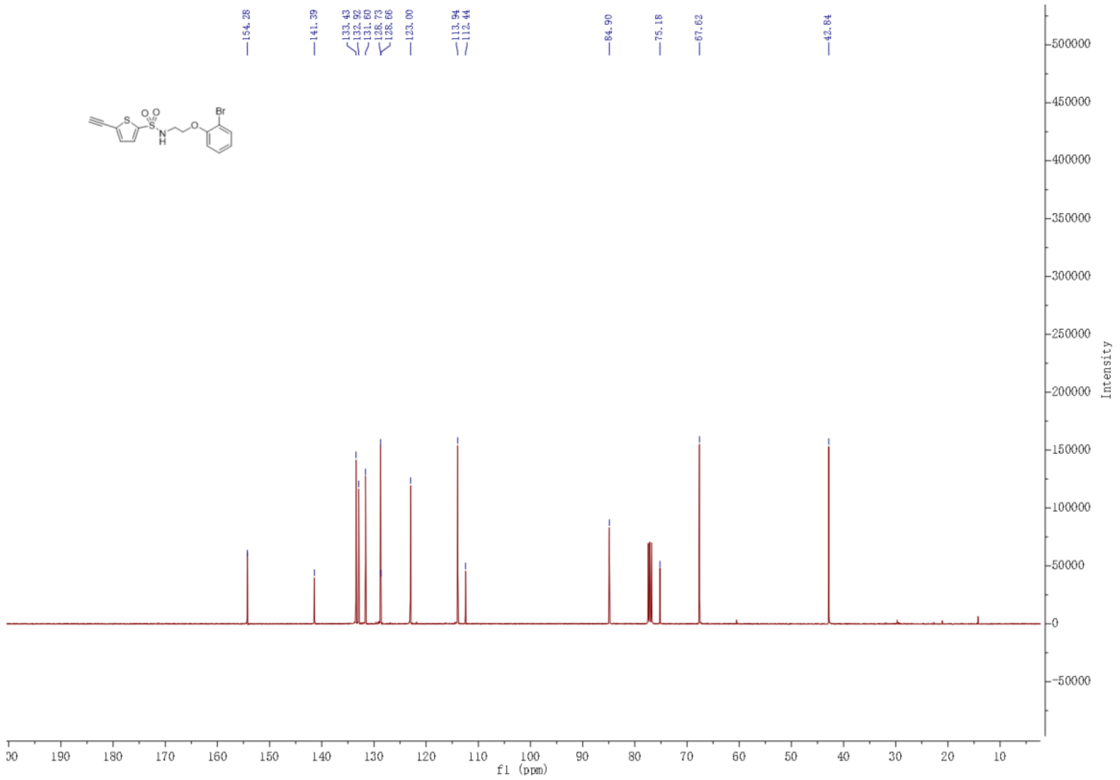

N-(2-(2-Chlorophenoxy)ethyl)-5-ethynylthiophene-2-sulfonamide (5e)

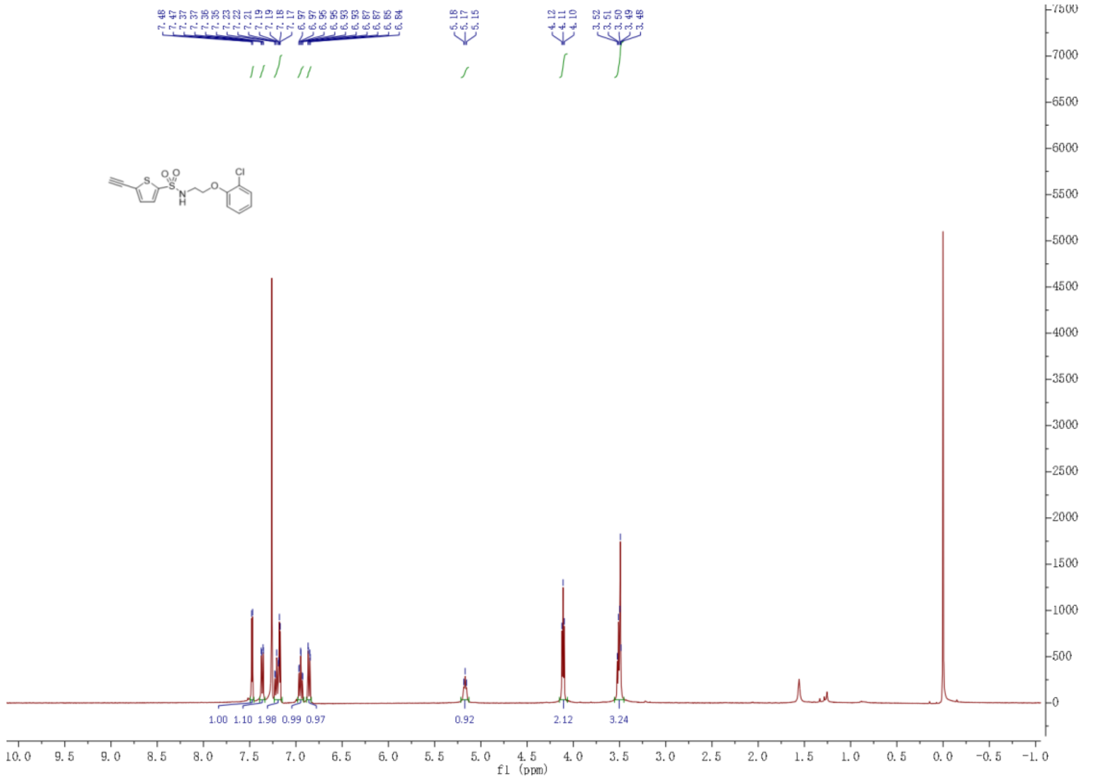

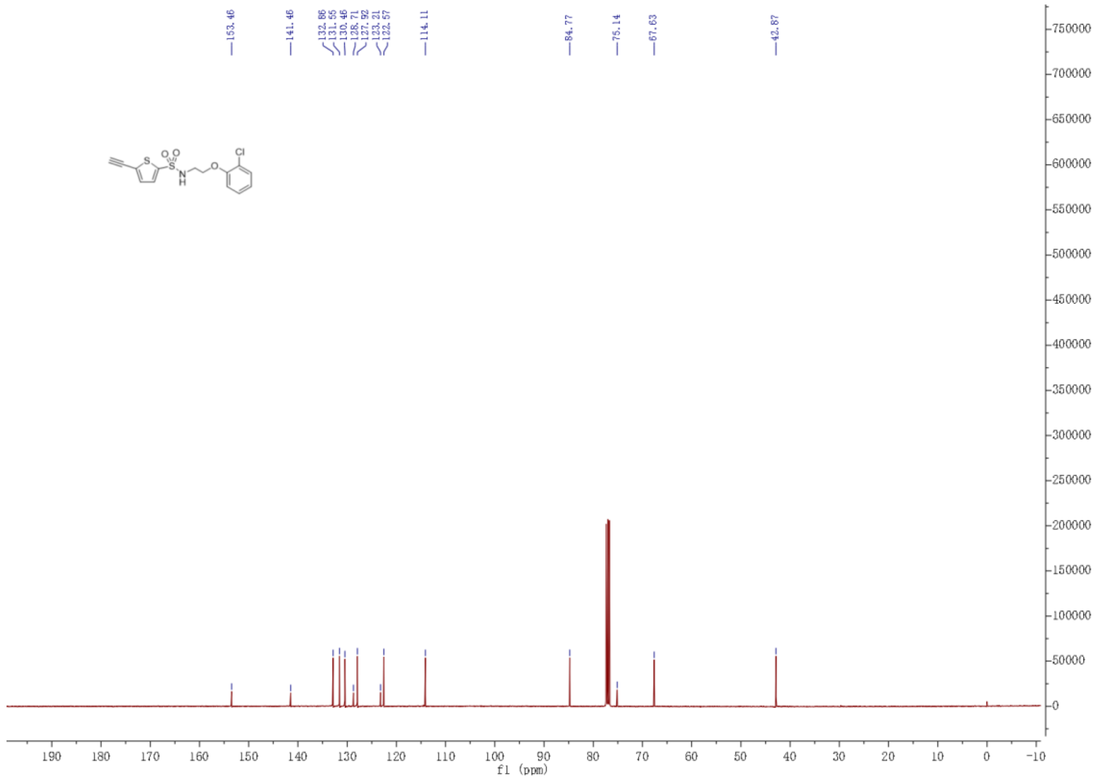

N-(2-(3-Chlorophenoxy)ethyl)-5-ethynylthiophene-2-sulfonamide (5f)

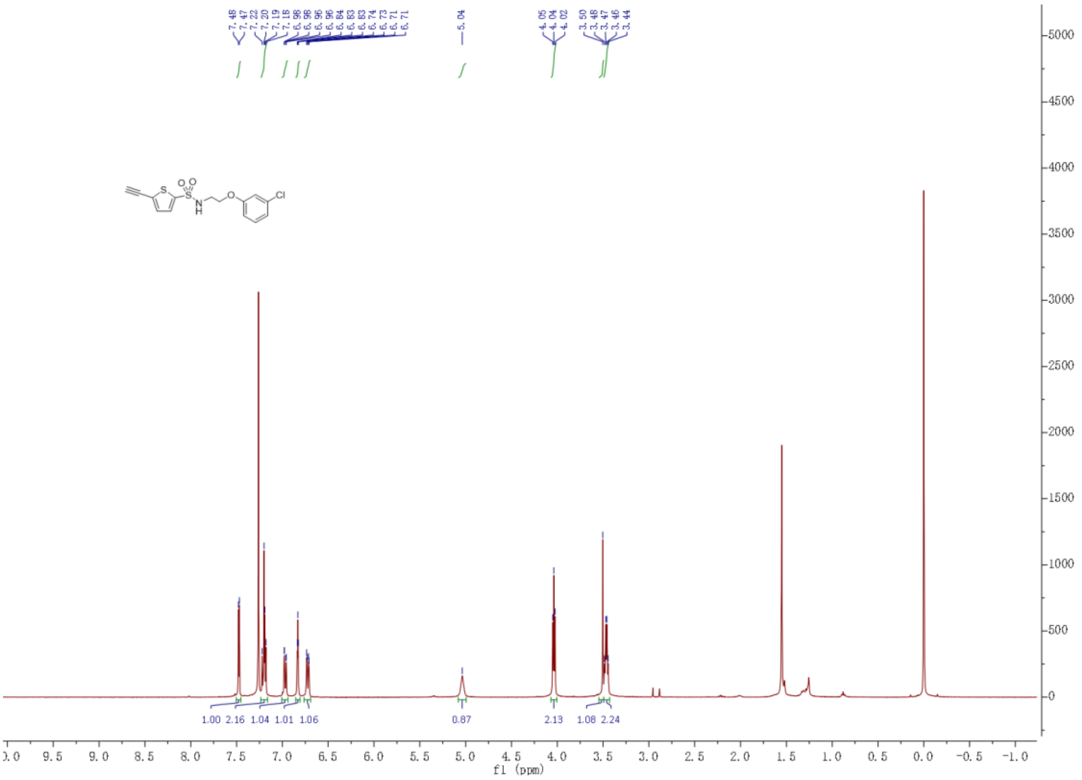

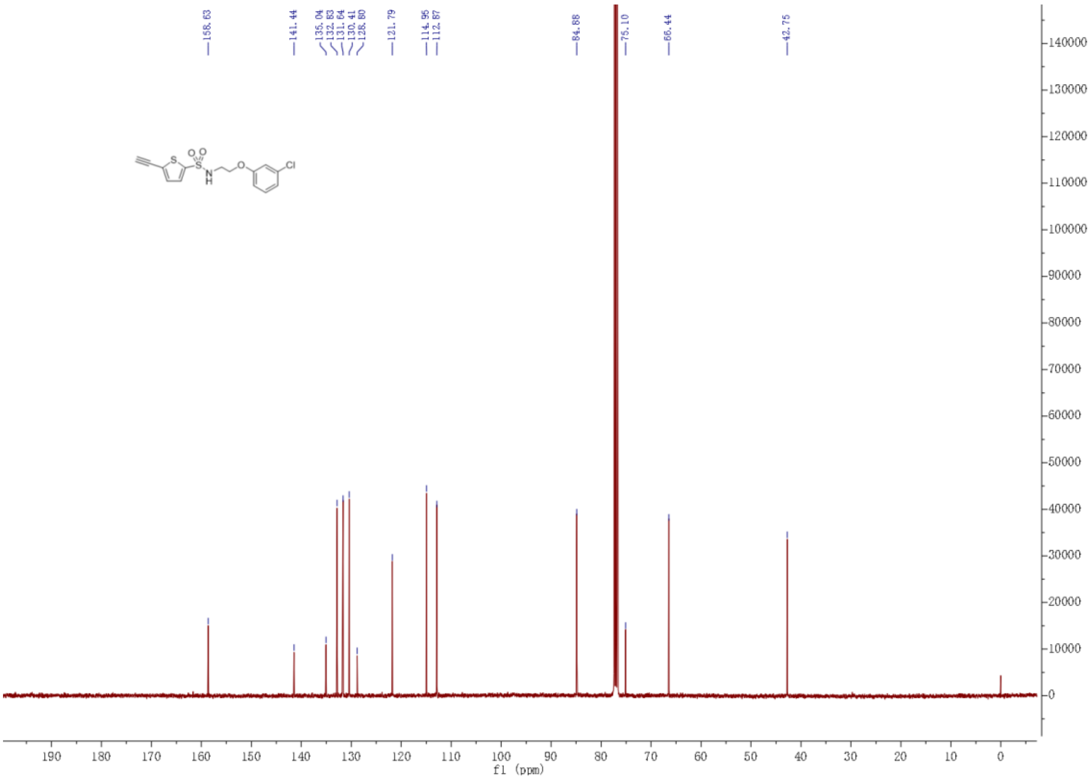

N-(2-(2,3-Dimethylphenoxy)ethyl)-5-ethynylthiophene-2-sulfonamide (5g)

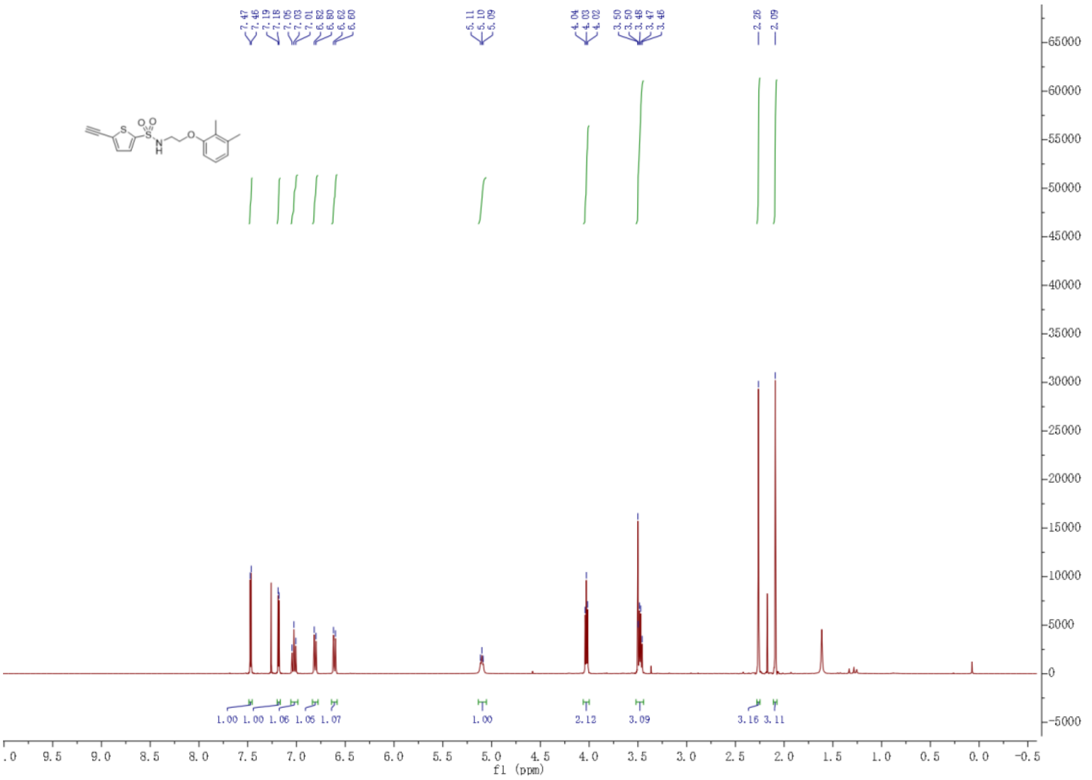

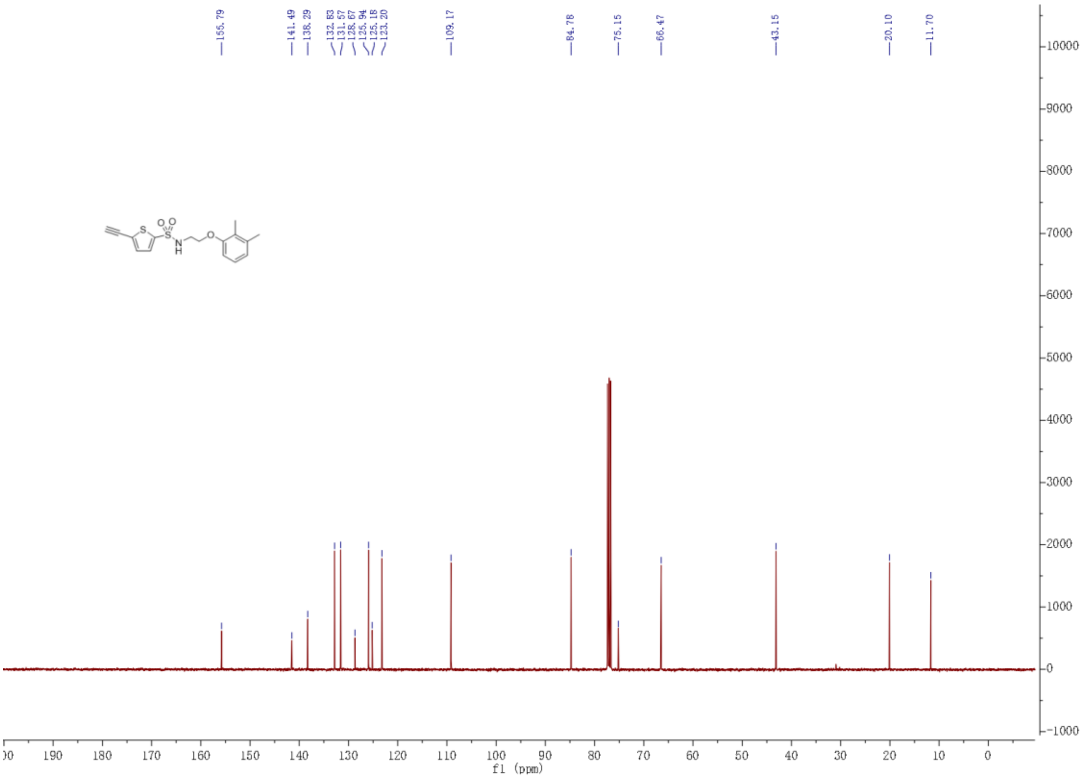

5-Ethynyl-N-(2-(naphthalen-1-yloxy)ethyl)thiophene-2-sulfonamide (5h)

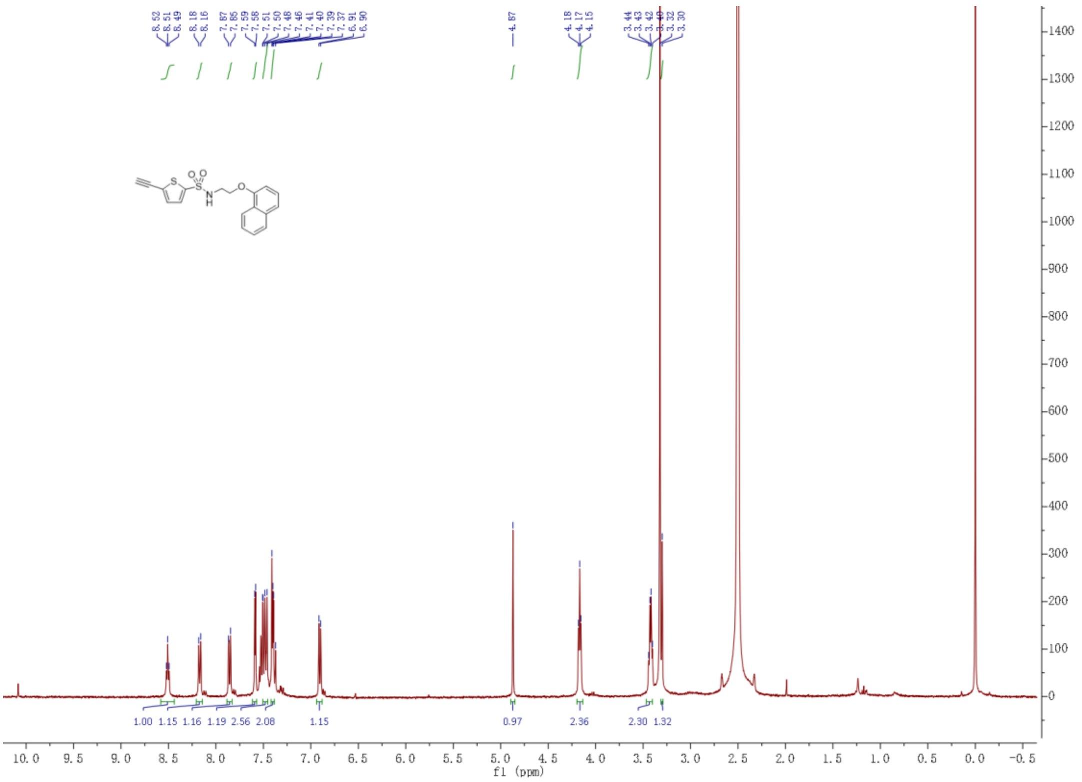

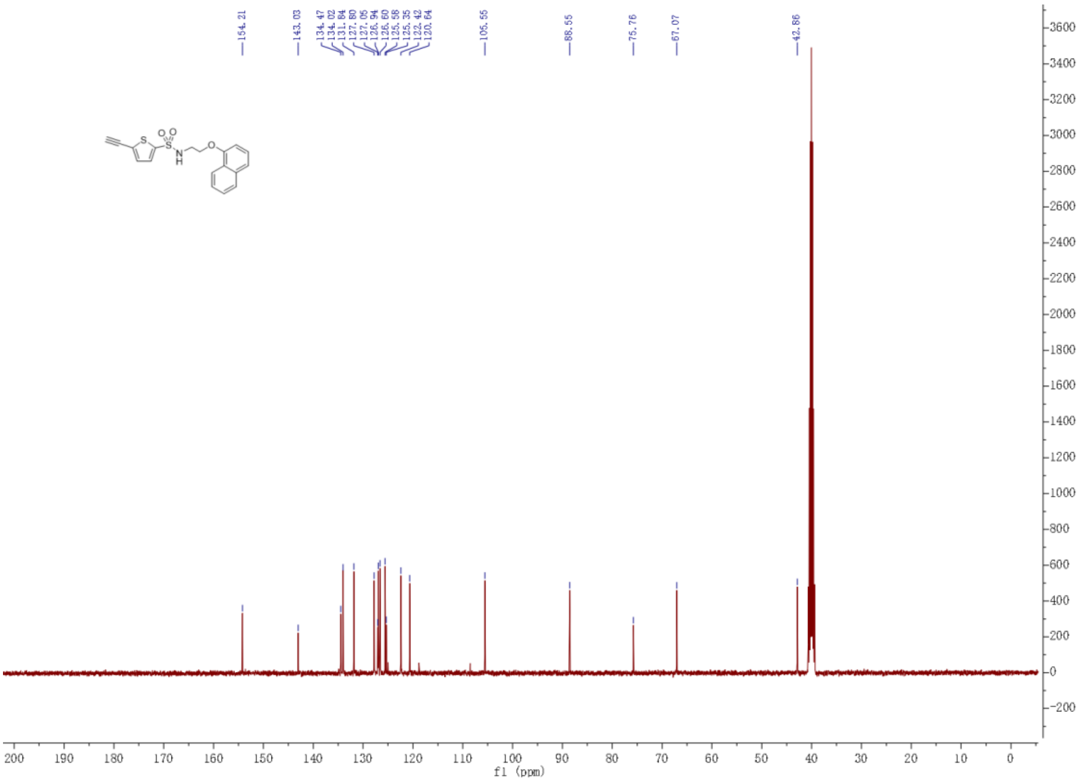

N-(2-Phenoxyethyl)-6-((trimethylsilyl)ethynyl)pyridine-3-sulfonamide (5i)

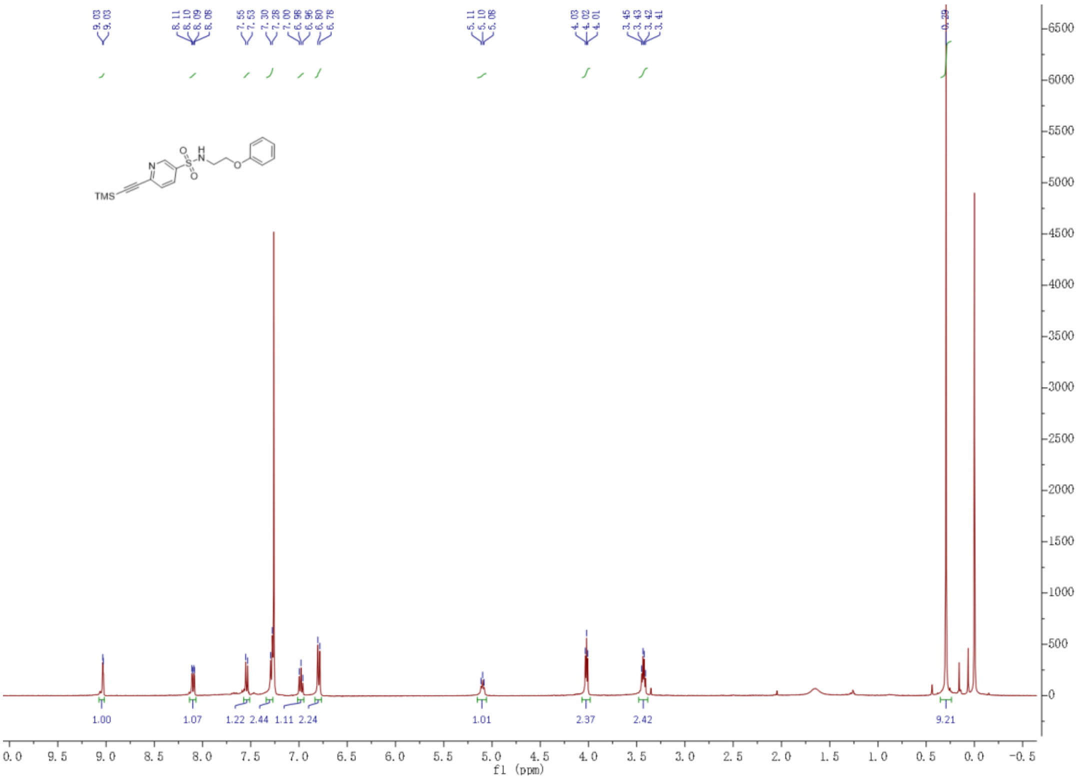

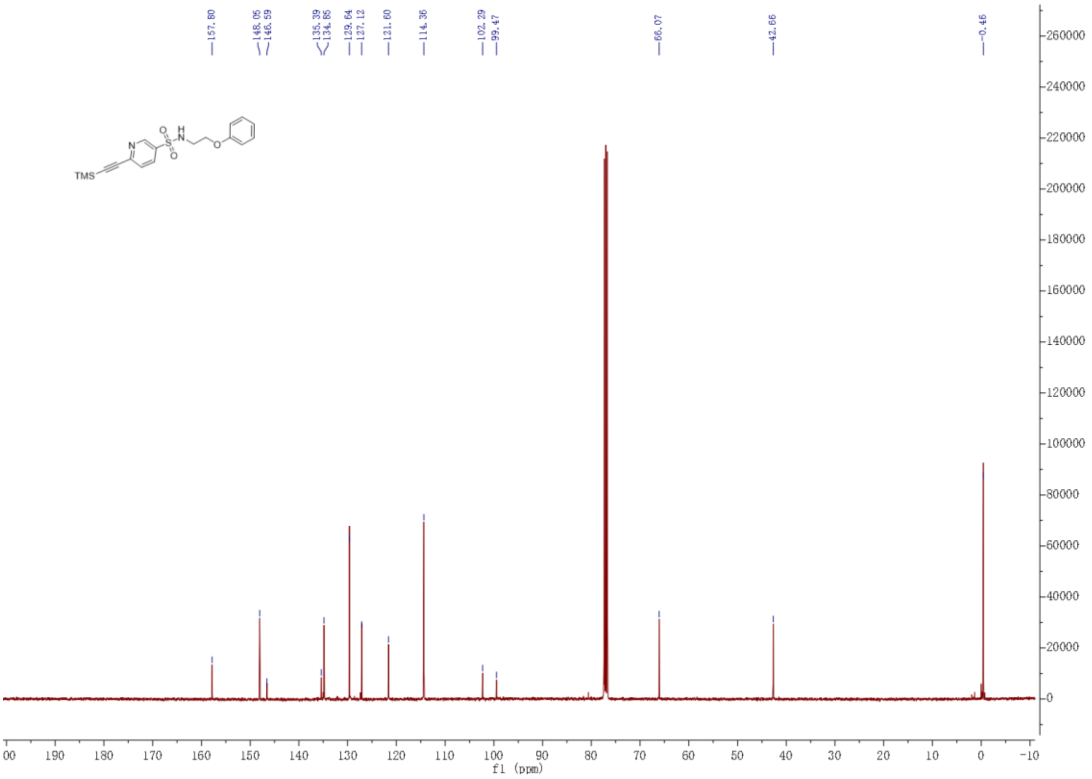

4-(Morpholinosulfonyl)-1-oxa-4-azaspiro[4.5]deca-6,9-dien-8-one (2a)

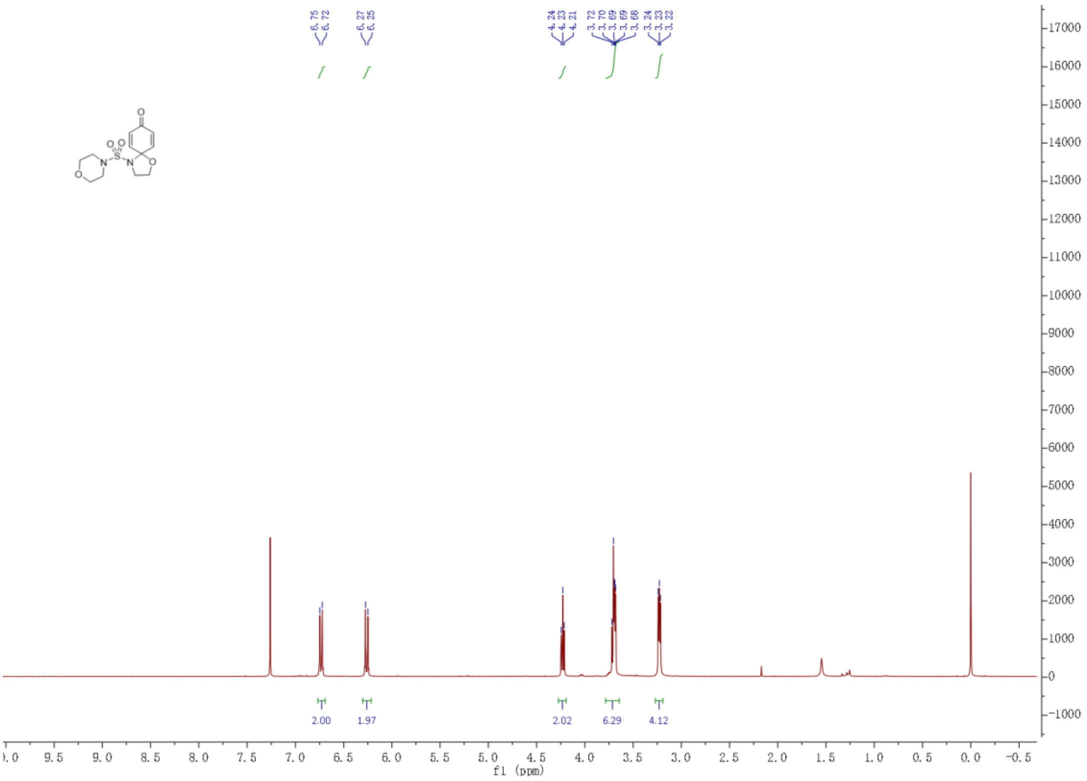

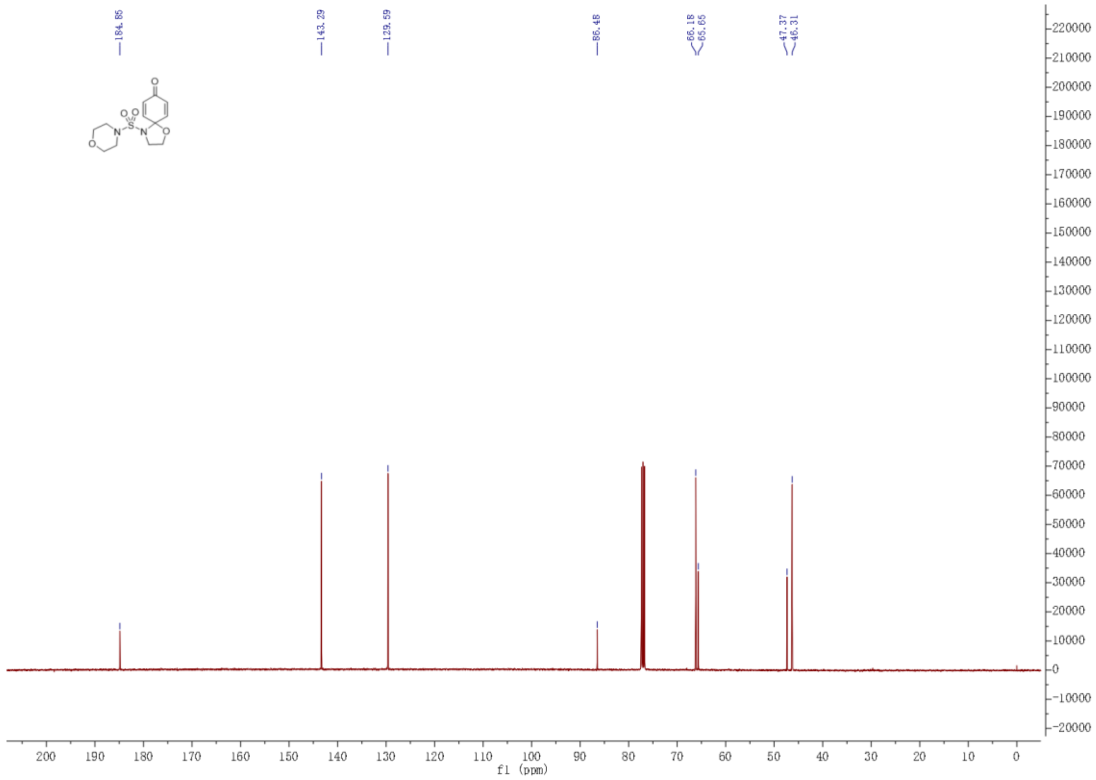

6-Chloro-4-(morpholinosulfonyl)-1-oxa-4-azaspiro[4.5]deca-6,9-dien-8-one (2b)

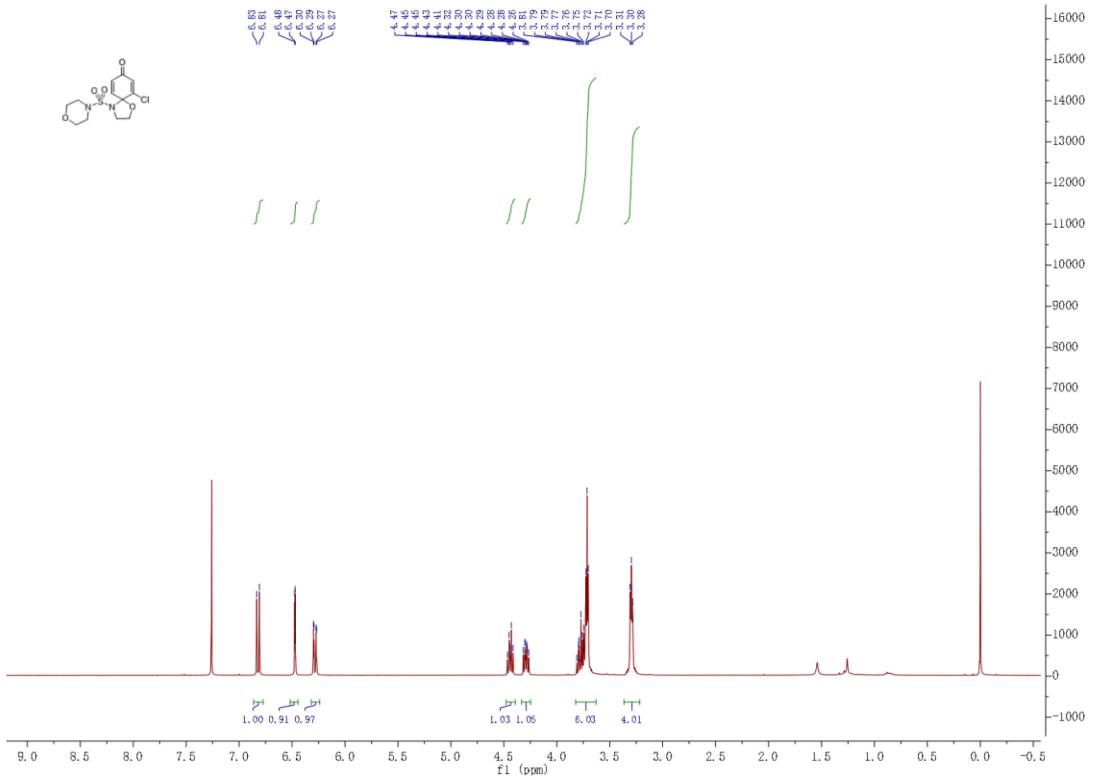
6.83
6.81
6.48
6.47
6.30
6.29
6.27
6.27
1.00
0.91
0.97
1.03
1.05
6.03
4.01
f1 (ppm)
Cl
O
N
S

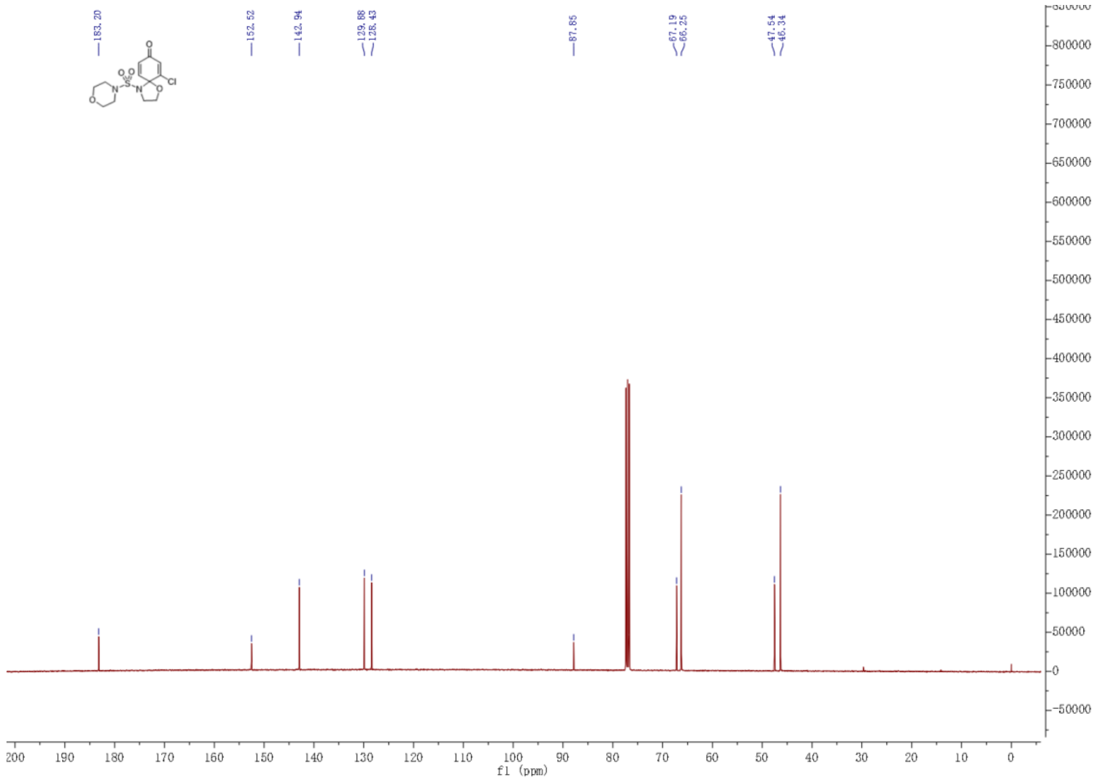
183.20
152.52
142.94
129.88
128.43
87.85
67.19
66.25
47.54
46.34
f1 (ppm)
Cl
O
N
S

7-Chloro-4-(morpholinosulfonyl)-1-oxa-4-azaspiro[4.5]deca-6,9-dien-8-one (2c)

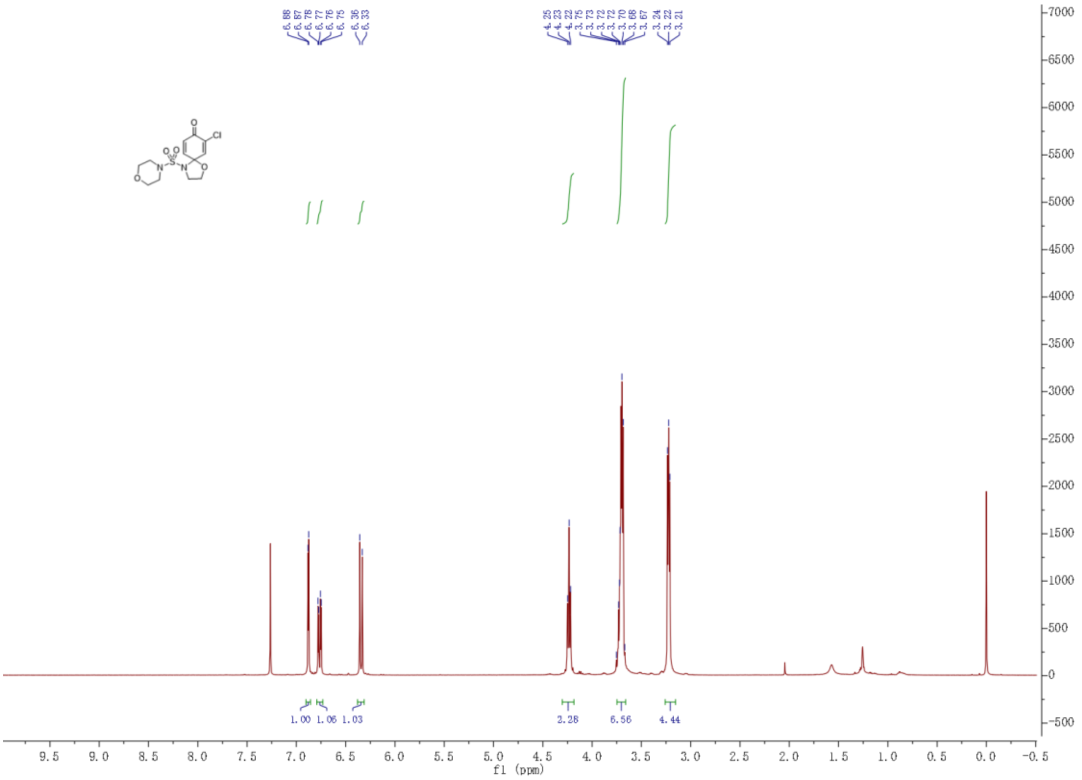

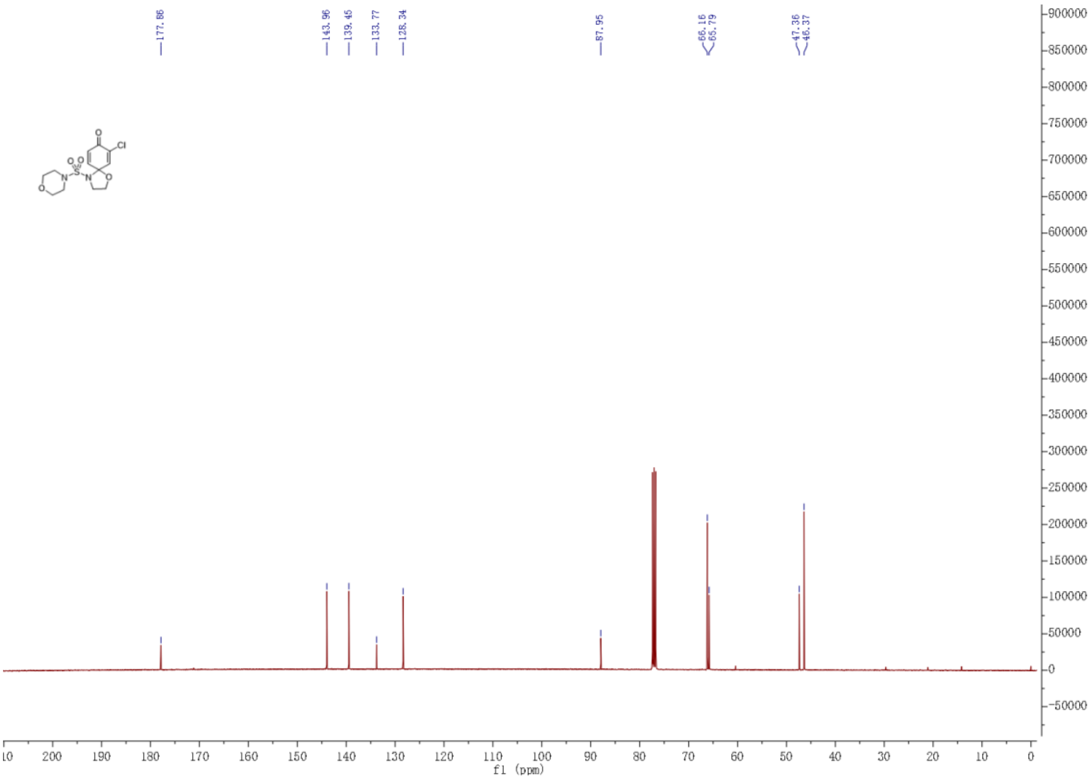

3'-(Morpholinosulfonyl)-4H-spiro[naphthalene-1,2'-oxazolidin]-4-one (2d)

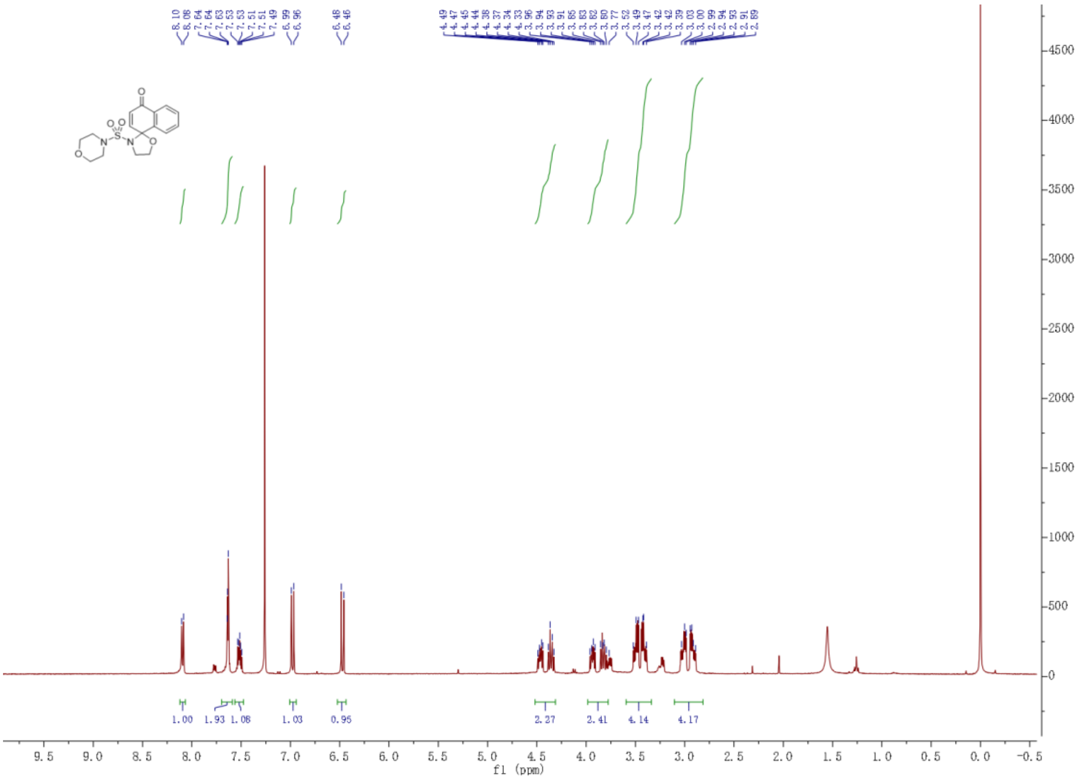

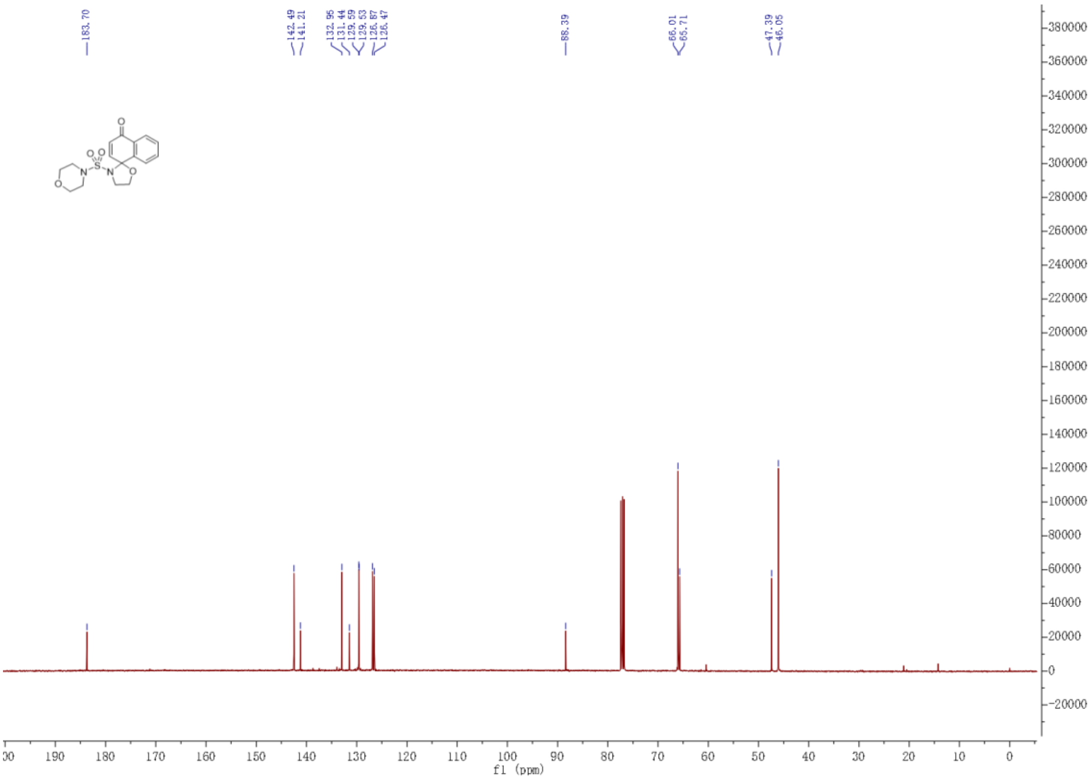

4-(Thiophen-2-ylsulfonyl)-1-oxa-4-azaspiro[4.5]deca-6,9-dien-8-one (2e)

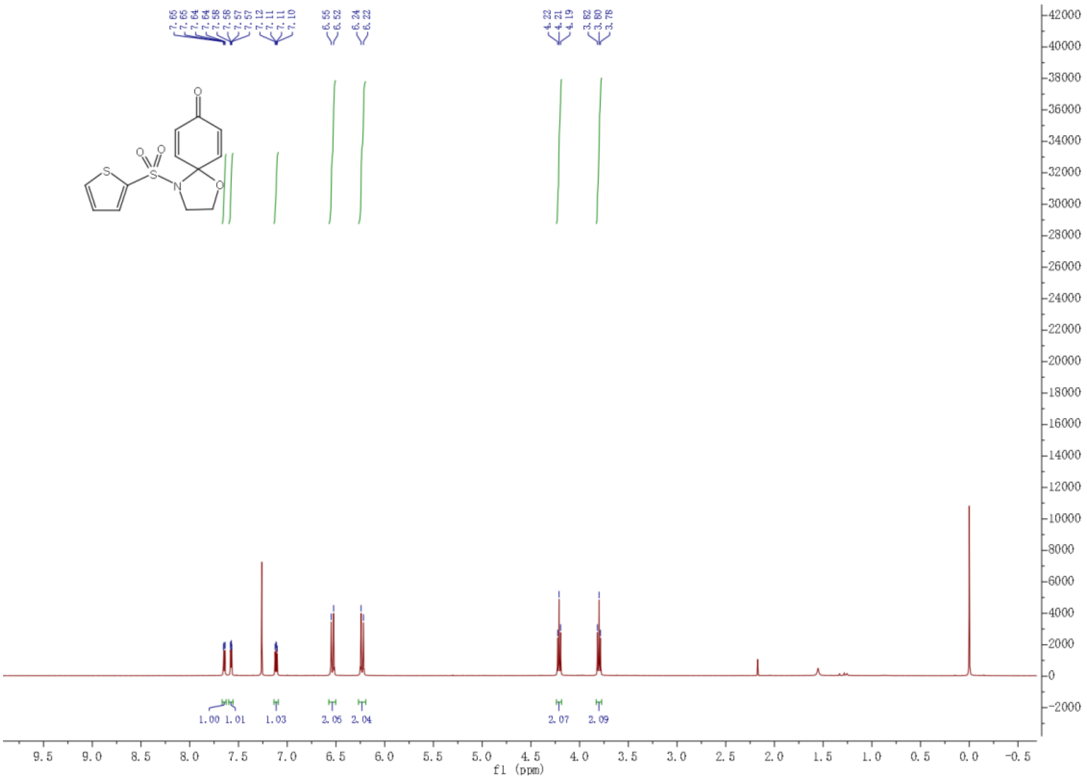

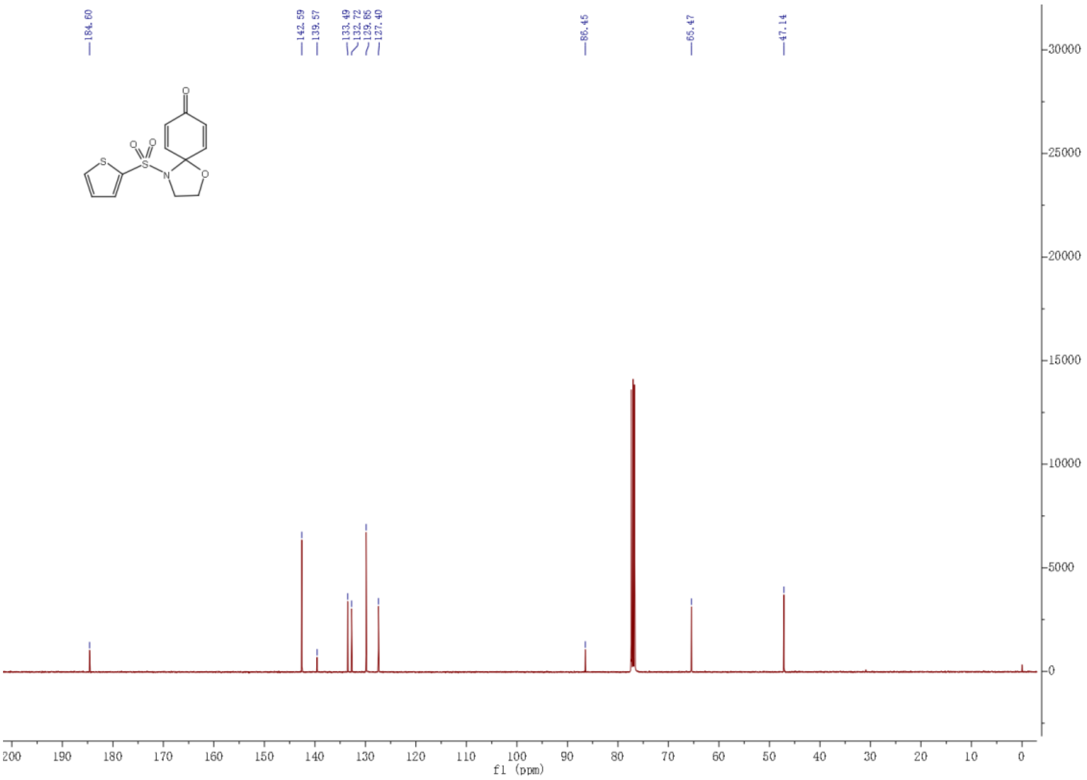

6-Bromo-4-(thiophen-2-ylsulfonyl)-1-oxa-4-azaspiro[4.5]deca-6,9-dien-8-one (2f)

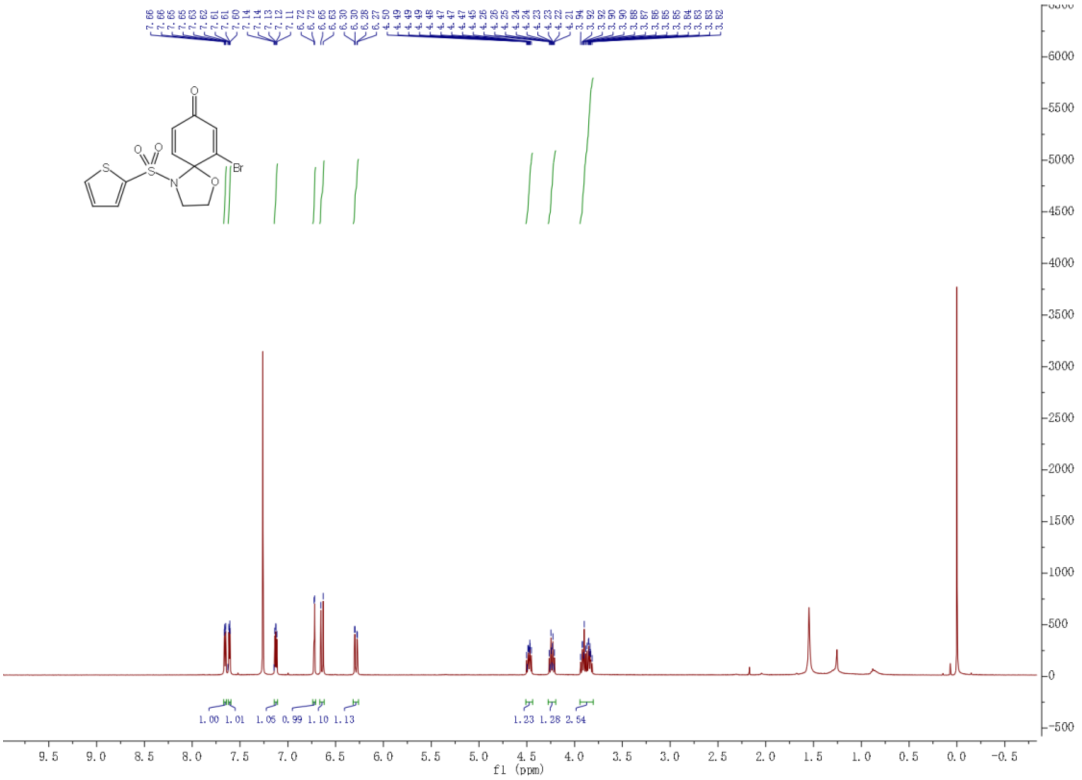

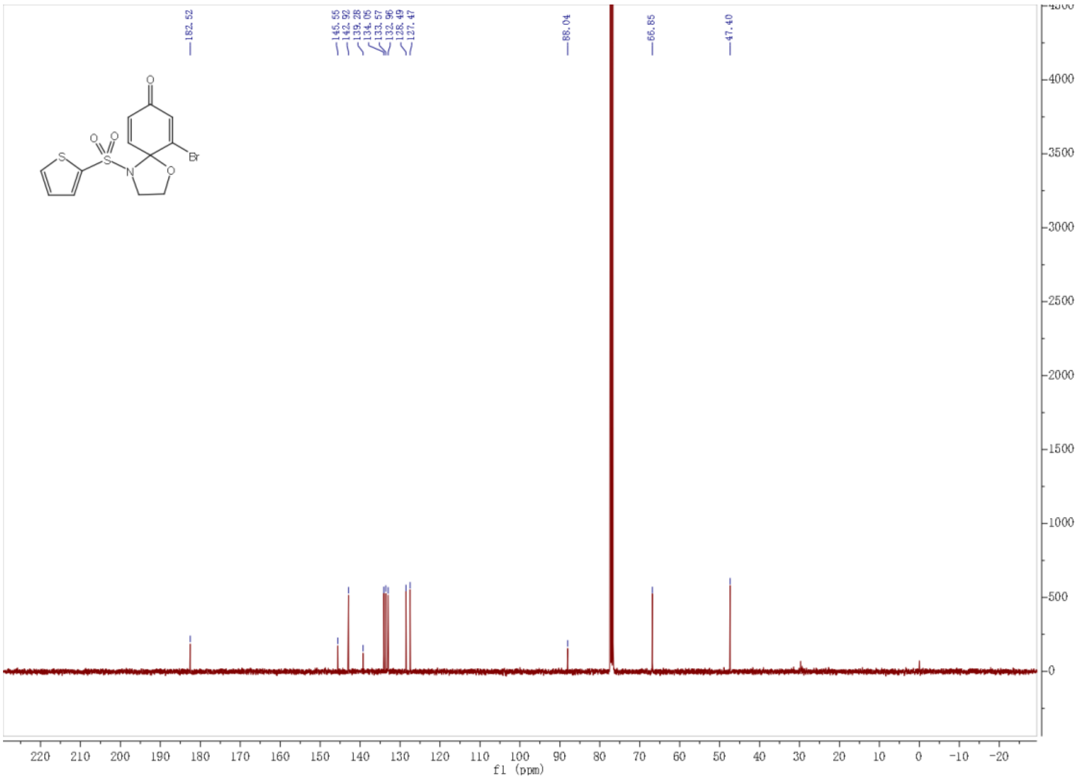

4-((5-Ethynylthiophen-2-yl)sulfonyl)-1-oxa-4-azaspiro[4.5]deca-6,9-dien-8-one (6a)

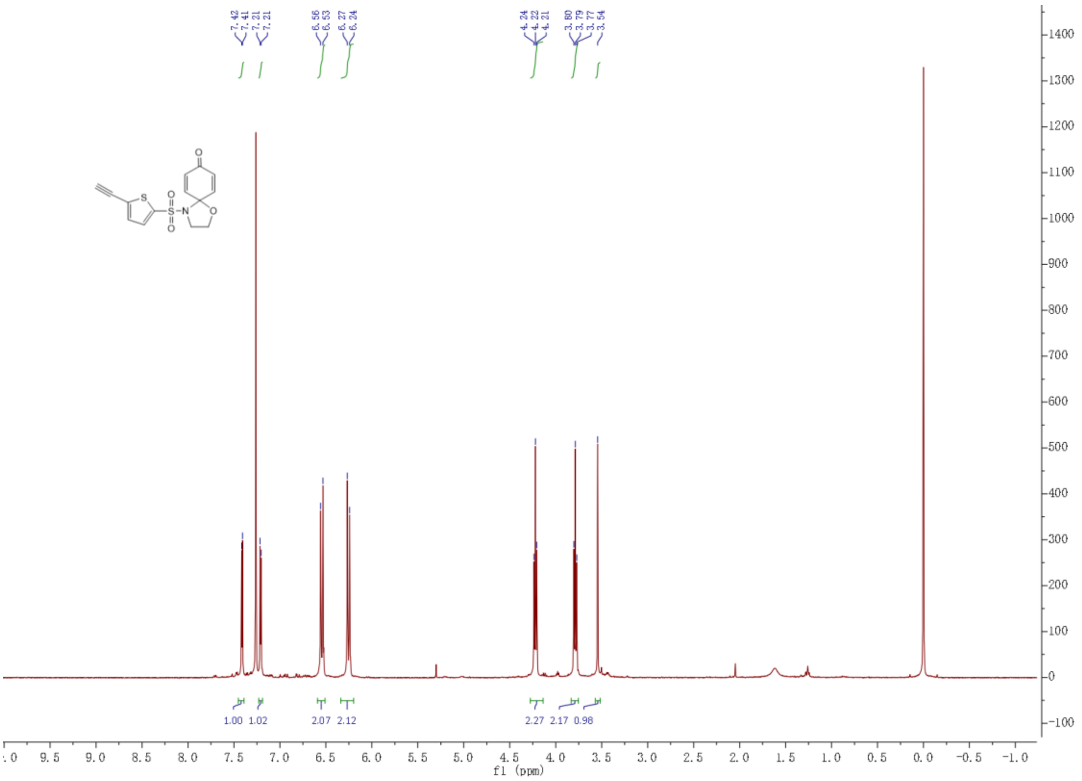

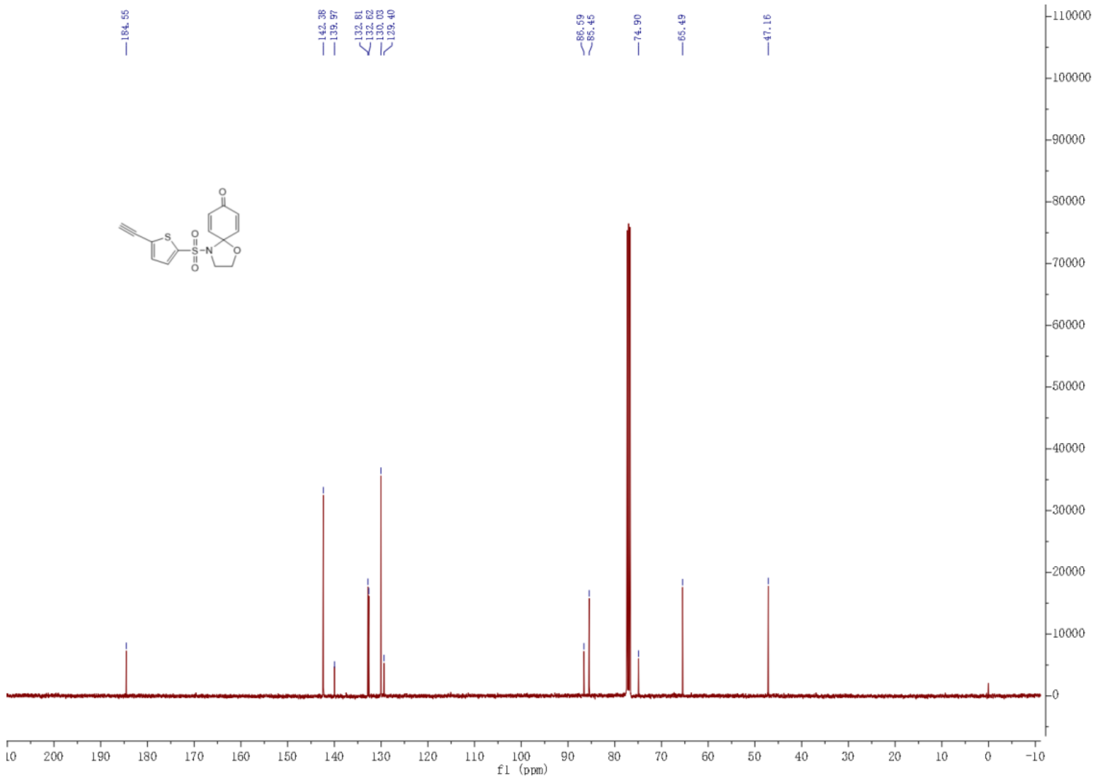

4-((5-Ethynylthiophen-2-yl)sulfonyl)-6-fluoro-1-oxa-4-azaspiro[4.5]deca-6,9-dien-8-one (6b)

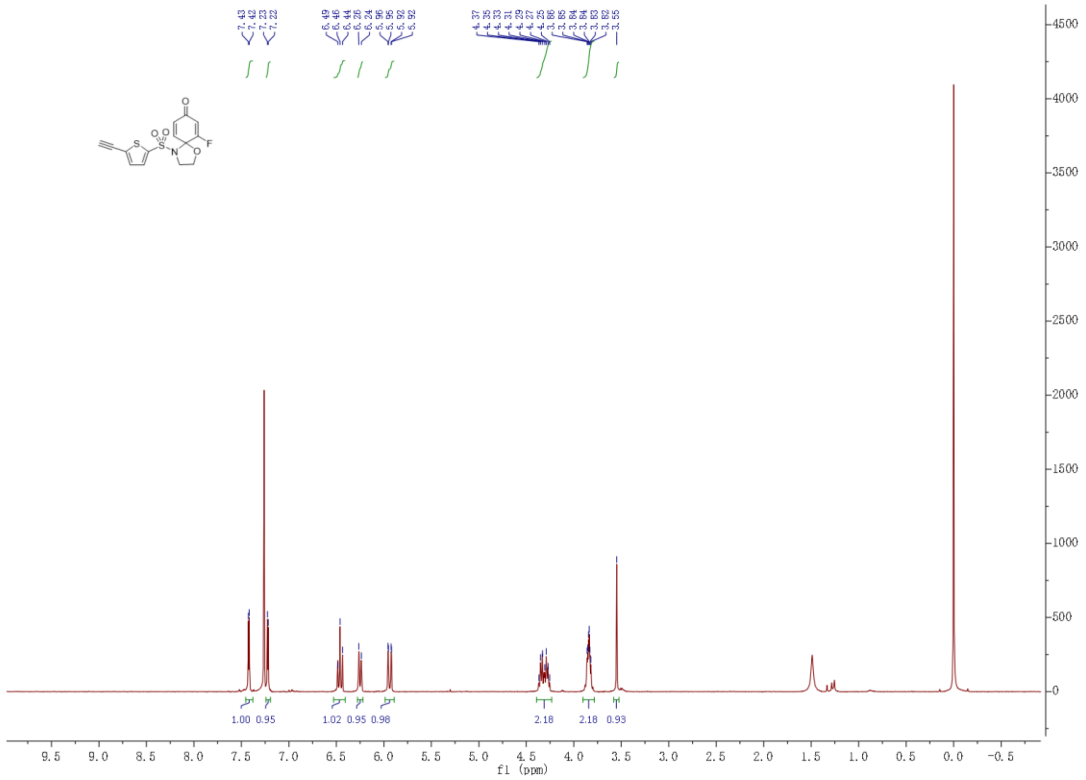

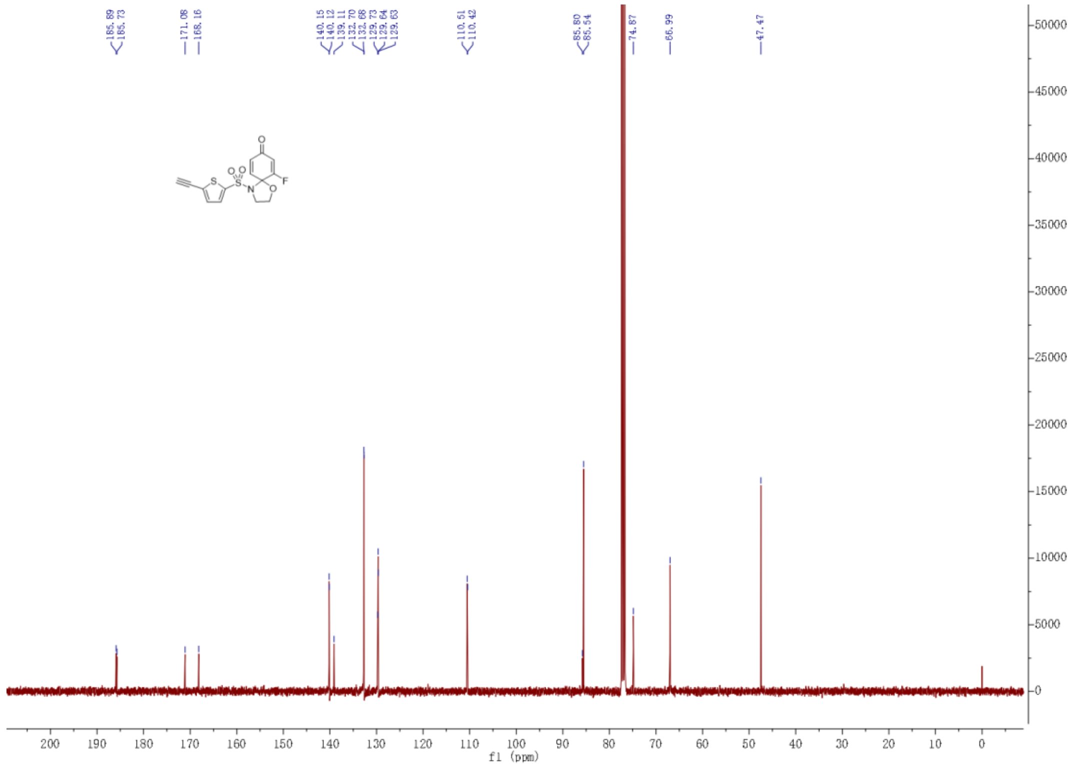

4-((5-Ethynylthiophen-2-yl)sulfonyl)-6-methyl-1-oxa-4-azaspiro[4.5]deca-6,9-dien-8-one (6c)

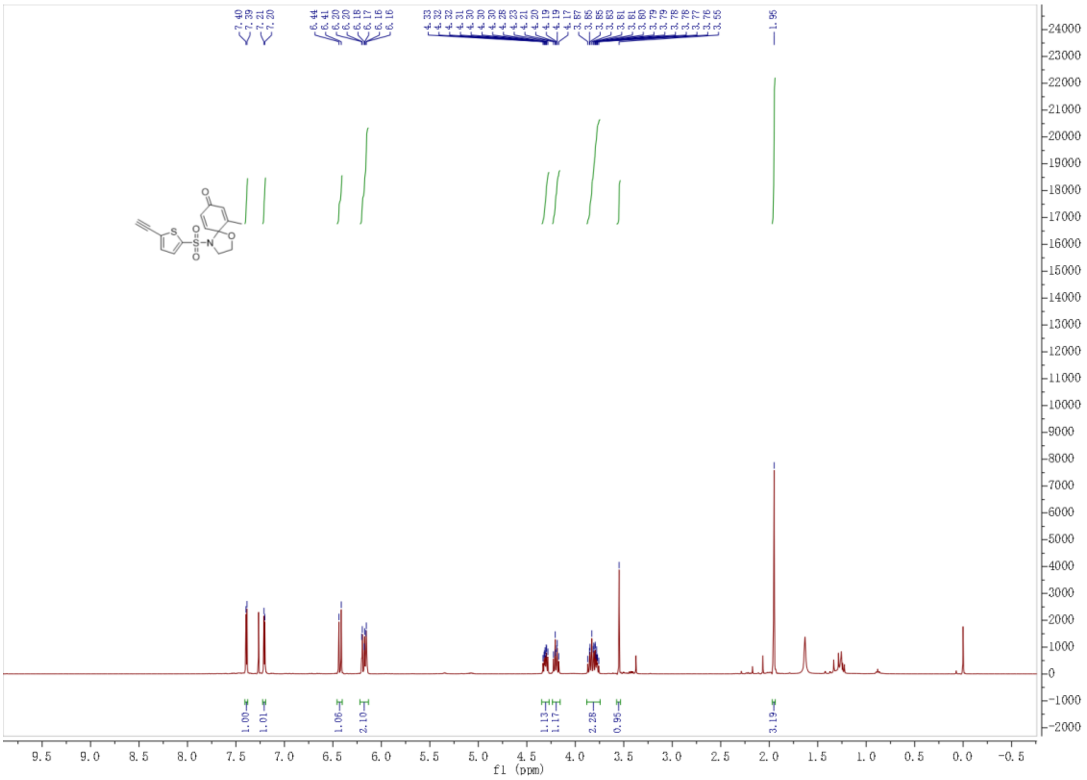

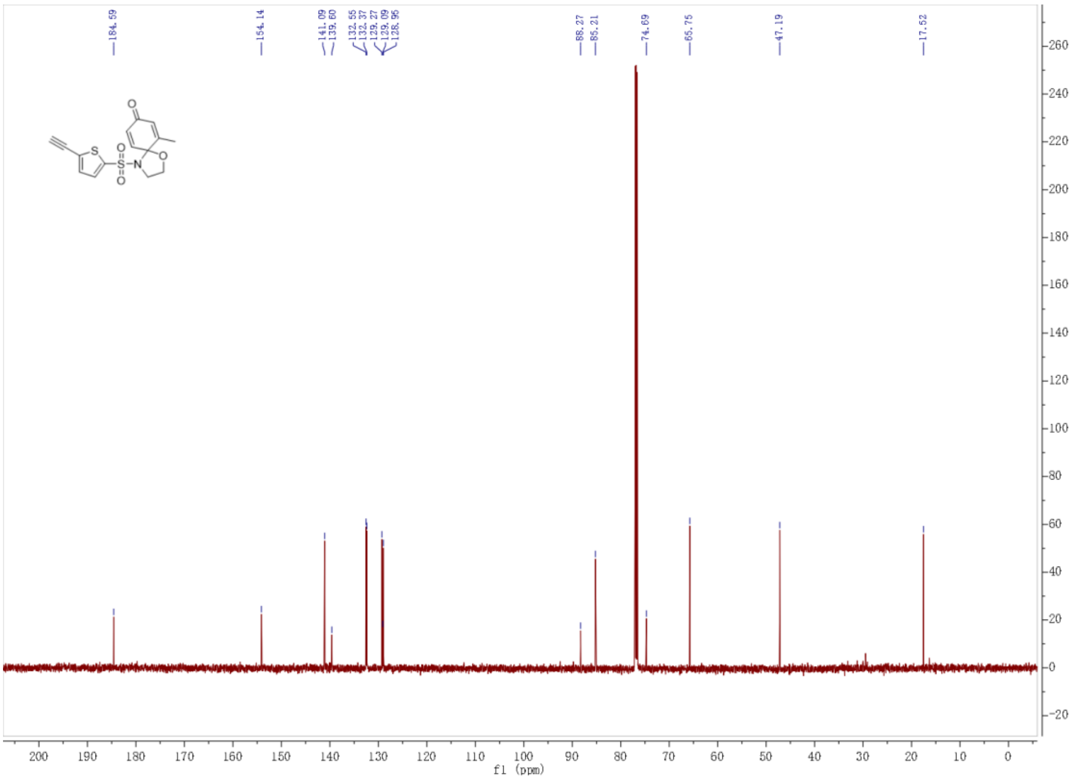

6-Bromo-4-((5-ethynylthiophen-2-yl)sulfonyl)-1-oxa-4-azaspiro[4.5]deca-6,9-dien-8-one (6d)

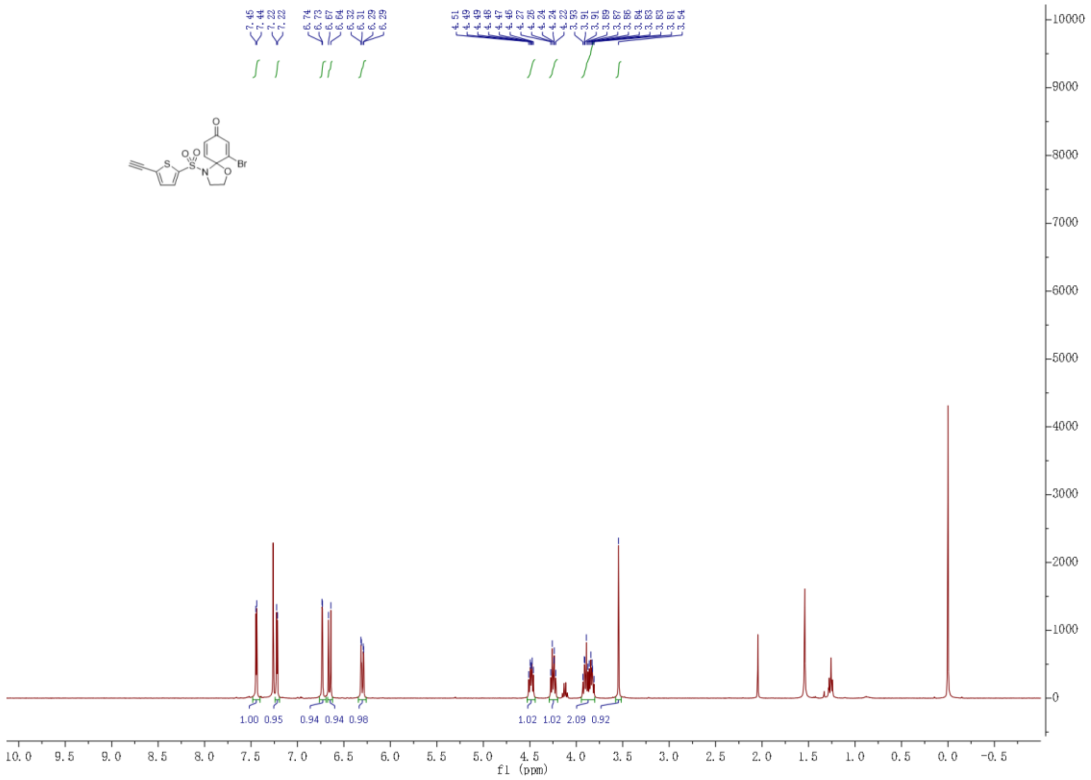

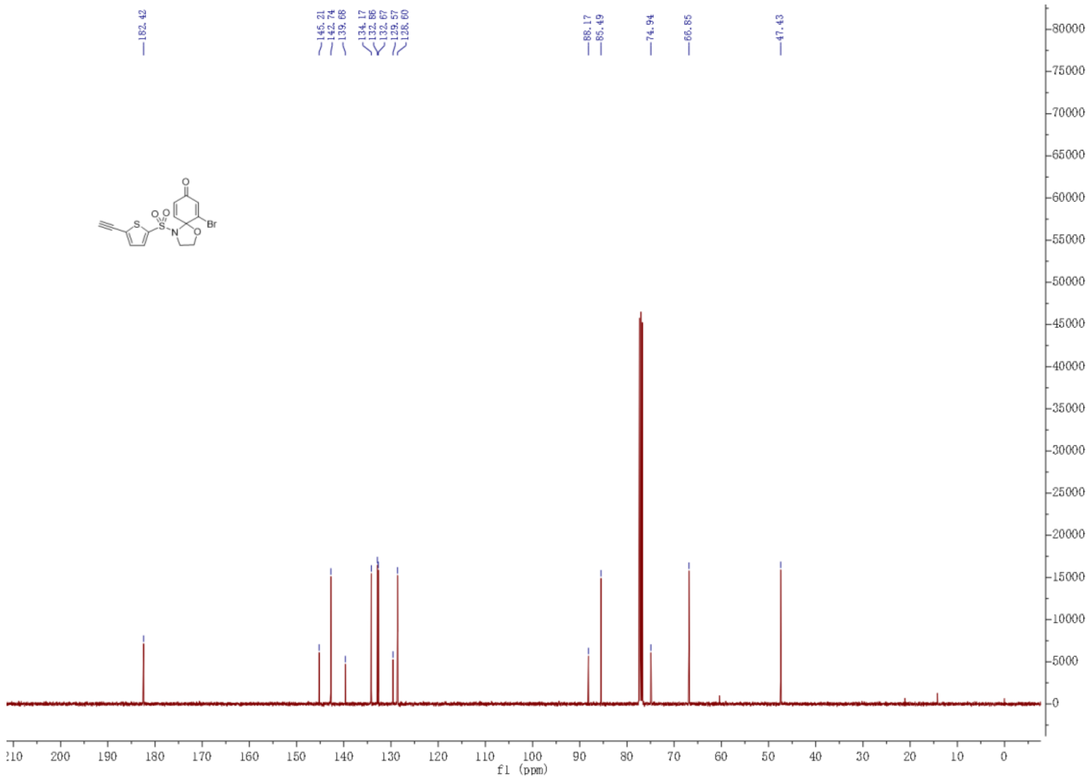

6-Chloro-4-((5-ethynylthiophen-2-yl)sulfonyl)-1-oxa-4-azaspiro[4.5]deca-6,9-dien-8-one (6e)

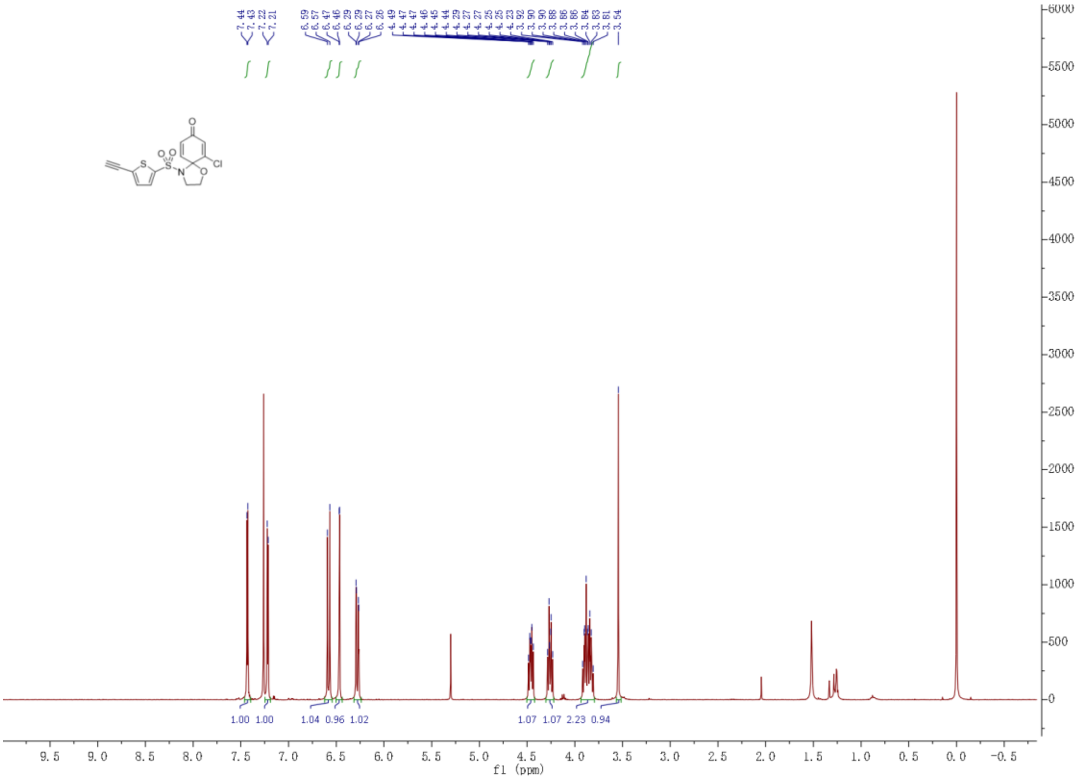

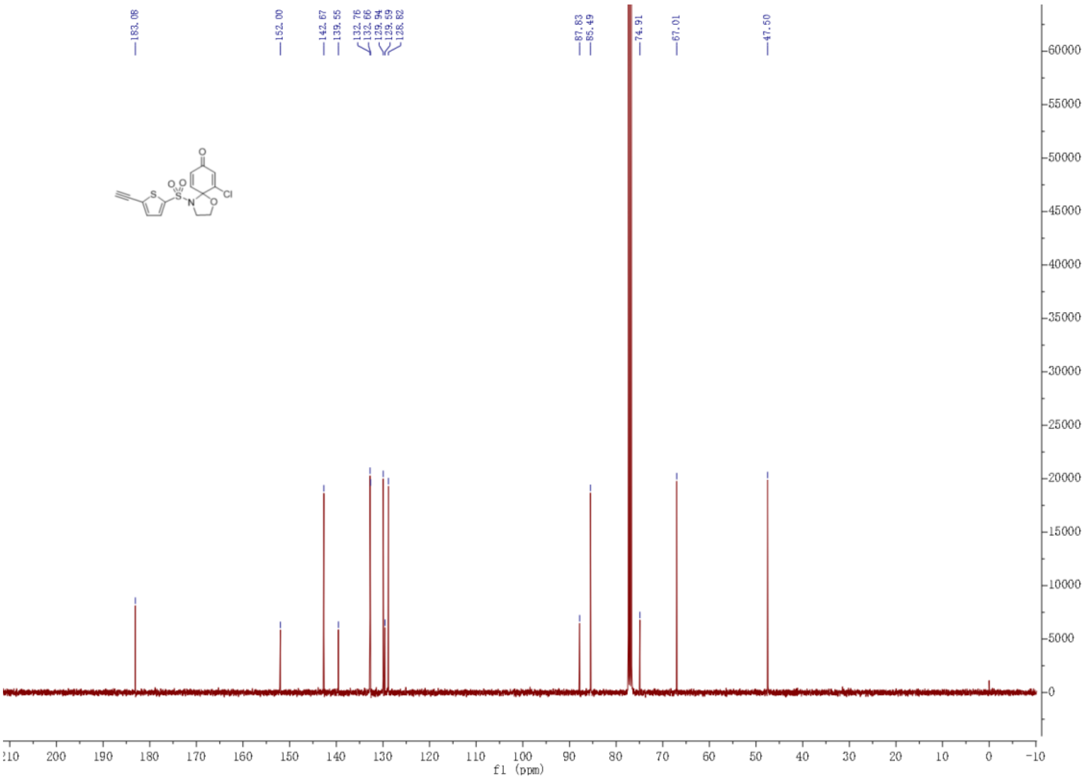

7-Chloro-4-((5-ethynylthiophen-2-yl)sulfonyl)-1-oxa-4-azaspiro[4.5]deca-6,9-dien-8-one (6f)

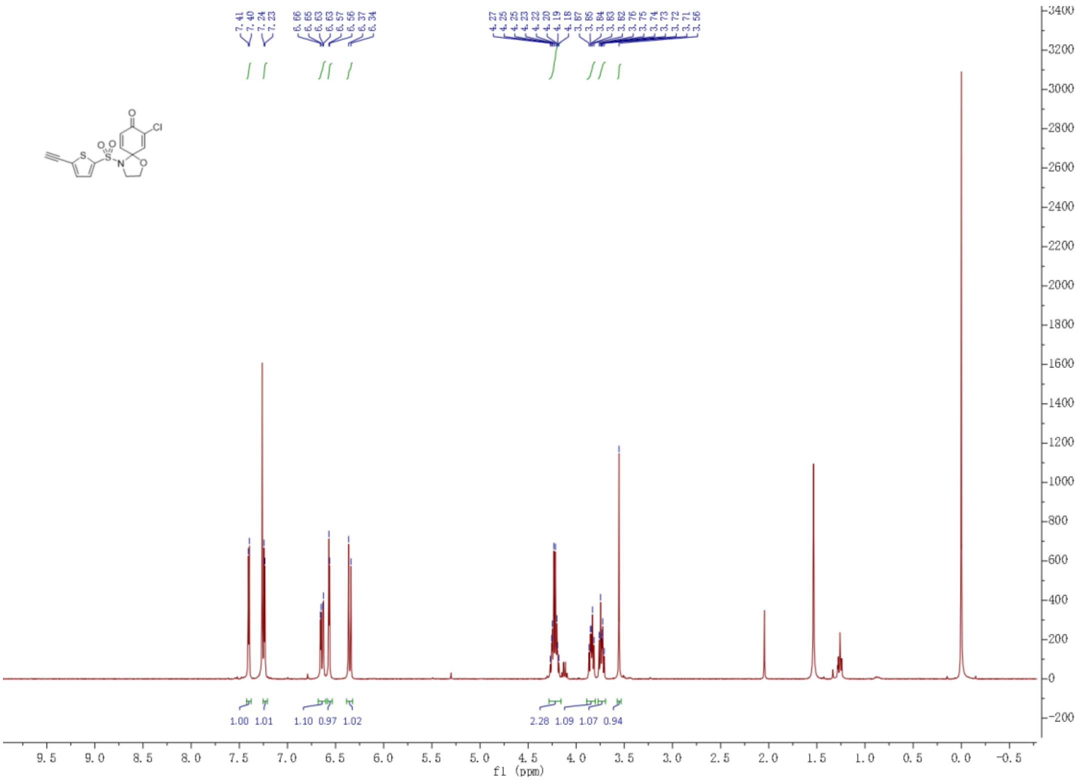

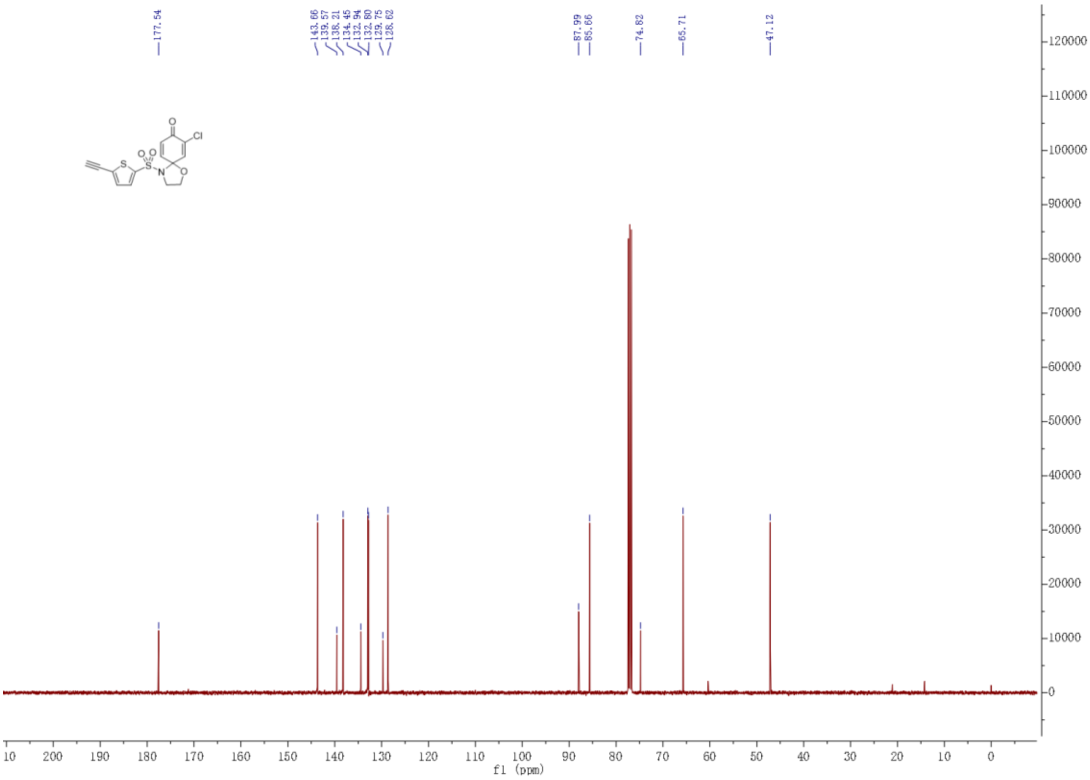

4-((5-Ethynylthiophen-2-yl)sulfonyl)-6,7-dimethyl-1-oxa-4-azaspiro[4.5]deca-6,9-dien-8-one (6g)

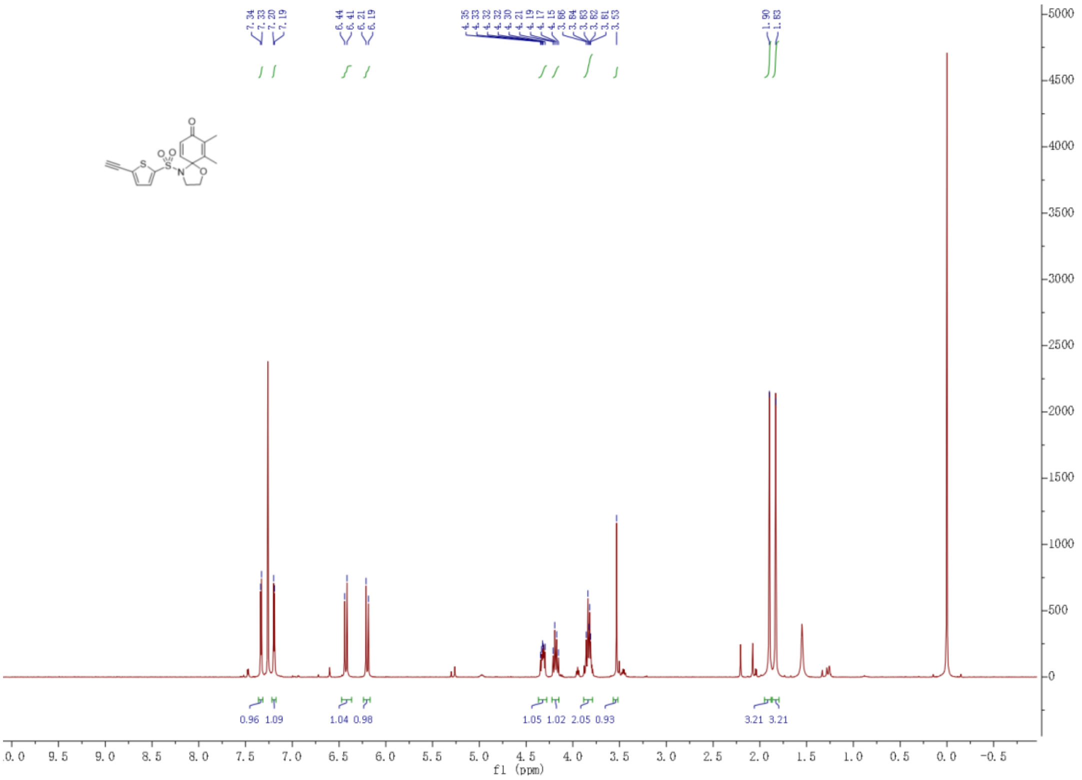

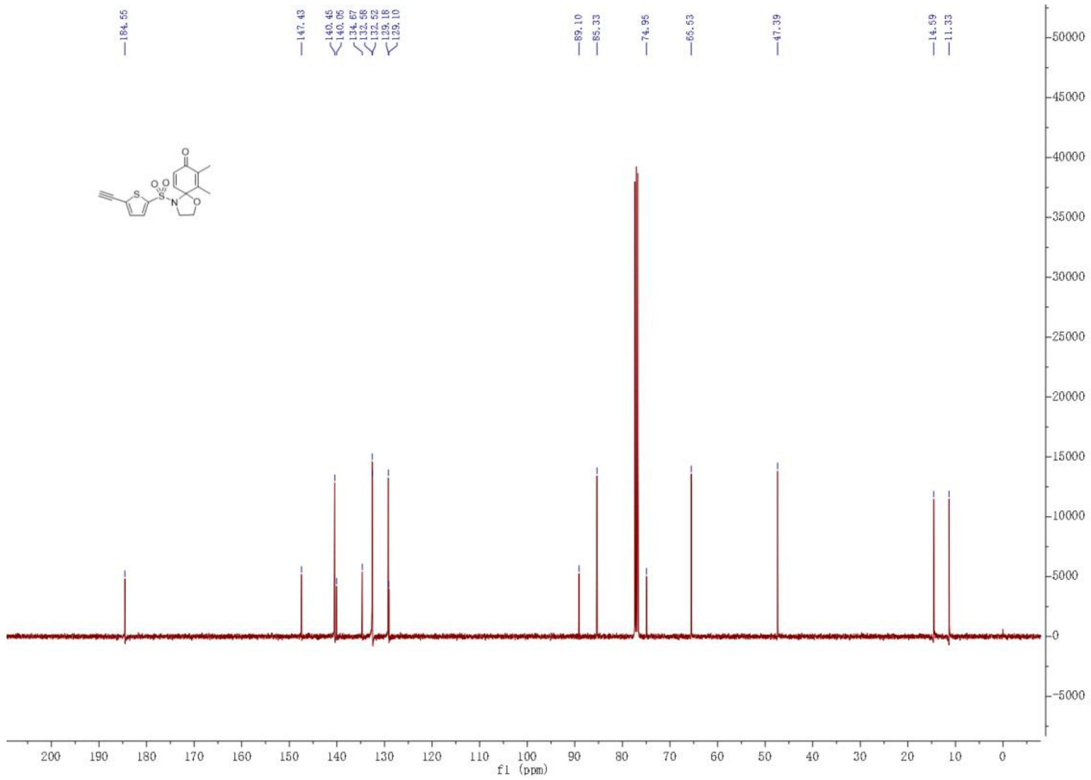

3'-((5-Ethynylthiophen-2-yl)sulfonyl)-4H-spiro[naphthalene-1,2'-oxazolidin]-4-one (6h)

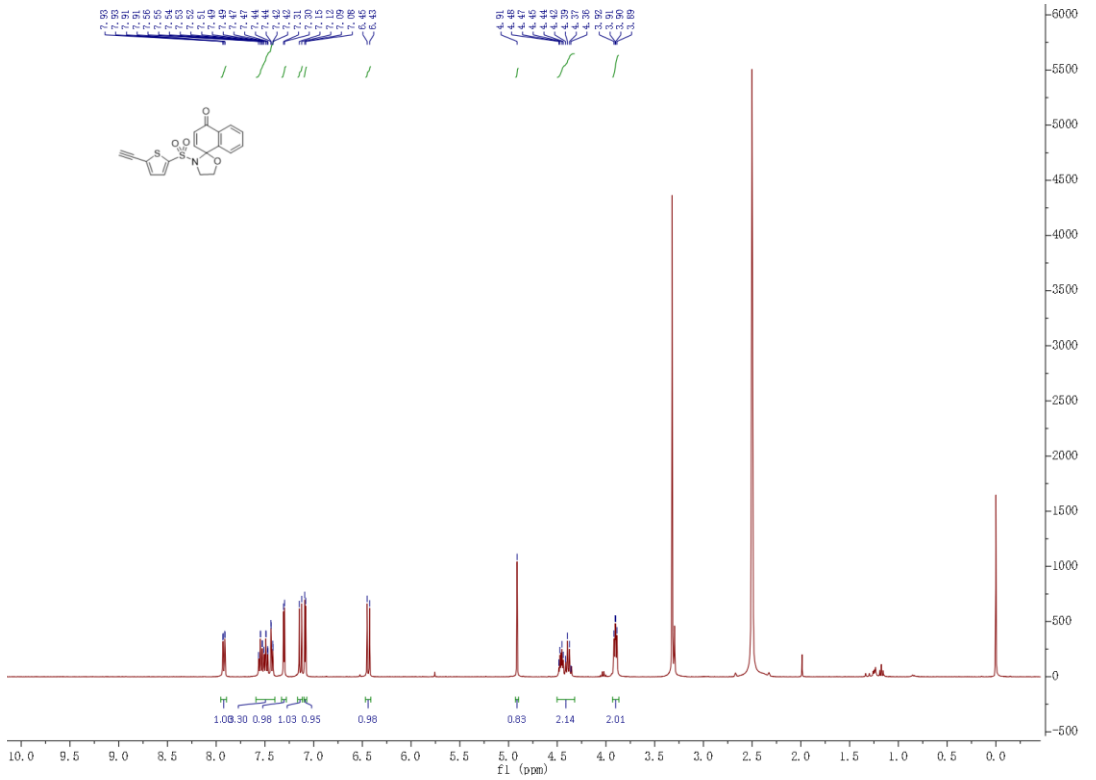

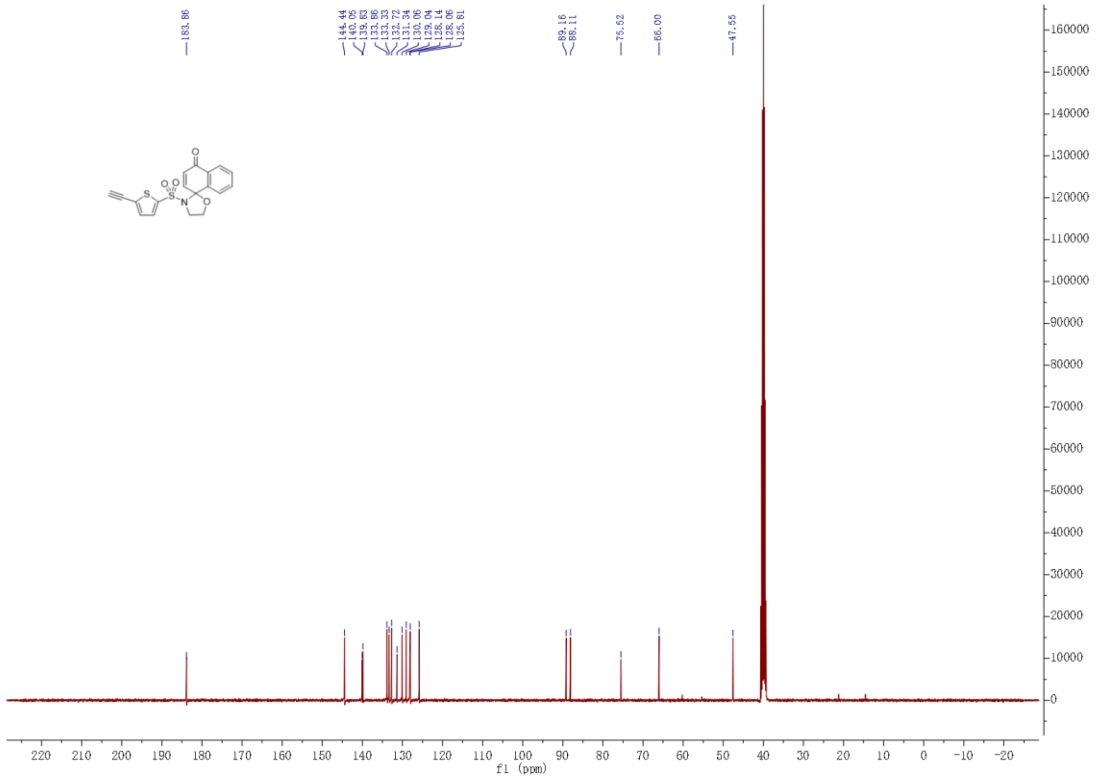

4-((6-((Trimethylsilyl)ethynyl)pyridin-3-yl)sulfonyl)-1-oxa-4-azaspiro[4.5]deca-6,9-dien-8-one (6i)

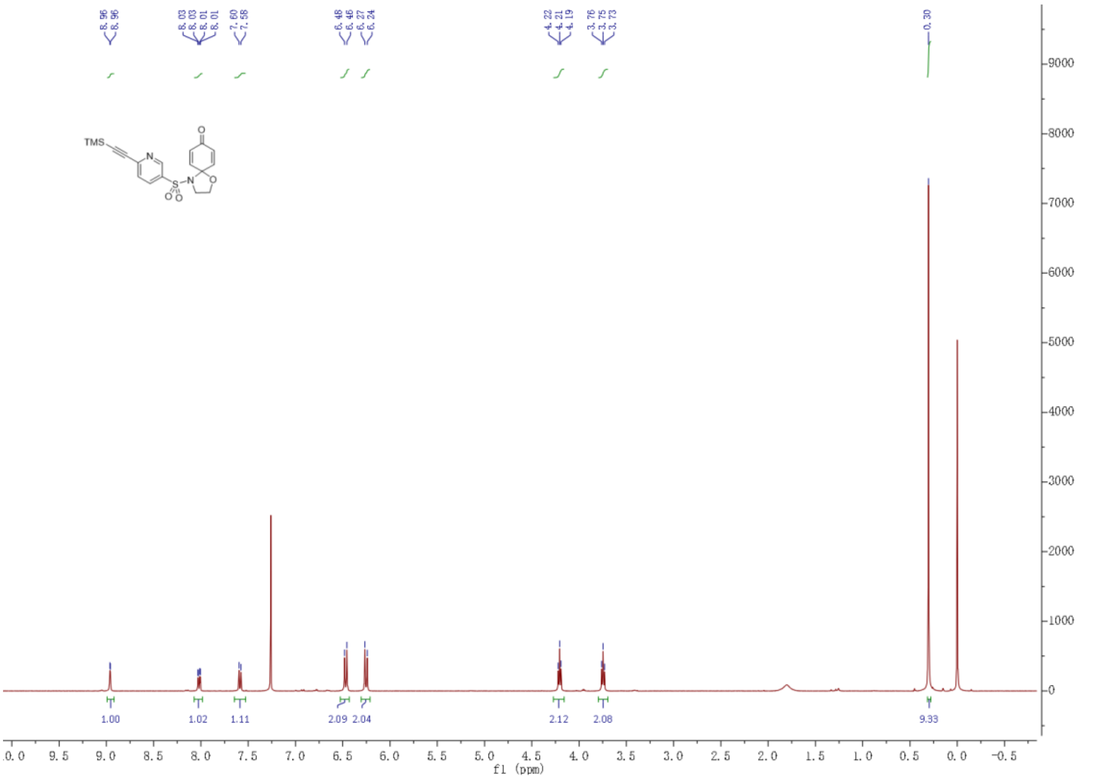

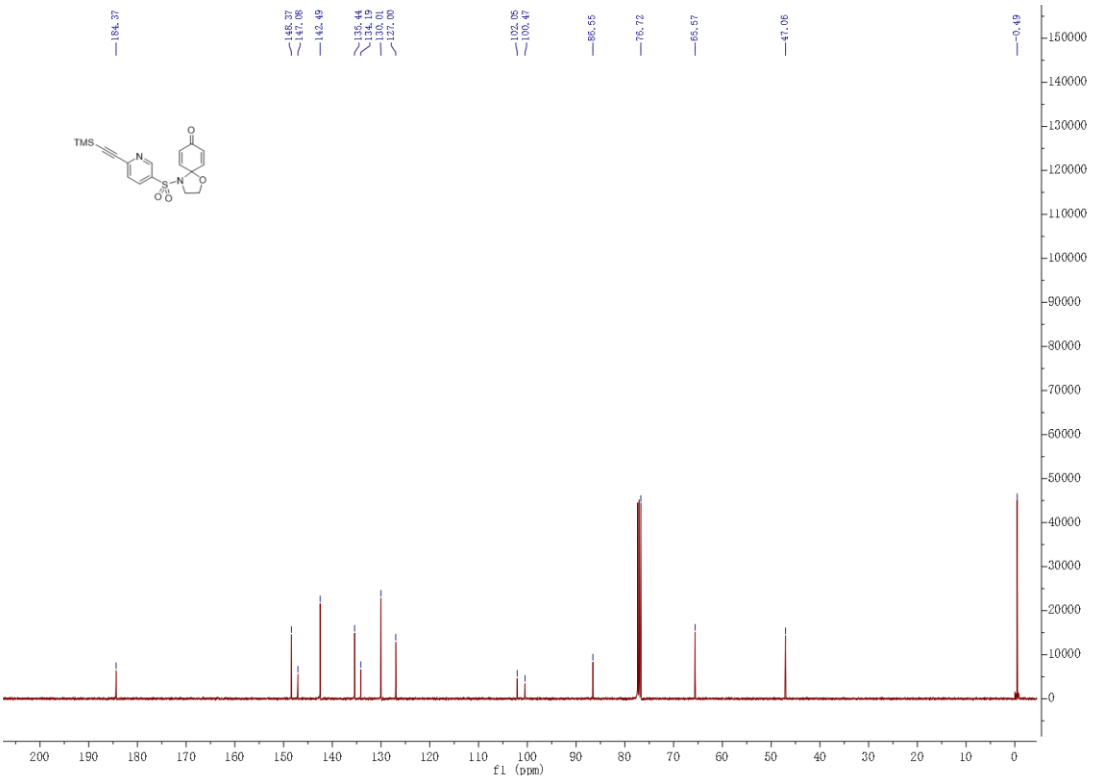

(2R,3R,4S,5R,6R)-2-(Acetoxymethyl)-6-(4-(5-((8-oxo-1-oxa-4-azaspiro[4.5]deca-6,9-dien-4-yl)sulfonyl)thiophen-2-yl)-1*H*-1,2,3-triazol-1-yl)tetrahydro-2*H*-pyran-3,4,5-triyl triacetate (7a)

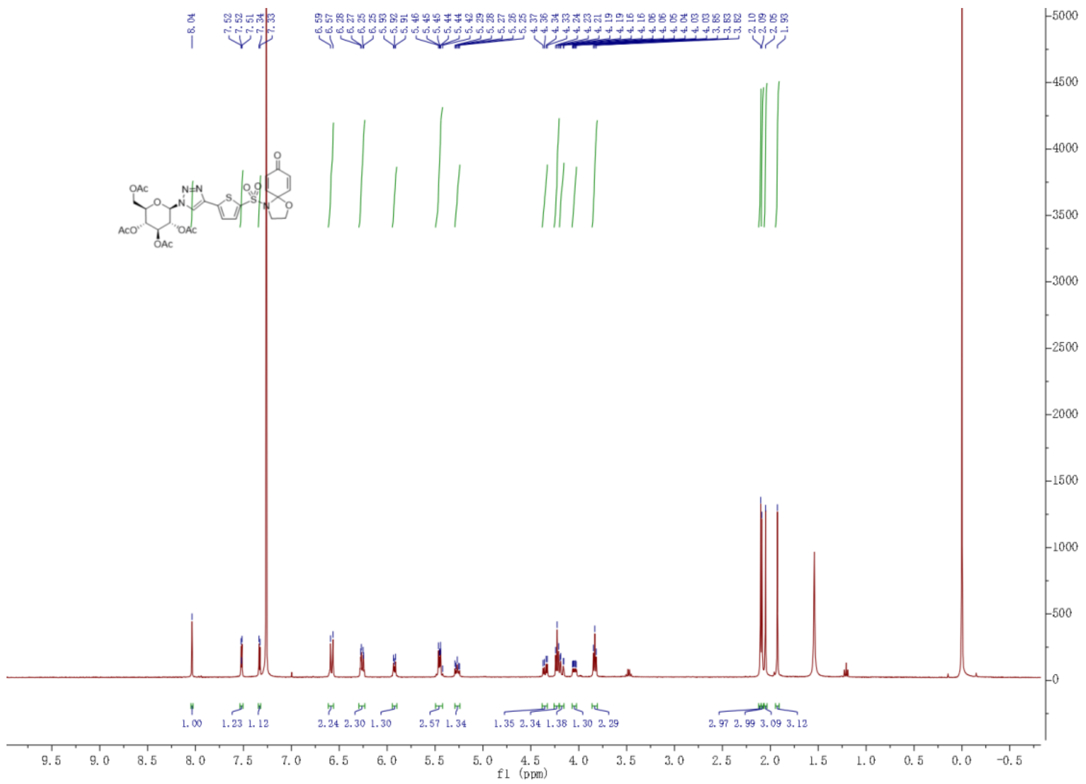

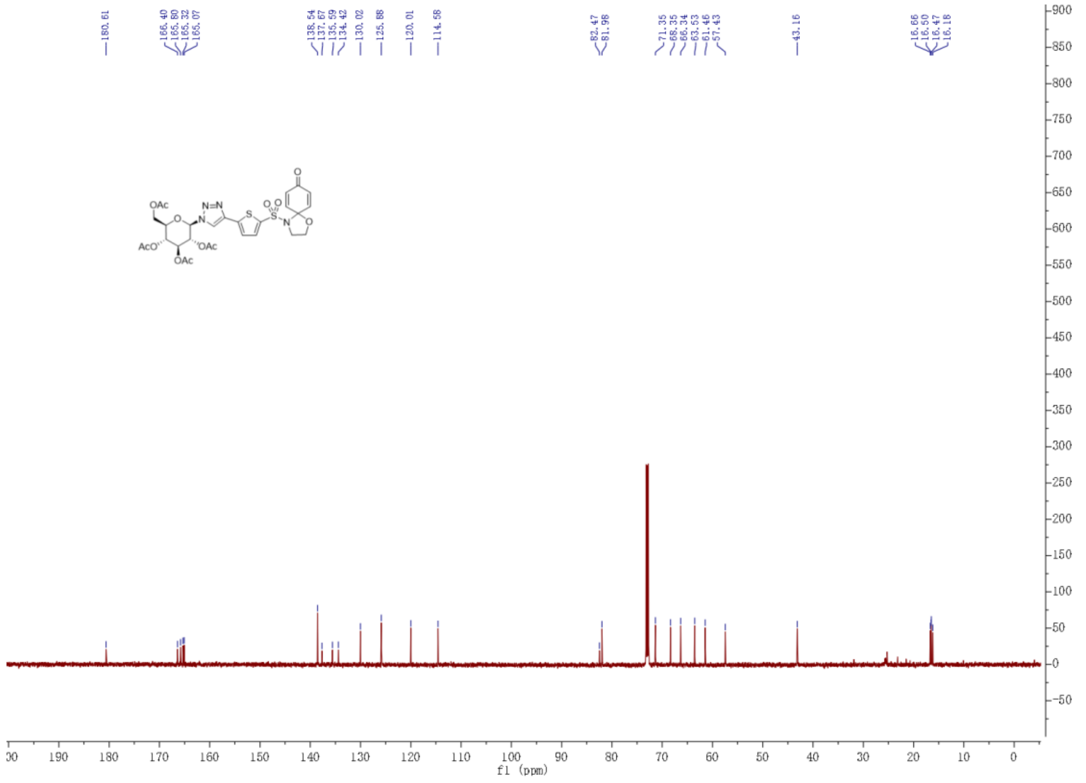

(2R,3R,4S,5R,6R)-2-(Acetoxymethyl)-6-(4-(5-((6-fluoro-8-oxo-1-oxa-4-azaspiro[4.5]deca-6,9-dien-4-yl)sulfonyl)thiophen-2-yl)-1*H*-1,2,3-triazol-1-yl)tetrahydro-2*H*-pyran-3,4,5-triyl triacetate (7b)

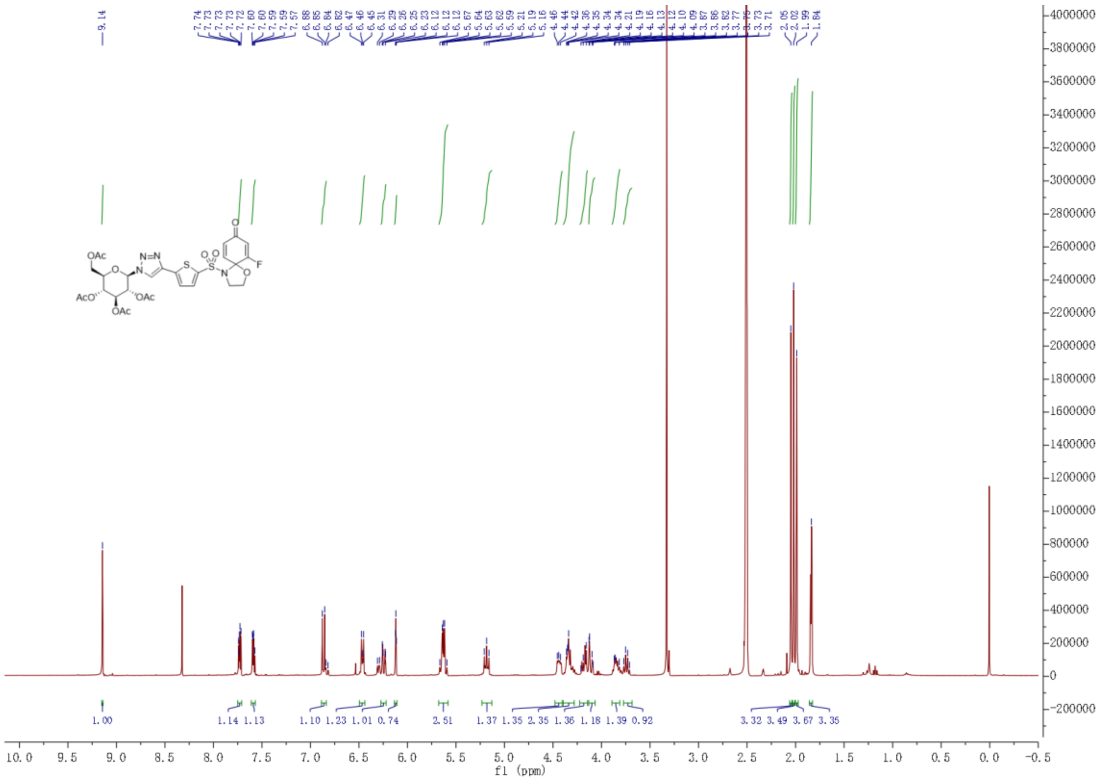

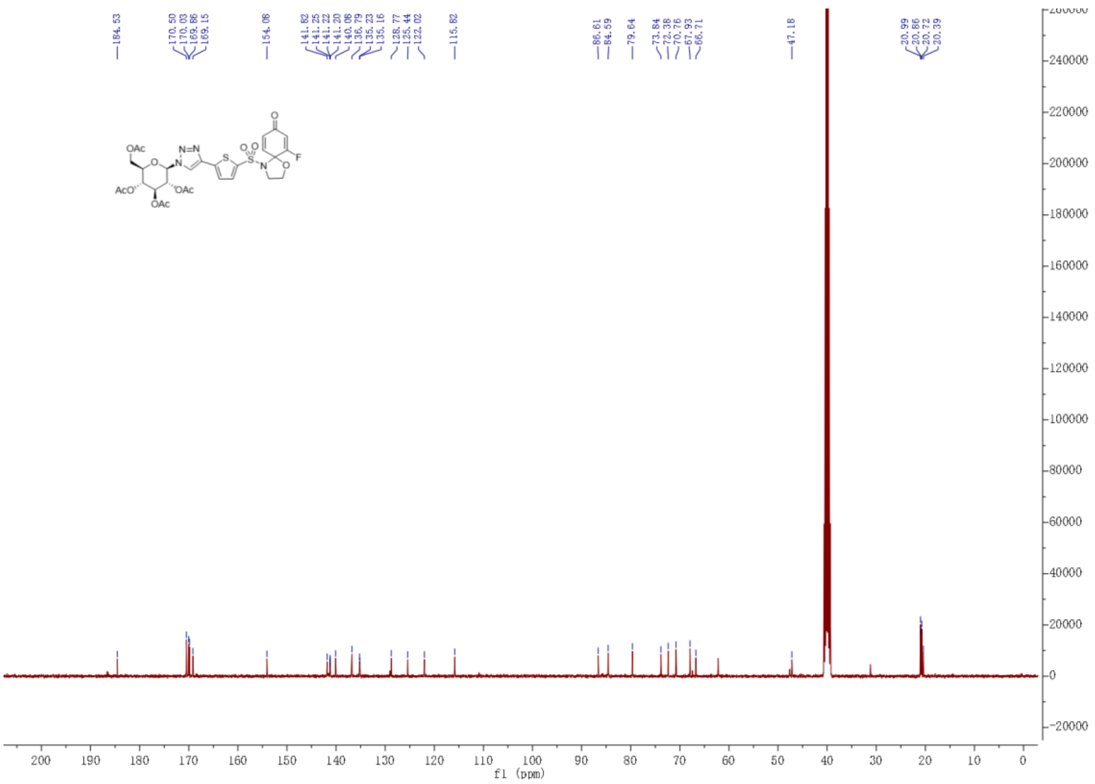

(2R,3R,4S,5R,6R)-2-(Acetoxymethyl)-6-(4-(5-((6-methyl-8-oxo-1-oxa-4-azaspiro[4.5]deca-6,9-dien -4-yl)sulfonyl)thiophen-2-yl)-1*H*-1,2,3-triazol-1-yl)tetrahydro-2*H*-pyran-3,4,5-triyl triacetate (7c)

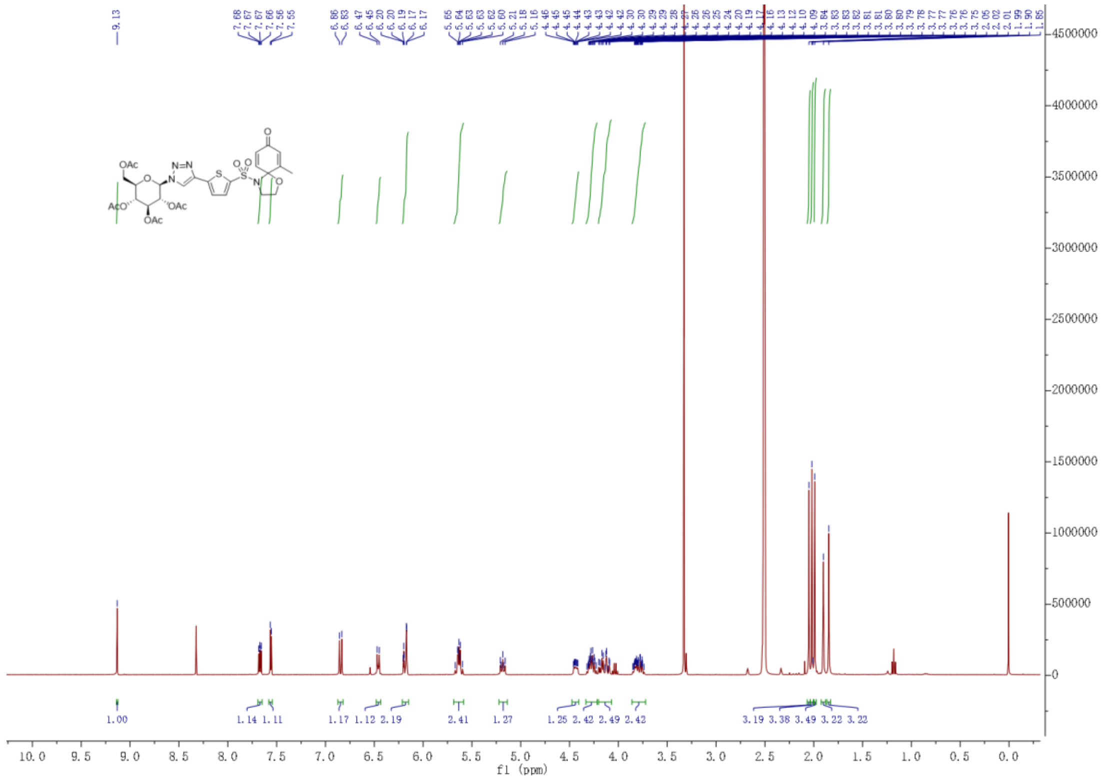

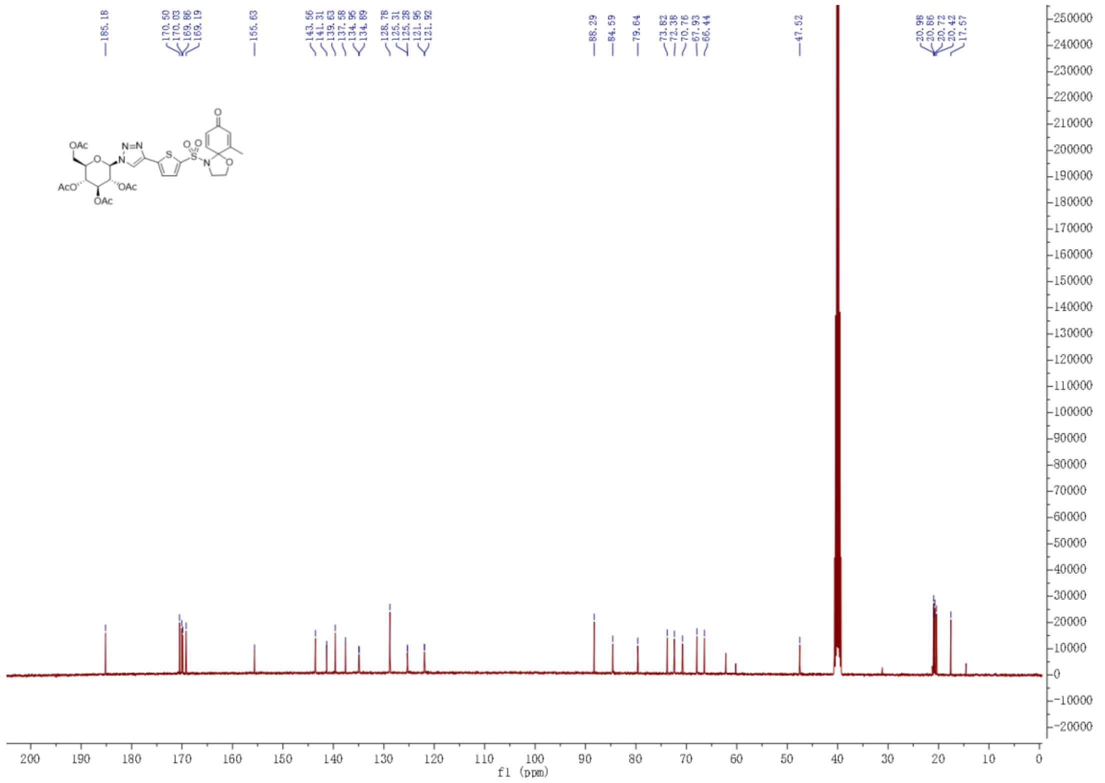

(2R,3R,4S,5R,6R)-2-(Acetoxymethyl)-6-(4-(5-((6-bromo-8-oxo-1-oxa-4-azaspiro[4.5]deca-6,9-dien

-4-yl)sulfonyl)thiophen-2-yl)-1*H*-1,2,3-triazol-1-yl)tetrahydro-2*H*-pyran-3,4,5-triyl triacetate (7d)

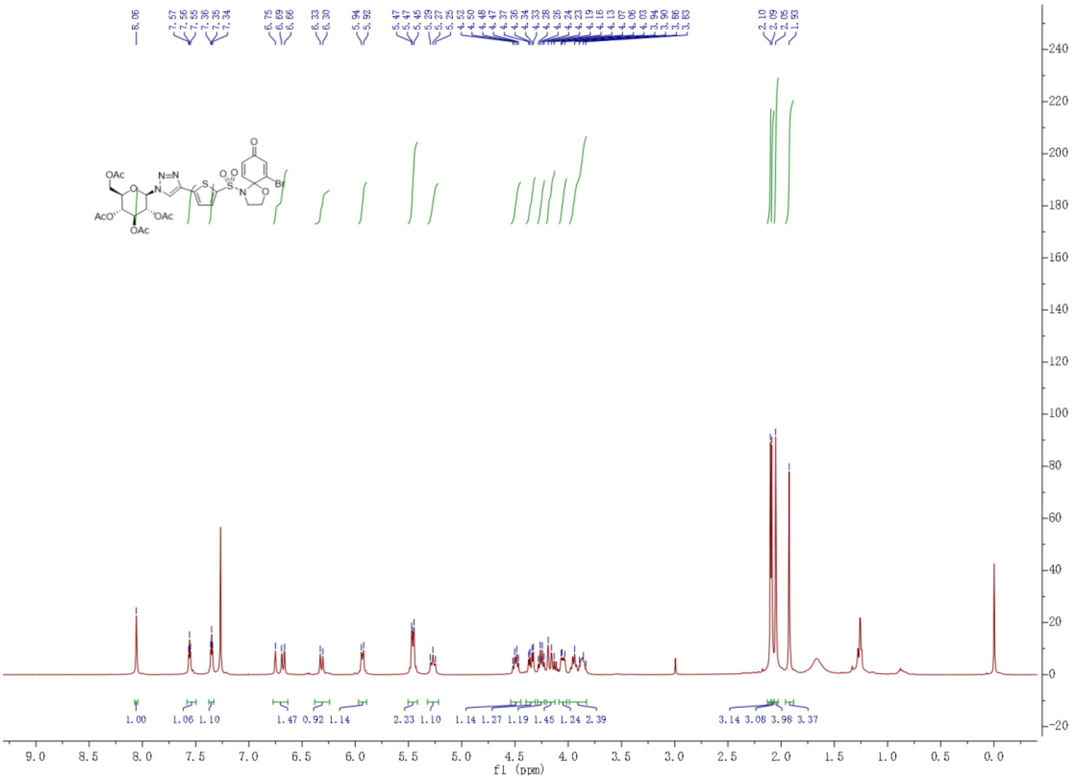

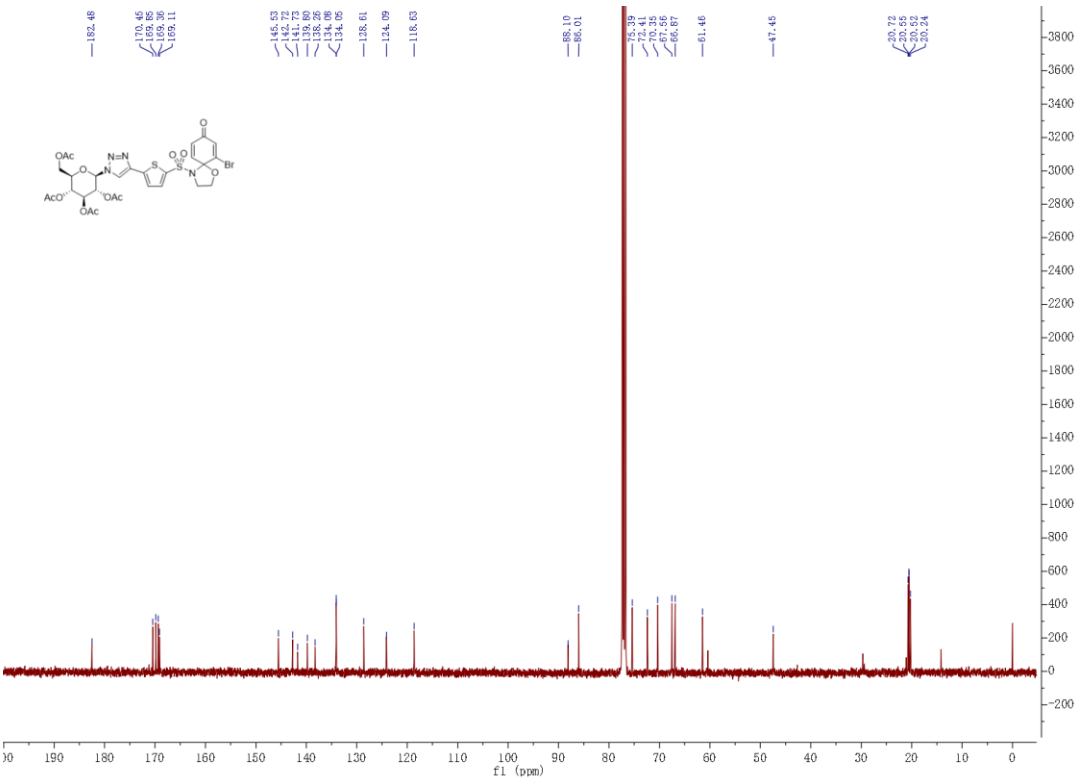

(2R,3R,4S,5R,6R)-2-(Acetoxymethyl)-6-(4-(5-((6-chloro-8-oxo-1-oxa-4-azaspiro[4.5]deca-6,9-dien -4-yl)sulfonyl)thiophen-2-yl)-1*H*-1,2,3-triazol-1-yl)tetrahydro-2*H*-pyran-3,4,5-triyl triacetate (7e)

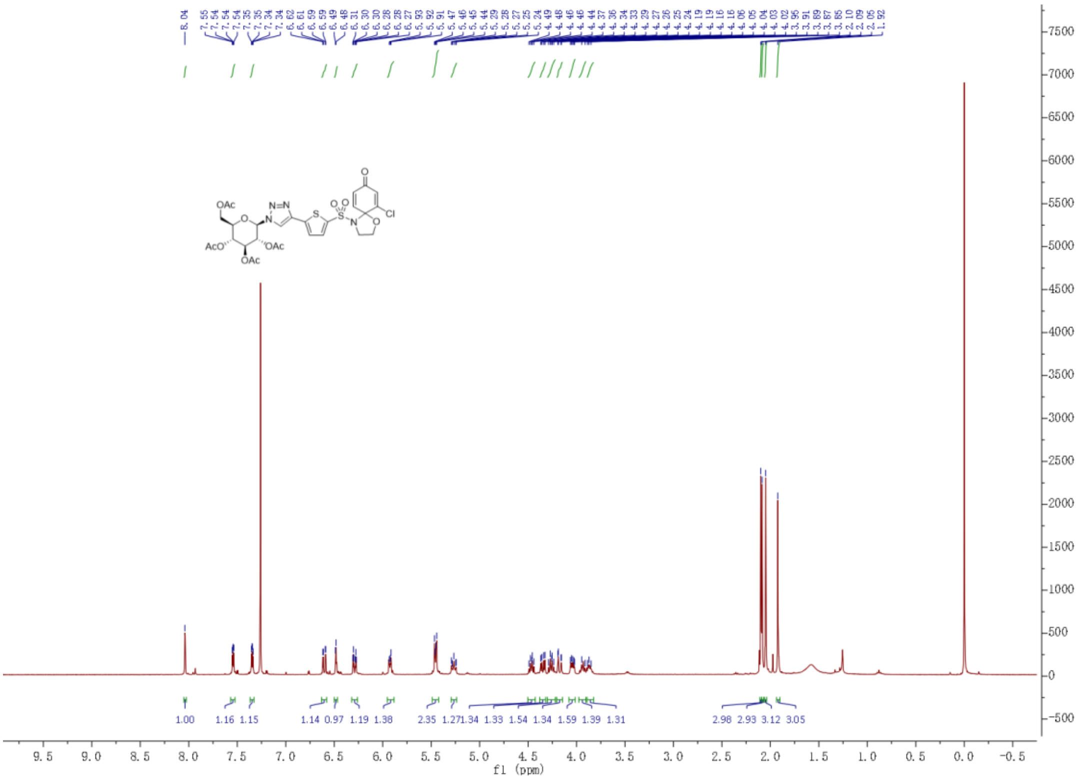

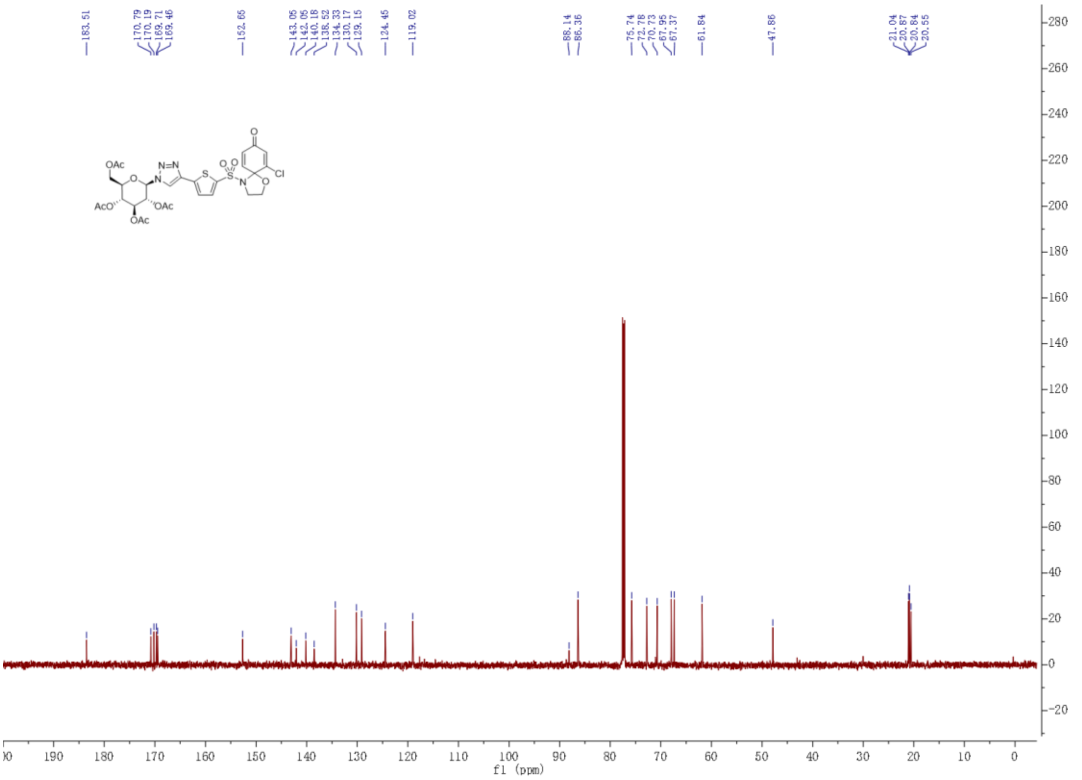

(2R,3R,4S,5R,6R)-2-(Acetoxymethyl)-6-(4-(5-((7-chloro-8-oxo-1-oxa-4-azaspiro[4.5]deca-6,9-dien-4-yl)sulfonyl)thiophen-2-yl)-1*H*-1,2,3-triazol-1-yl)tetrahydro-2*H*-pyran-3,4,5-triyl triacetate (7f)

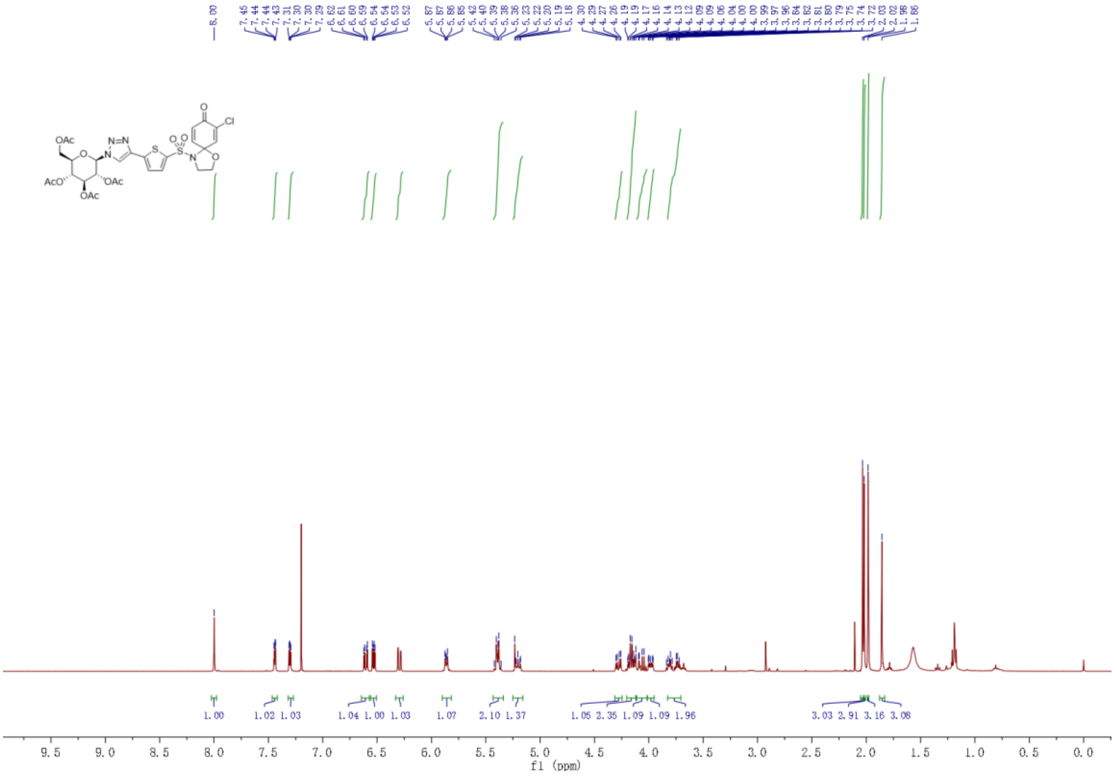

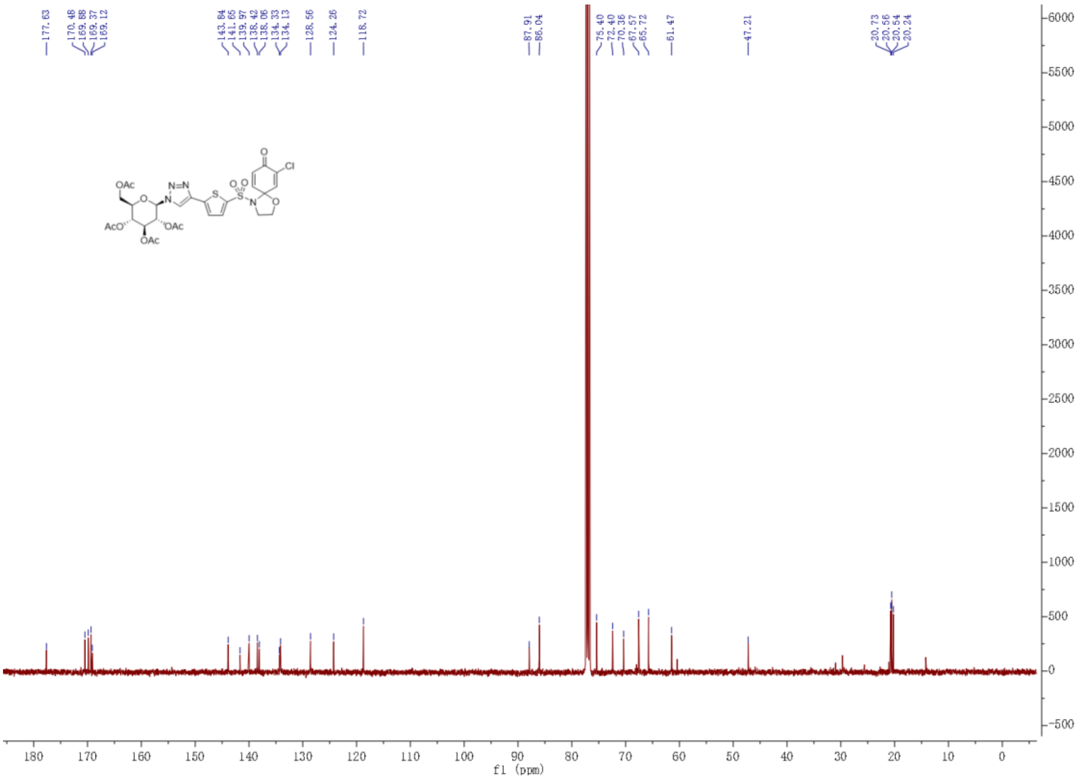

(2R,3R,4S,5R,6R)-2-(Acetoxymethyl)-6-(4-(5-((6,7-dimethyl-8-oxo-1-oxa-4-azaspiro[4.5]deca-6,9-dien-4-yl)sulfonyl)thiophen-2-yl)-1*H*-1,2,3-triazol-1-yl)tetrahydro-2*H*-pyran-3,4,5-triyl triacetate (7g)

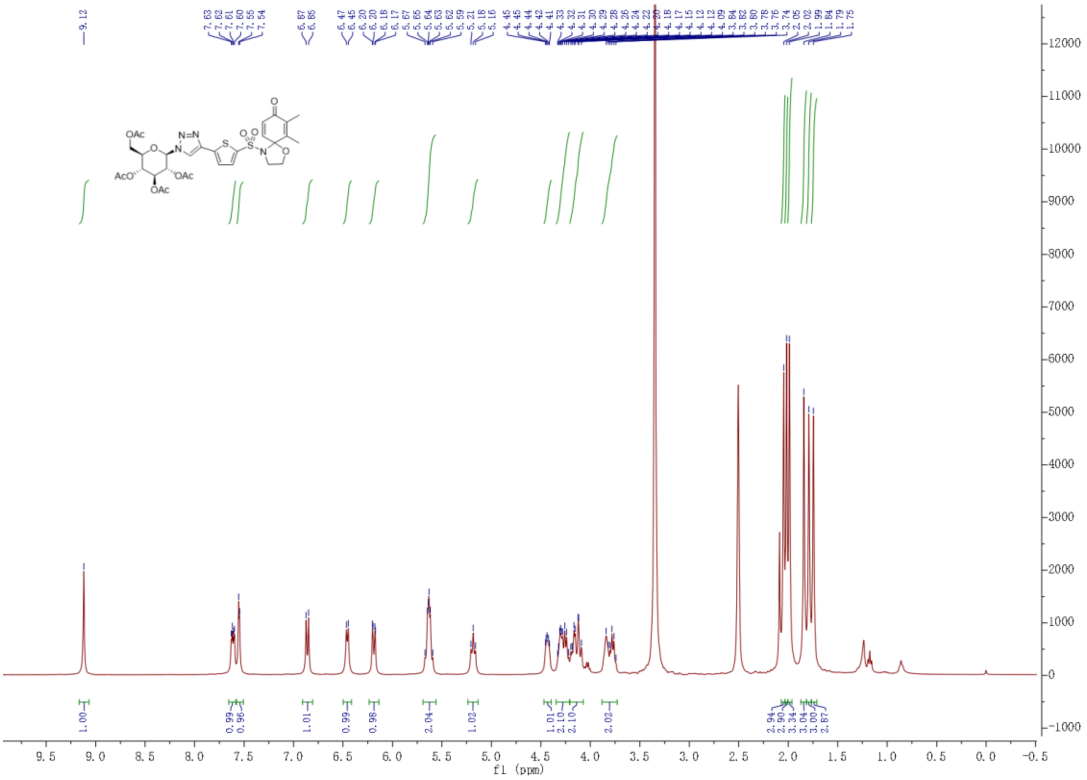

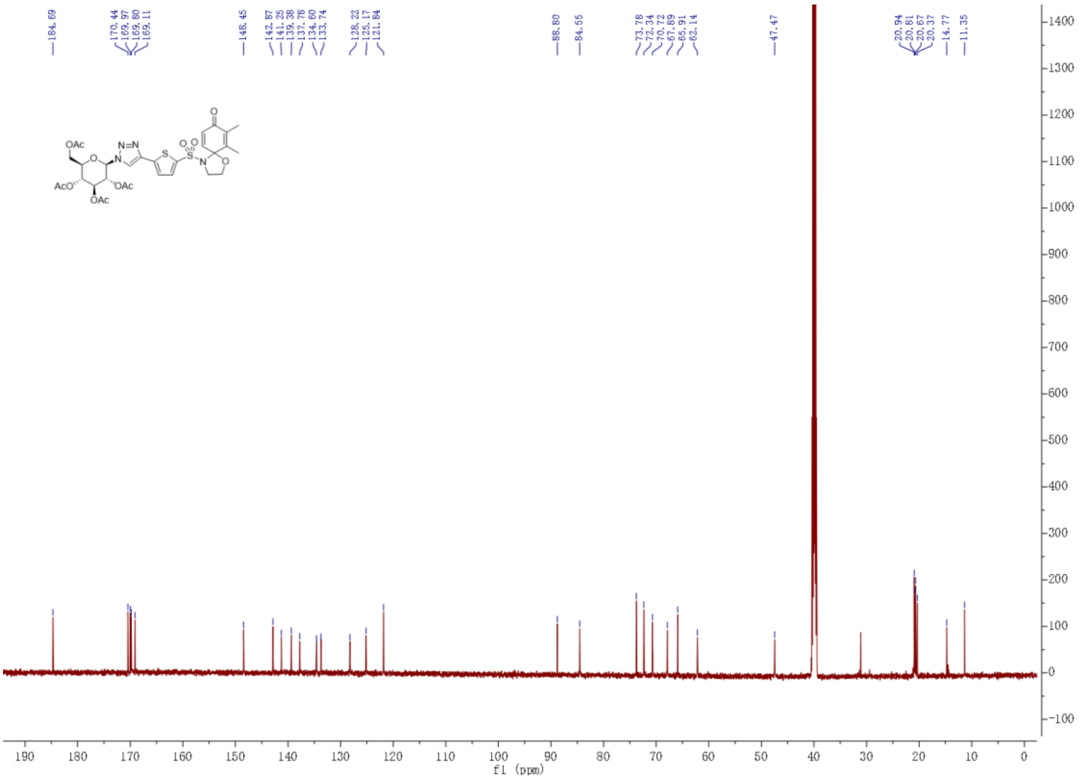

(2R,3R,4S,5R,6R)-2-(Acetoxymethyl)-6-(4-(5-((4-oxo-4*H*-spiro[naphthalene-1,2'-oxazolidin]-3'-yl

)sulfonyl)thiophen-2-yl)-1*H*-1,2,3-triazol-1-yl)tetrahydro-2*H*-pyran-3,4,5-triyl triacetate (7h)**

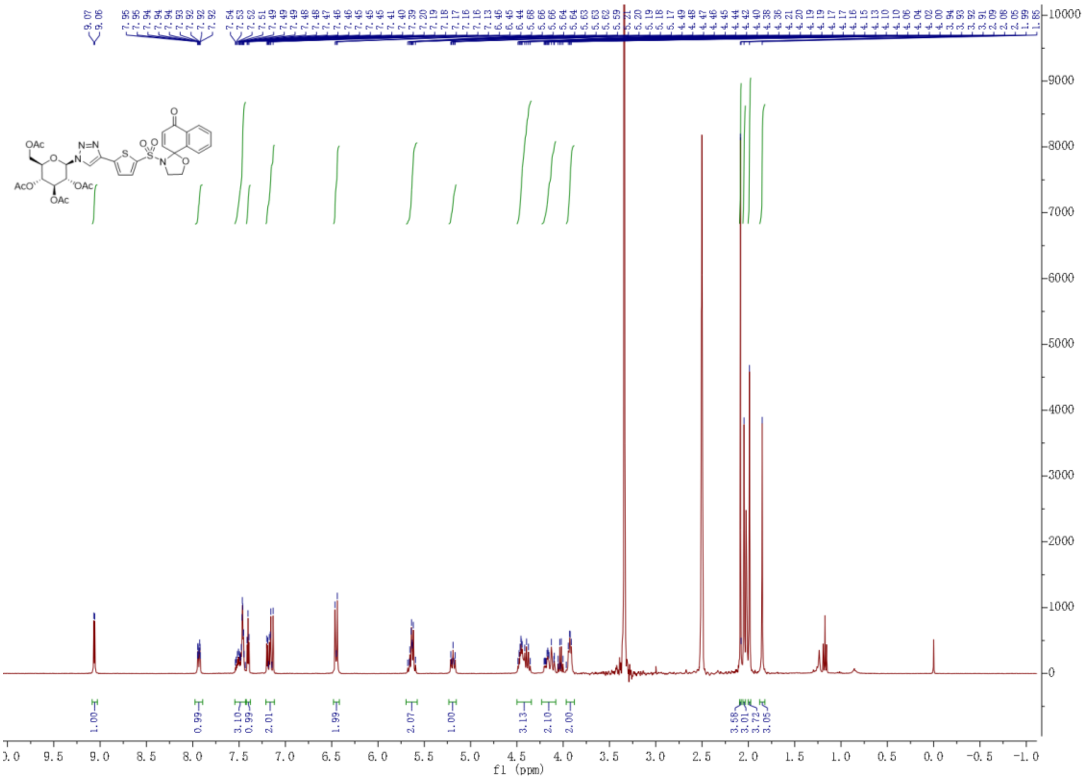

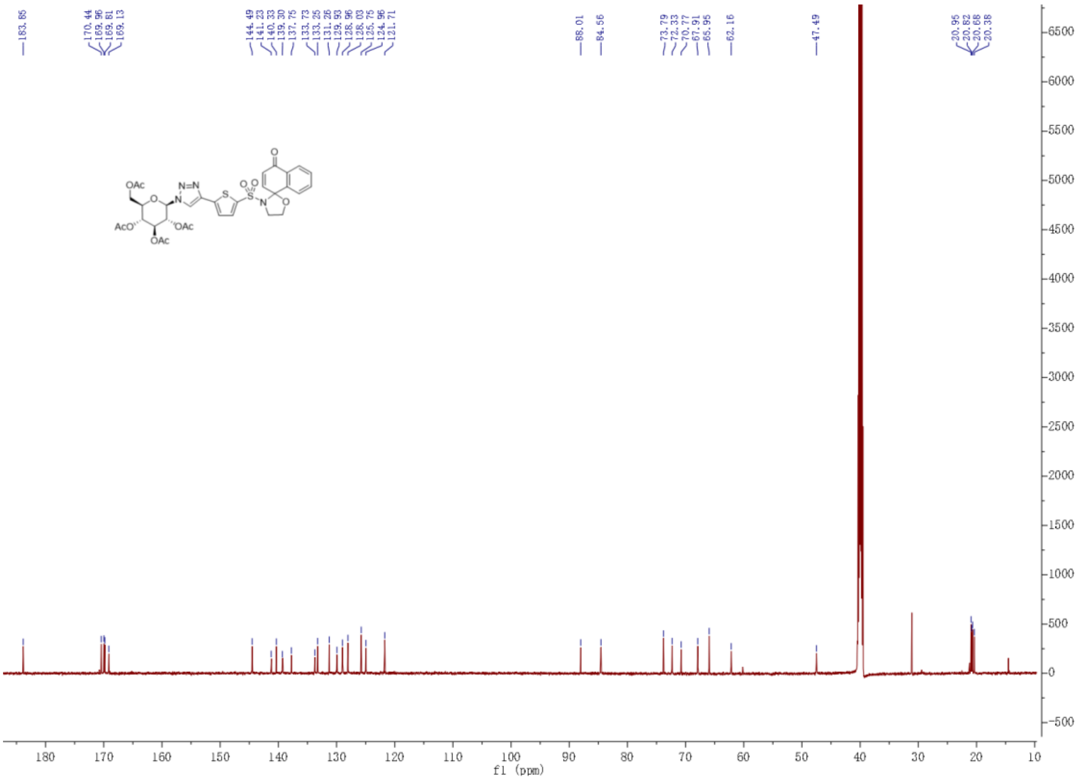

(2R,3R,4S,5S,6S)-2-(Acetoxymethyl)-6-(4-(5-((8-oxo-1-oxa-4-azaspiro[4.5]deca-6,9-dien-4-yl)sulfonyl)thiophen-2-yl)-1H-1,2,3-triazol-1-yl)tetrahydro-2H-pyran-3,4,5-triyl triacetate (7i)

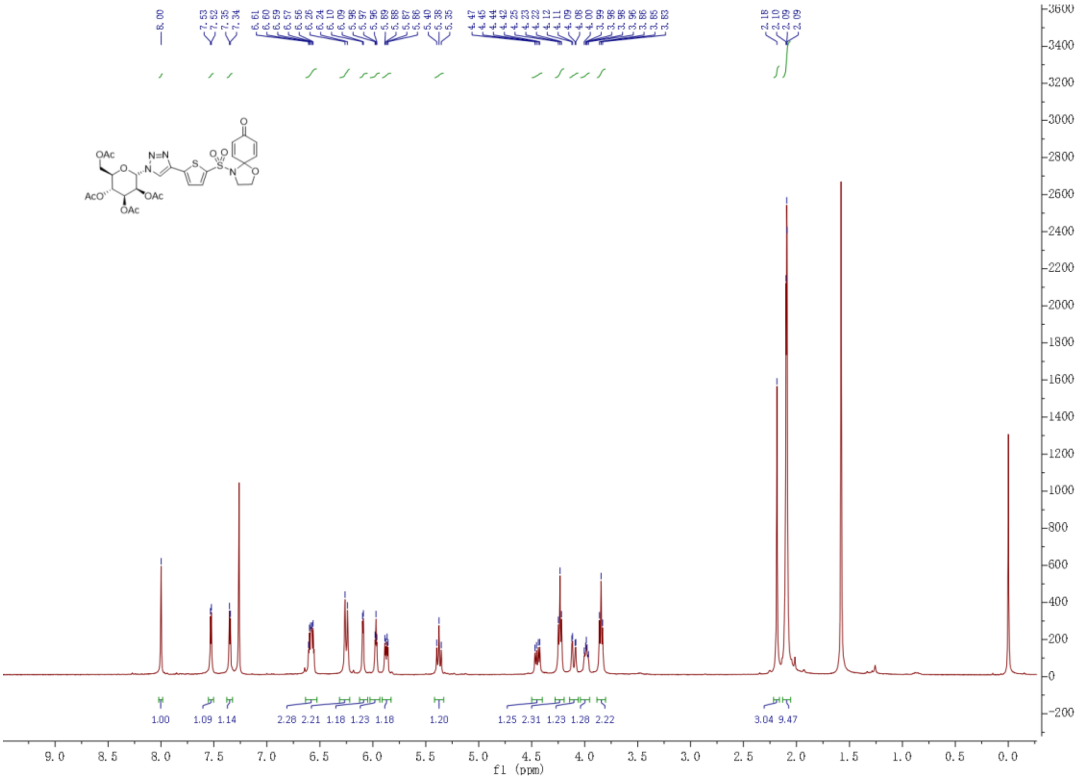

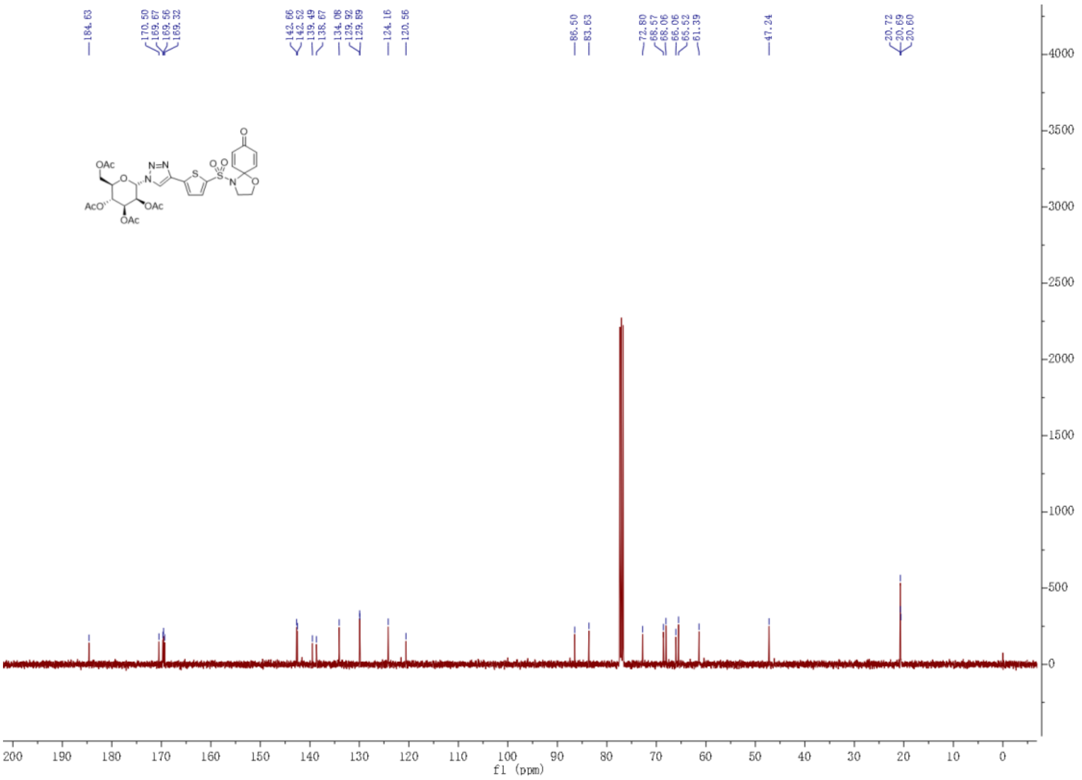

(2R,3R,4S,5S,6S)-2-(Acetoxymethyl)-6-(4-(5-((7-chloro-8-oxo-1-oxa-4-azaspiro[4.5]deca-6,9-dien-4-yl)sulfonyl)thiophen-2-yl)-1H-1,2,3-triazol-1-yl)tetrahydro-2H-pyran-3,4,5-triyl triacetate (7j)

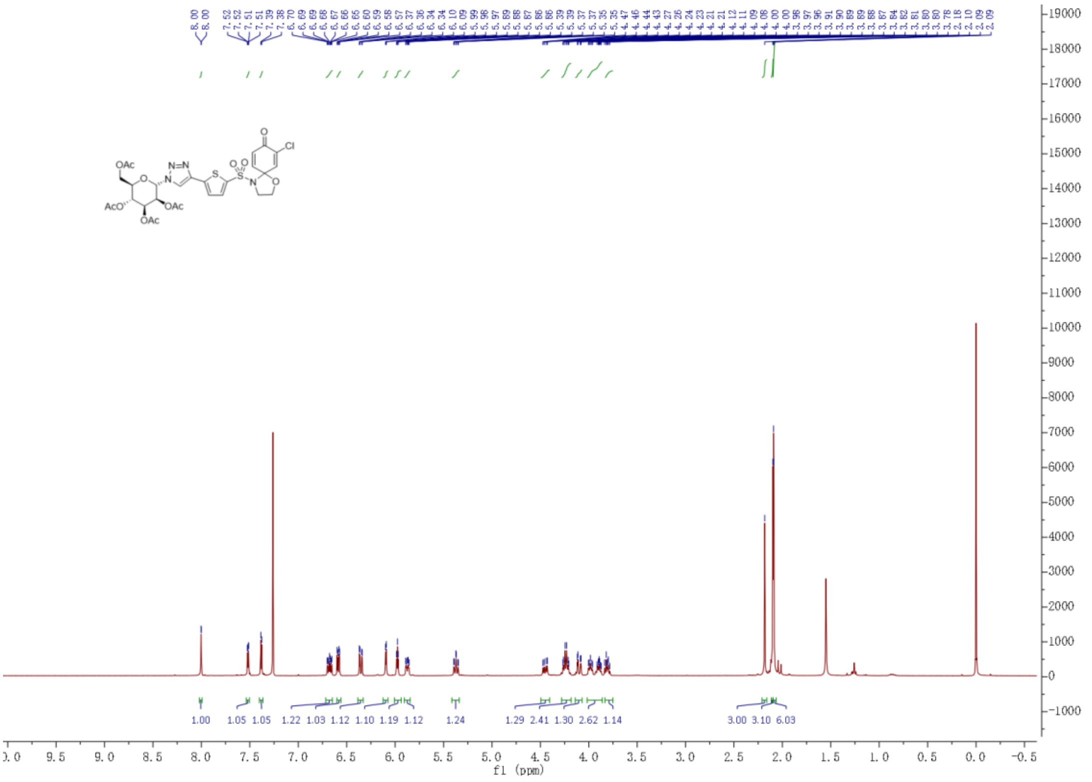

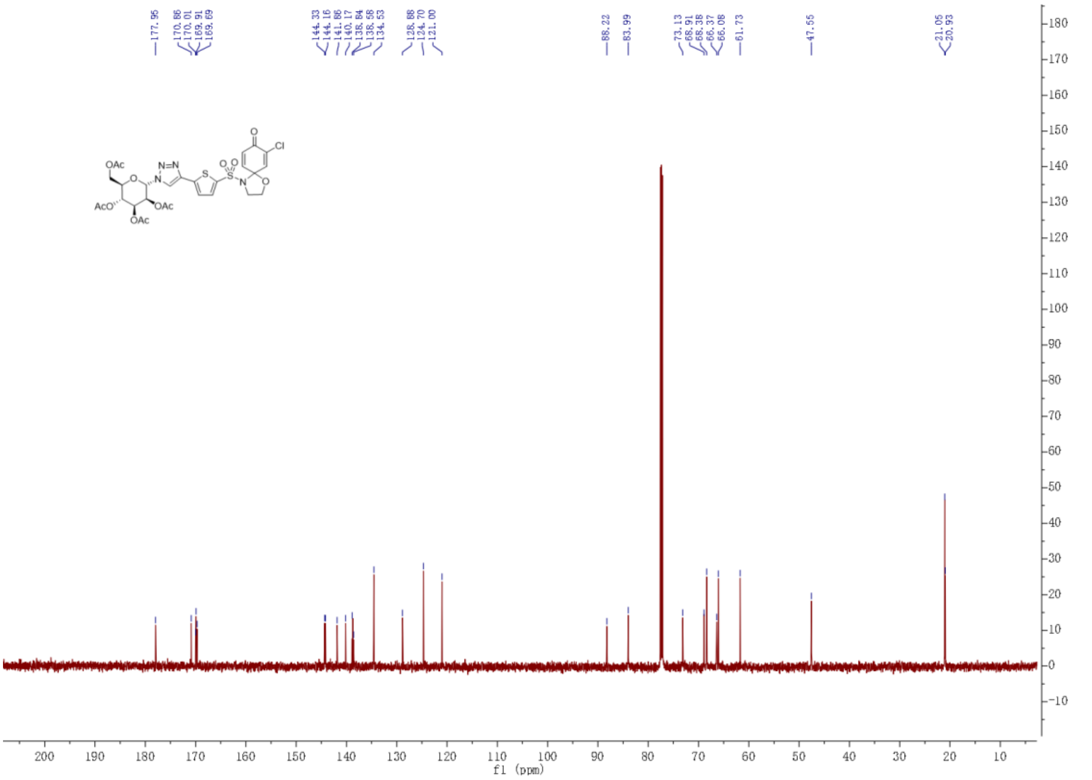

(2R,3R,4S,5S,6S)-2-(Acetoxymethyl)-6-(4-(5-((4-oxo-4H-spiro[naphthalene-1,2'-oxazolidin]-3'-yl)sulfonyl)thiophen-2-yl)-1H-1,2,3-triazol-1-yl)tetrahydro-2H-pyran-3,4,5-triyl triacetate (7k)

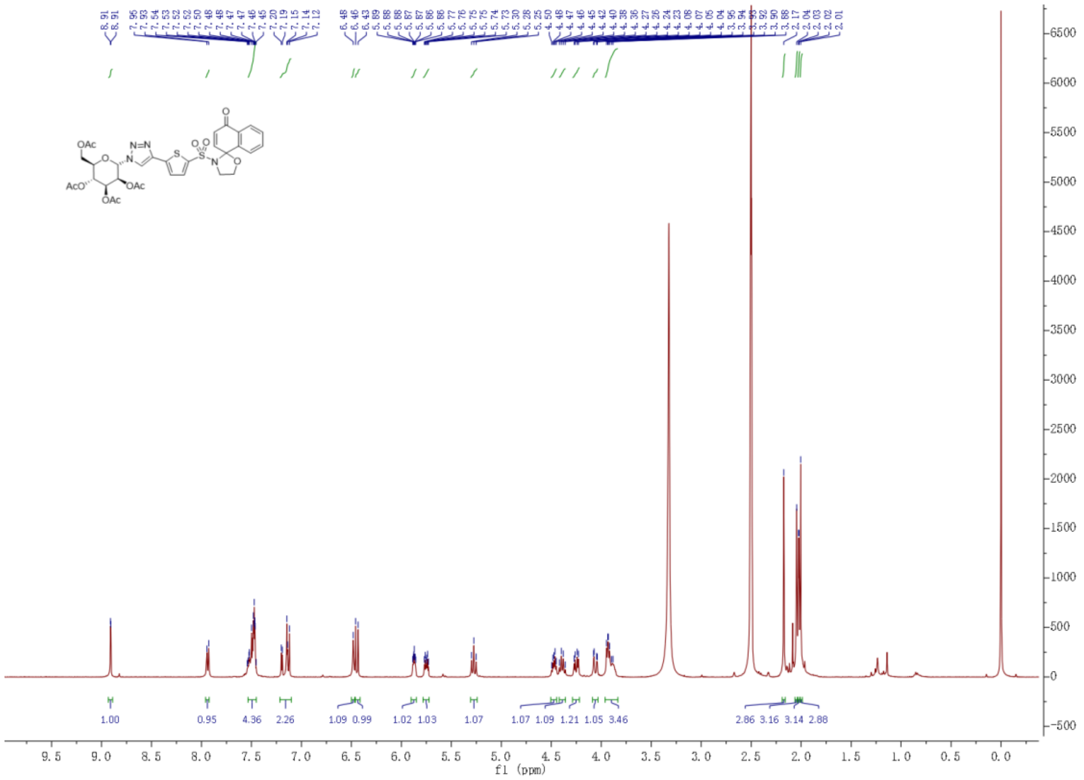

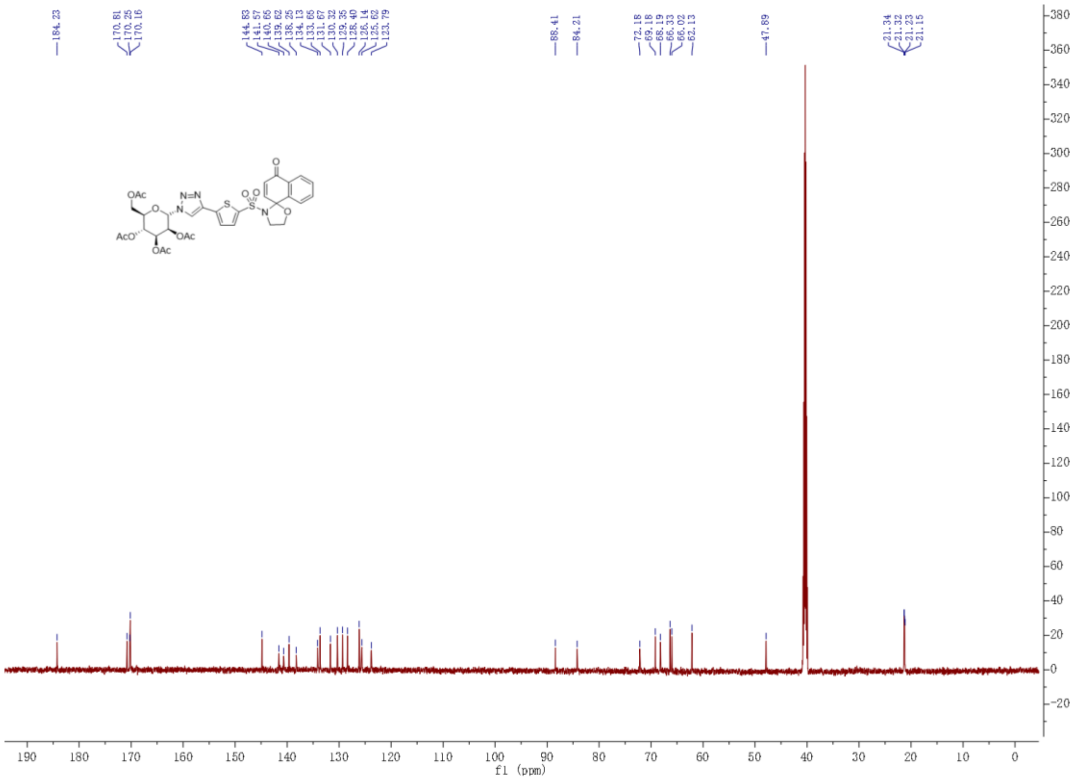

(2R,3R,4S,5S,6S)-2-(Acetoxymethyl)-6-(4-(5-((8-oxo-1-oxa-4-azaspiro[4.5]deca-6,9-dien-4-yl)sulfonyl)pyridin-2-yl)-1H-1,2,3-triazol-1-yl)tetrahydro-2H-pyran-3,4,5-triyl triacetate (7l)

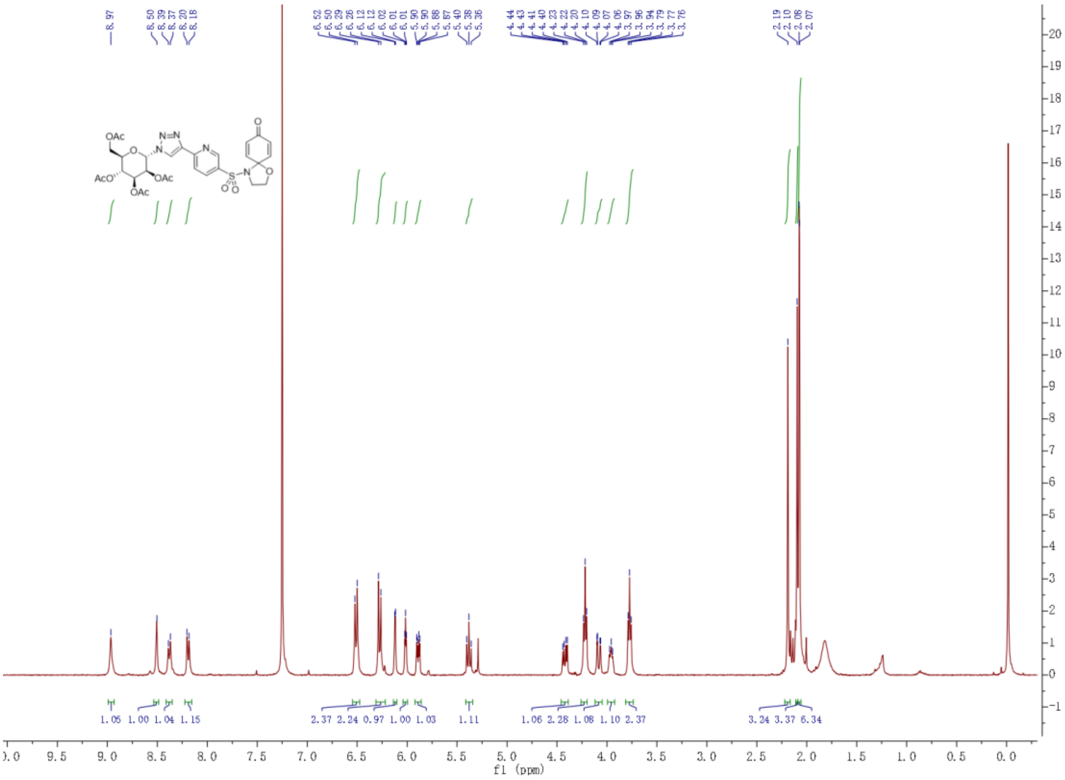

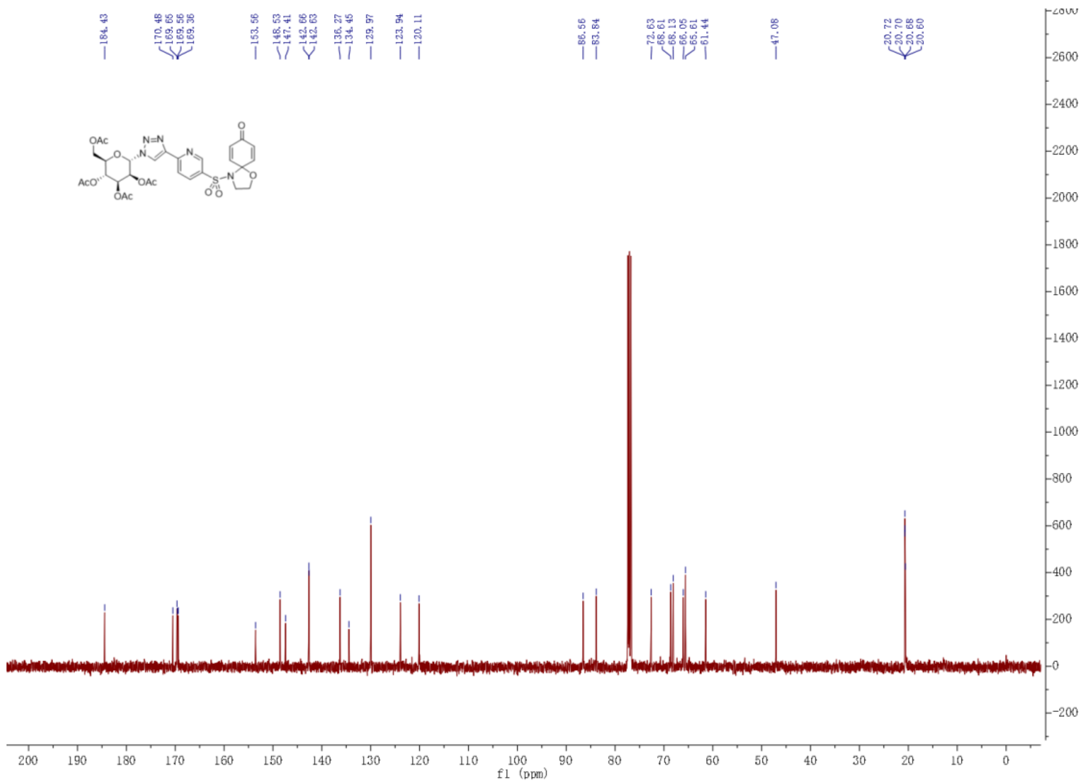

(2R,3R,4S,5R,6R)-2-(Acetoxymethyl)-6-(4-(5-((8-oxo-1-oxa-4-azaspiro[4.5]deca-6,9-dien-4-yl)sulfonyl)pyridin-2-yl)-1H-1,2,3-triazol-1-yl)tetrahydro-2H-pyran-3,4,5-triyl triacetate (7m)

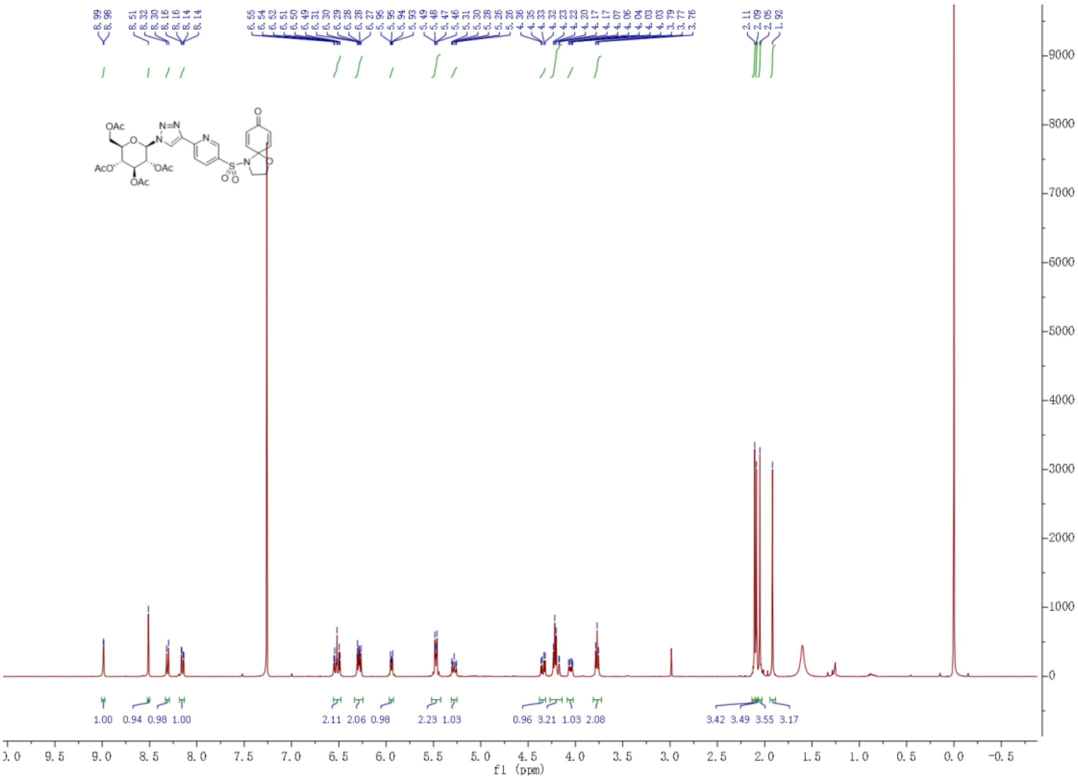

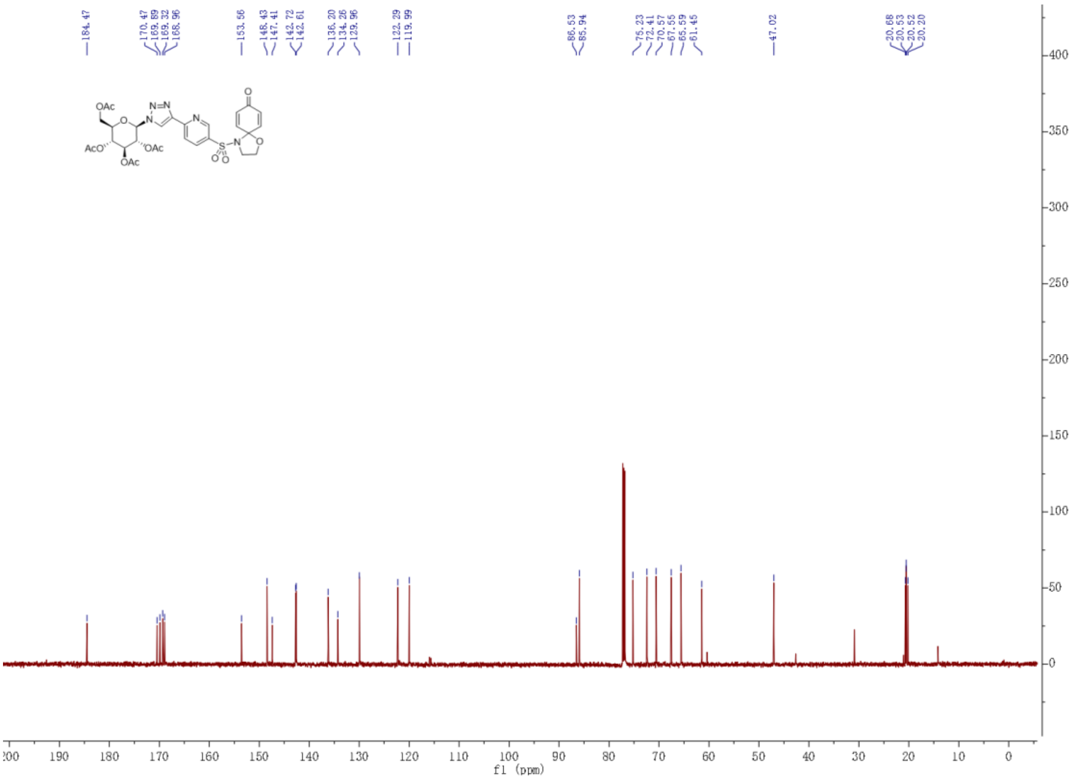

4-((5-(1-((2R,3R,4S,5S,6R)-3,4,5-Trihydroxy-6-(hydroxymethyl)tetrahydro-2*H*-pyran-2-yl)-1*H*-1,2,3-triazol-4-yl)thiophen-2-yl)sulfonyl)-1-oxa-4-azaspiro[4.5]deca-6,9-dien-8-one (8a)

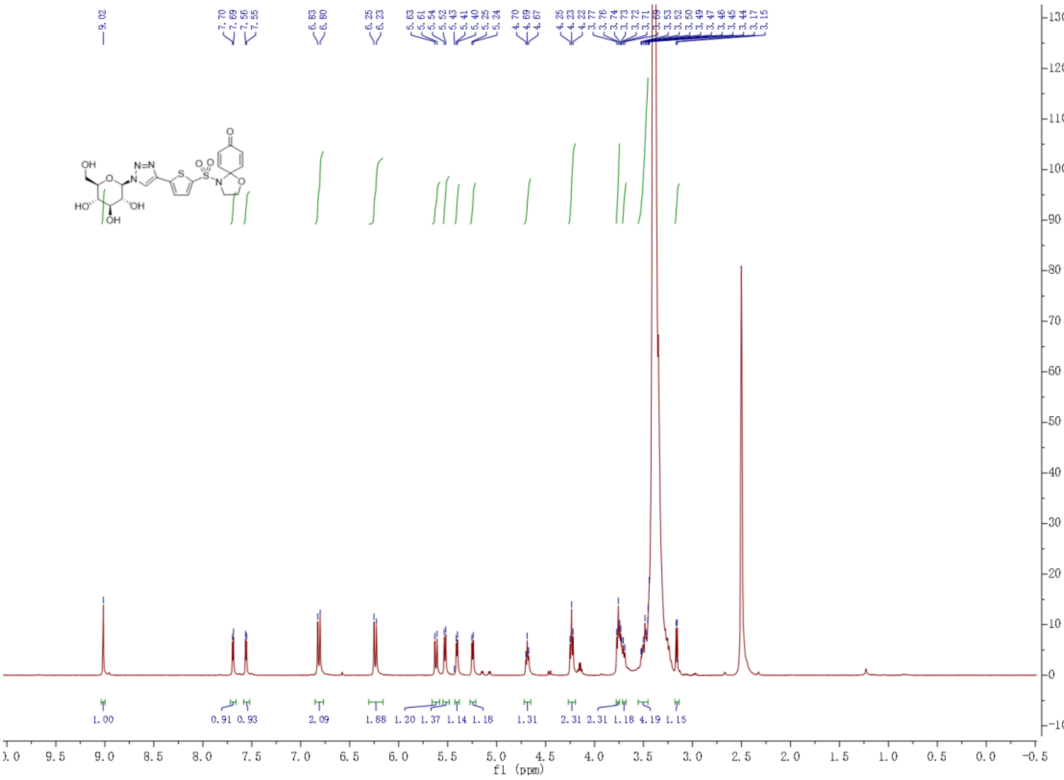

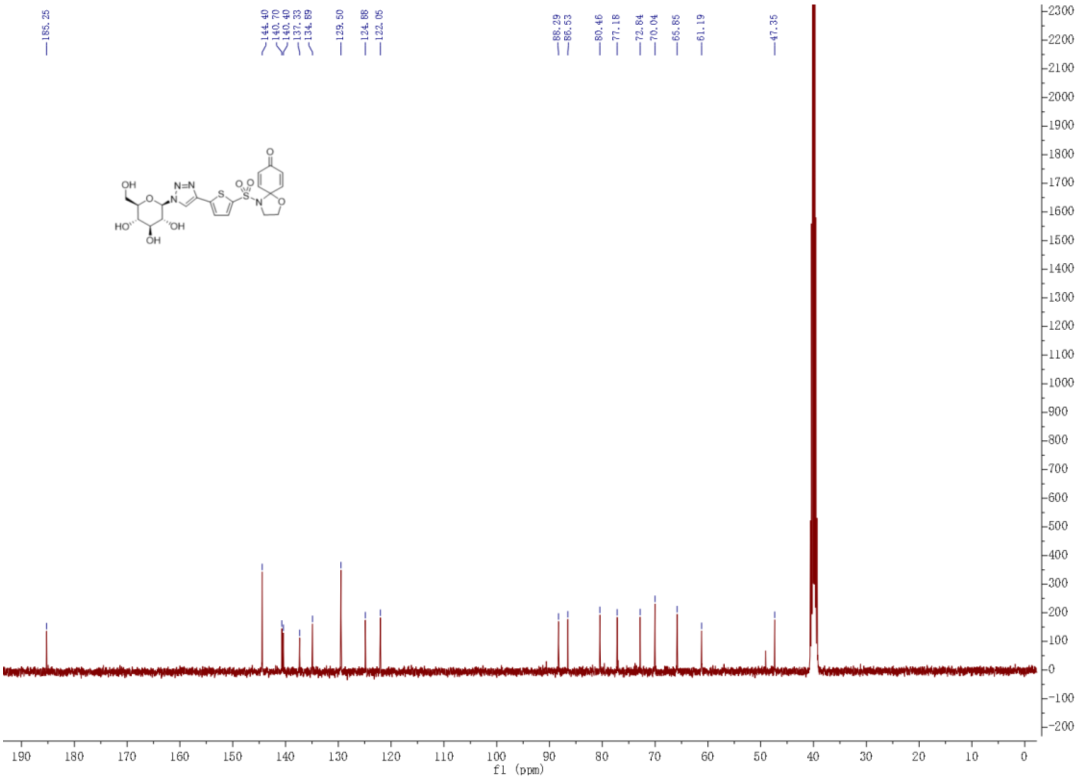

7-Chloro-4-((5-(1-((2R,3R,4S,5S,6R)-3,4,5-trihydroxy-6-(hydroxymethyl)tetrahydro-2*H*-pyran-2-

yl)-1*H*-1,2,3-triazol-4-yl)thiophen-2-yl)sulfonyl)-1-oxa-4-azaspiro[4.5]deca-6,9-dien-8-one (8b)

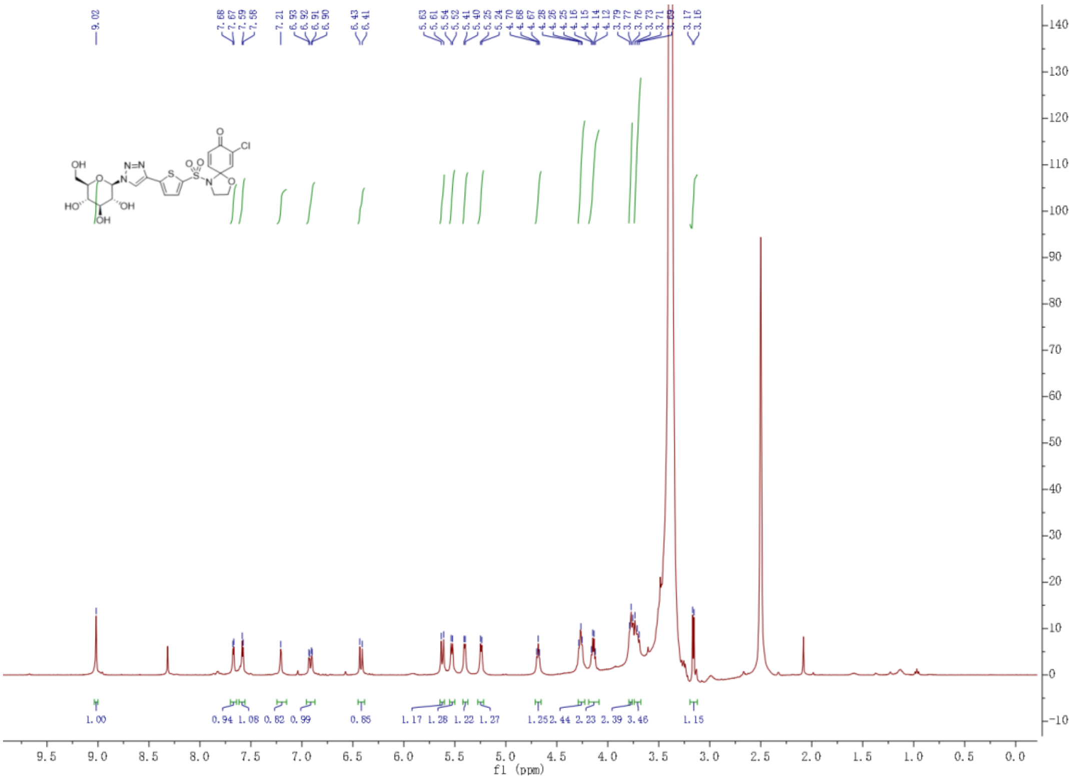

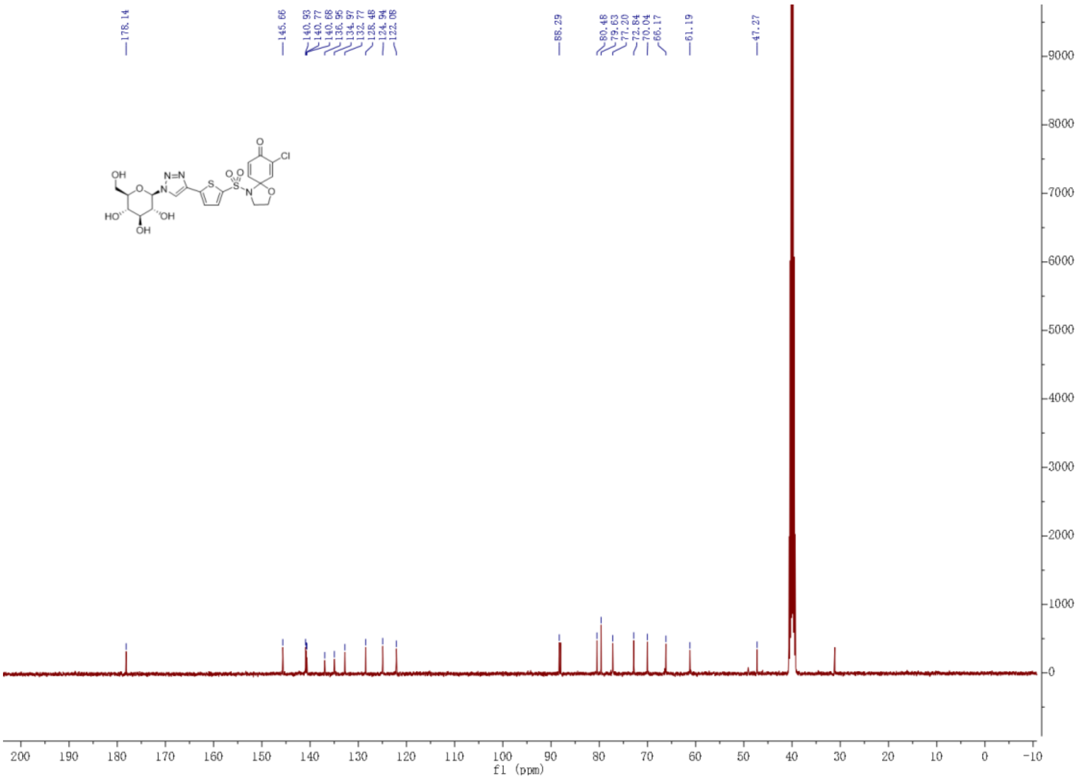

3'-((5-(1-((2R,3R,4S,5S,6R)-3,4,5-Trihydroxy-6-(hydroxymethyl)tetrahydro-2*H*-pyran-2-yl)-1*H*-1,2,3-triazol-4-yl)thiophen-2-yl)sulfonyl)-4*H*-spiro[naphthalene-1,2'-oxazolidin]-4-one (8c)

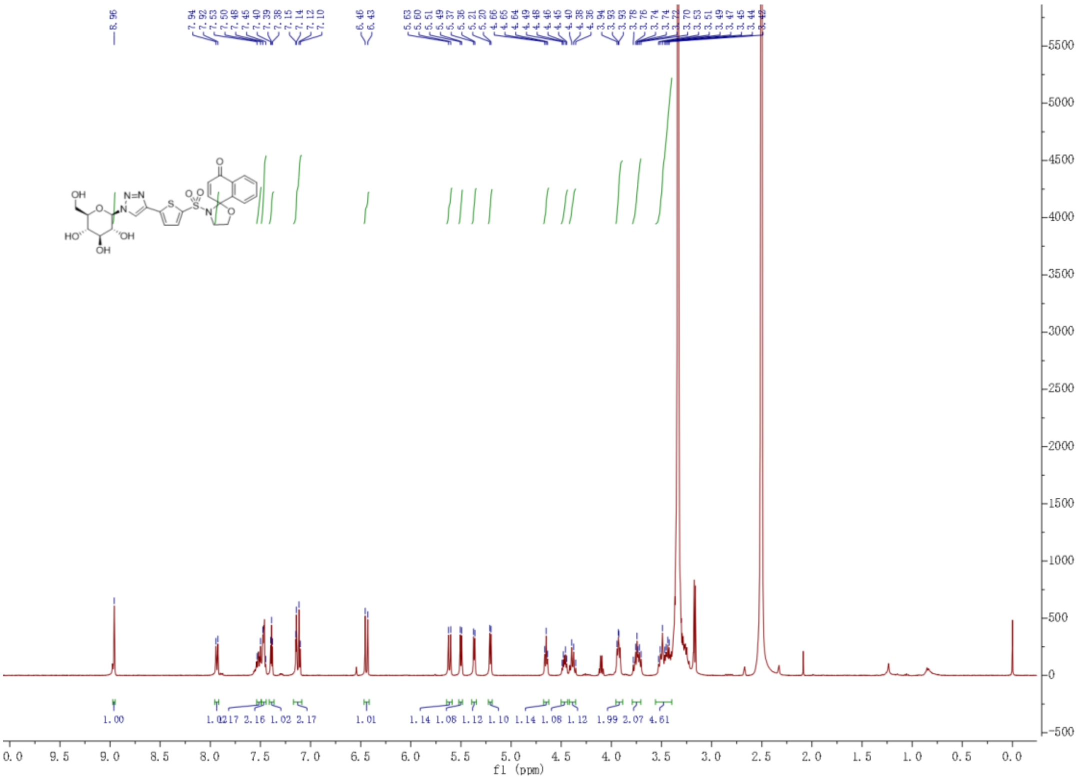

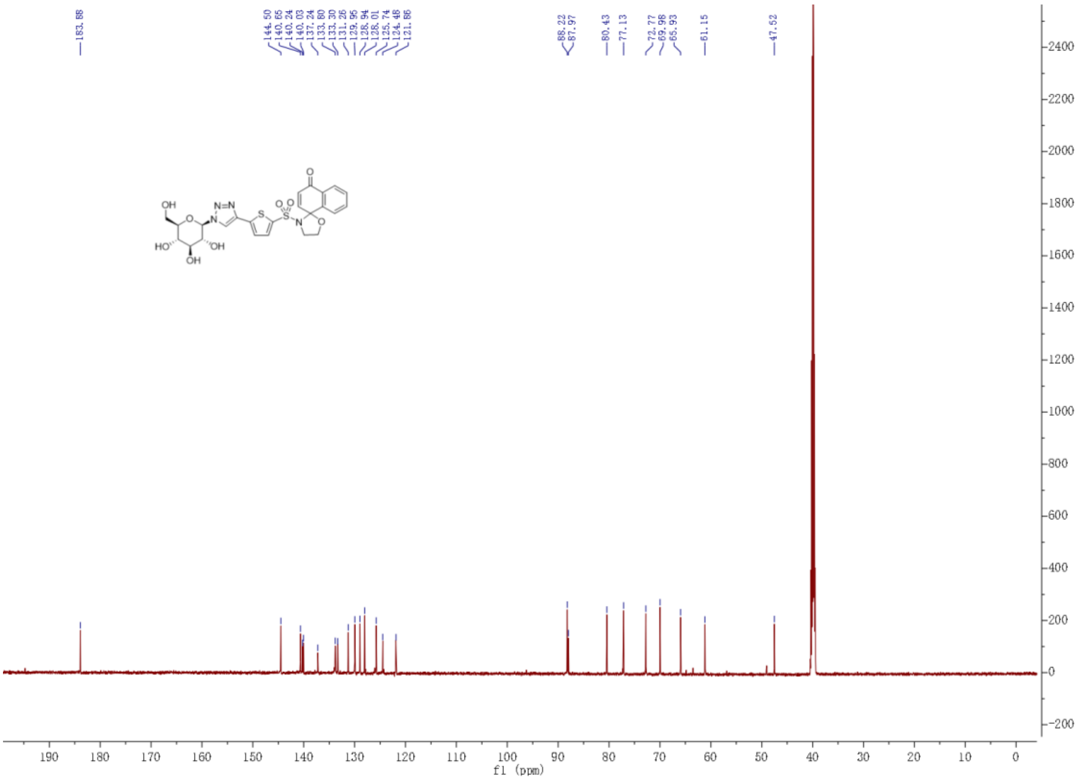

4-((5-(1-((2R,3S,4S,5S,6R)-3,4,5-Trihydroxy-6-(hydroxymethyl)tetrahydro-2*H*-pyran-2-yl)-1*H*-1,2,3-triazol-4-yl)thiophen-2-yl)sulfonyl)-1-oxa-4-azaspiro[4.5]deca-6,9-dien-8-one (8d)

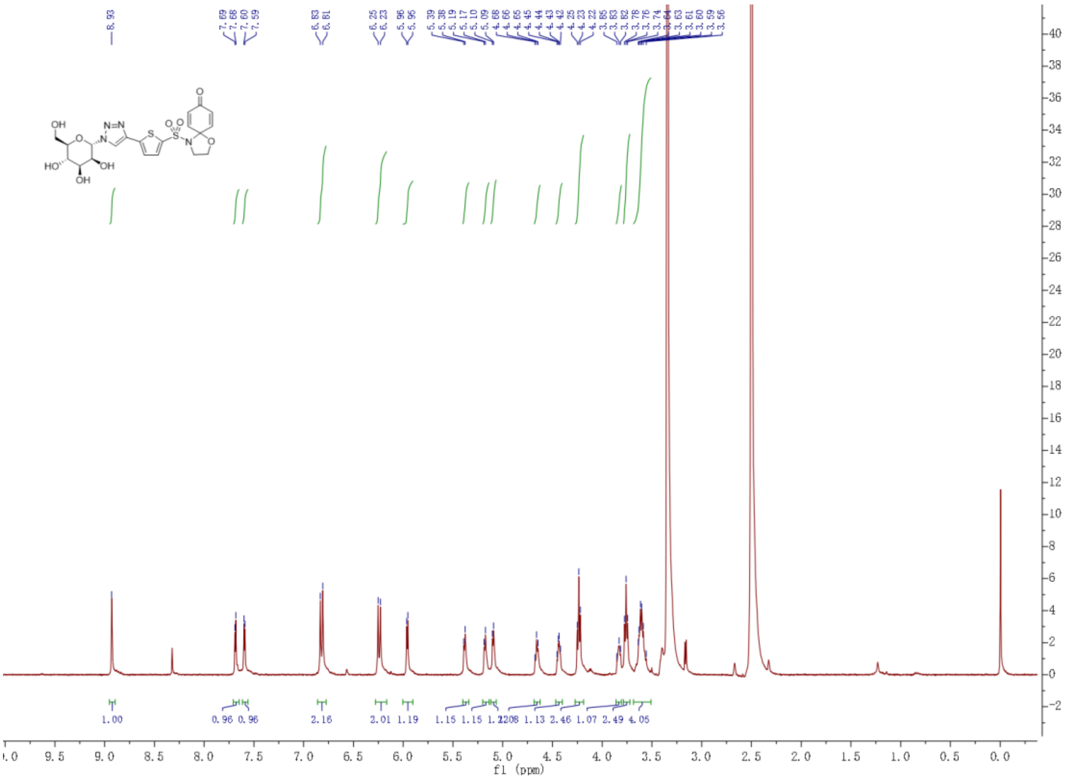

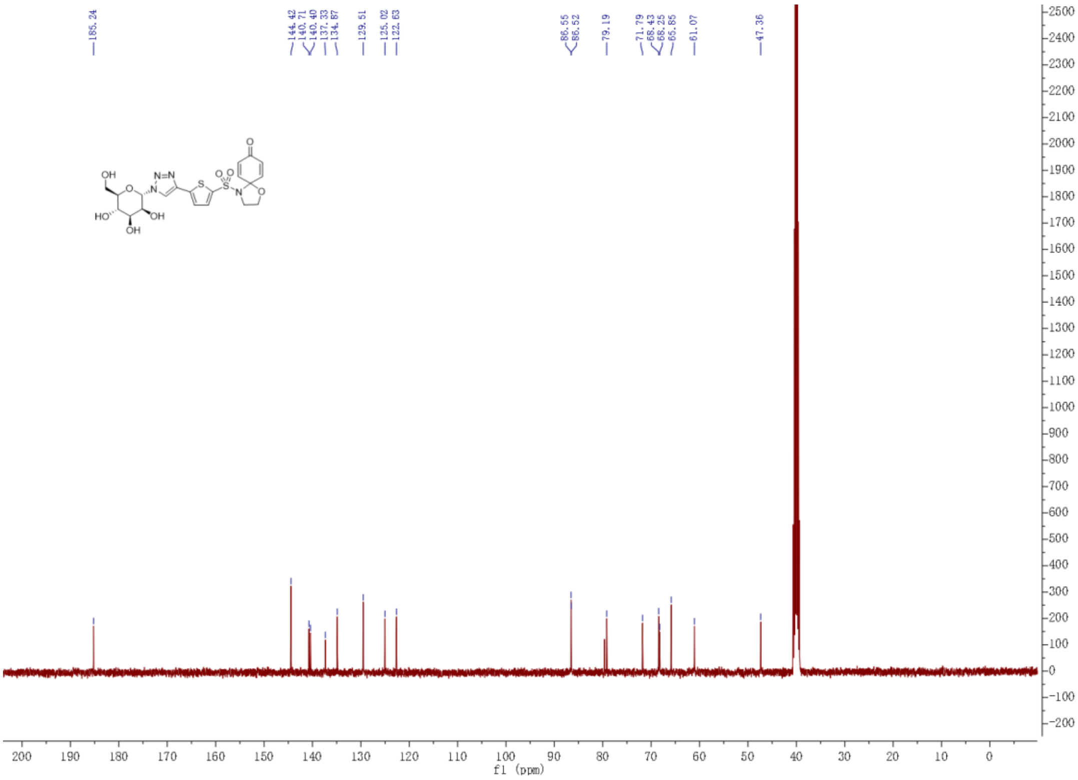

7-Chloro-4-((5-(1-((2R,3S,4S,5S,6R)-3,4,5-trihydroxy-6-(hydroxymethyl)tetrahydro-2*H*-pyran-2-yl)-1*H*-1,2,3-triazol-4-yl)thiophen-2-yl)sulfonyl)-1-oxa-4-azaspiro[4.5]deca-6,9-dien-8-one (8e)

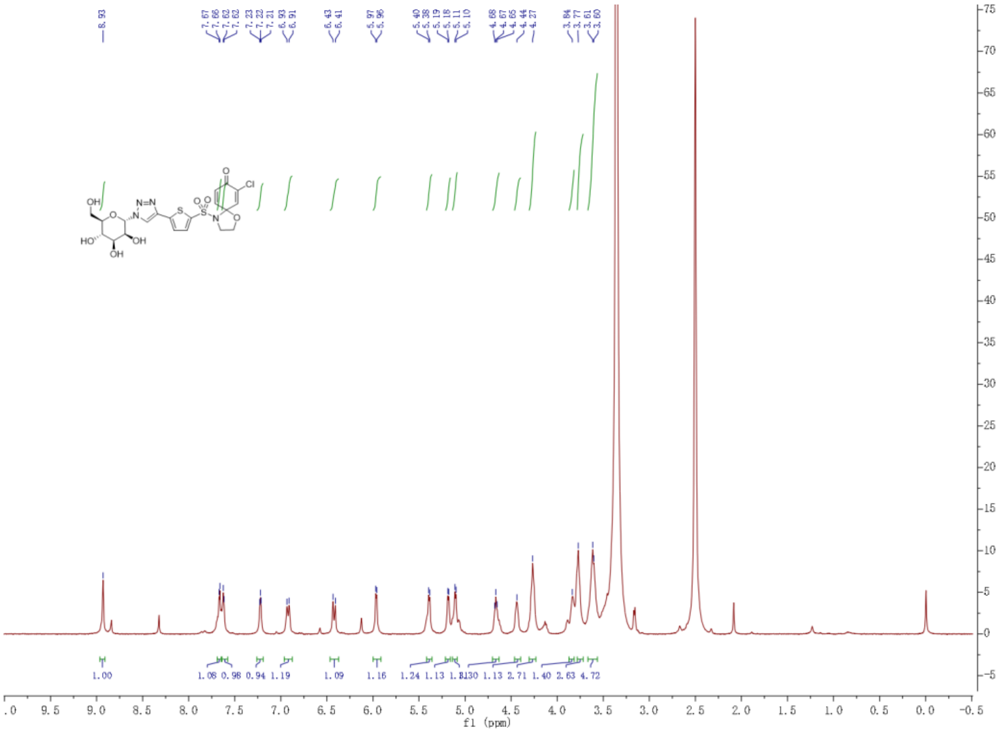

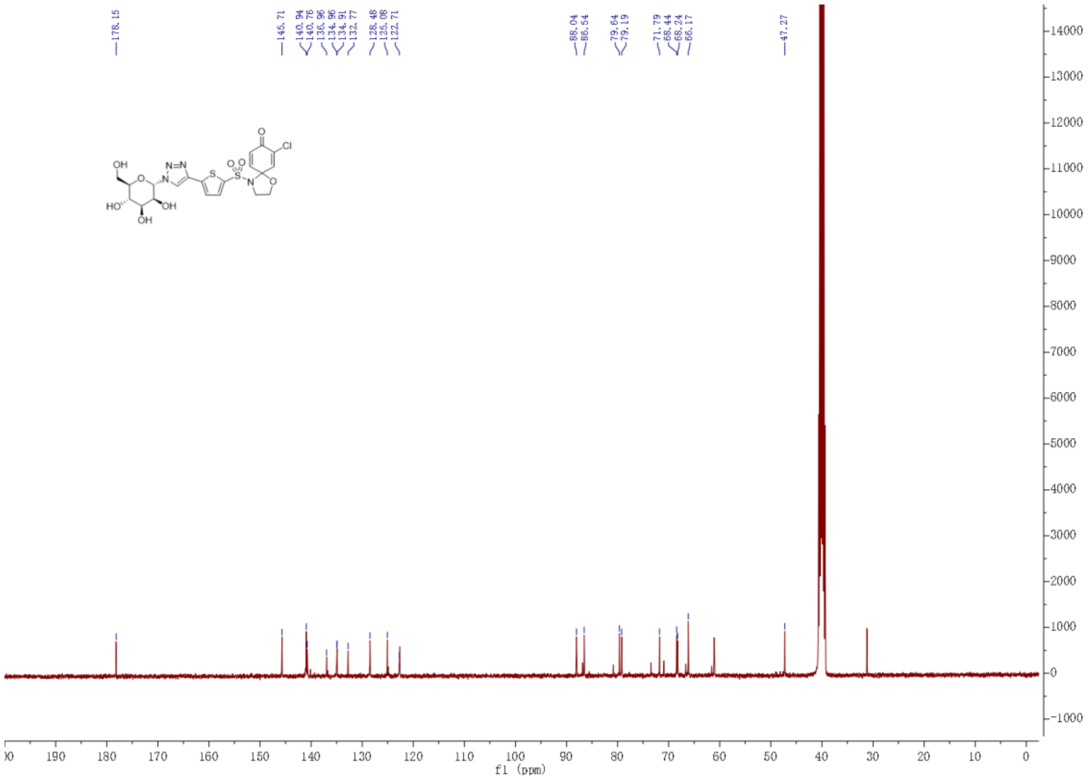

3'-((5-(1-((2R,3S,4S,5S,6R)-3,4,5-Trihydroxy-6-(hydroxymethyl)tetrahydro-2H-pyran-2-yl)-1*H*-1,2,3-triazol-4-yl)thiophen-2-yl)sulfonyl)-4*H*-spiro[naphthalene-1,2'-oxazolidin]-4-one (8f)

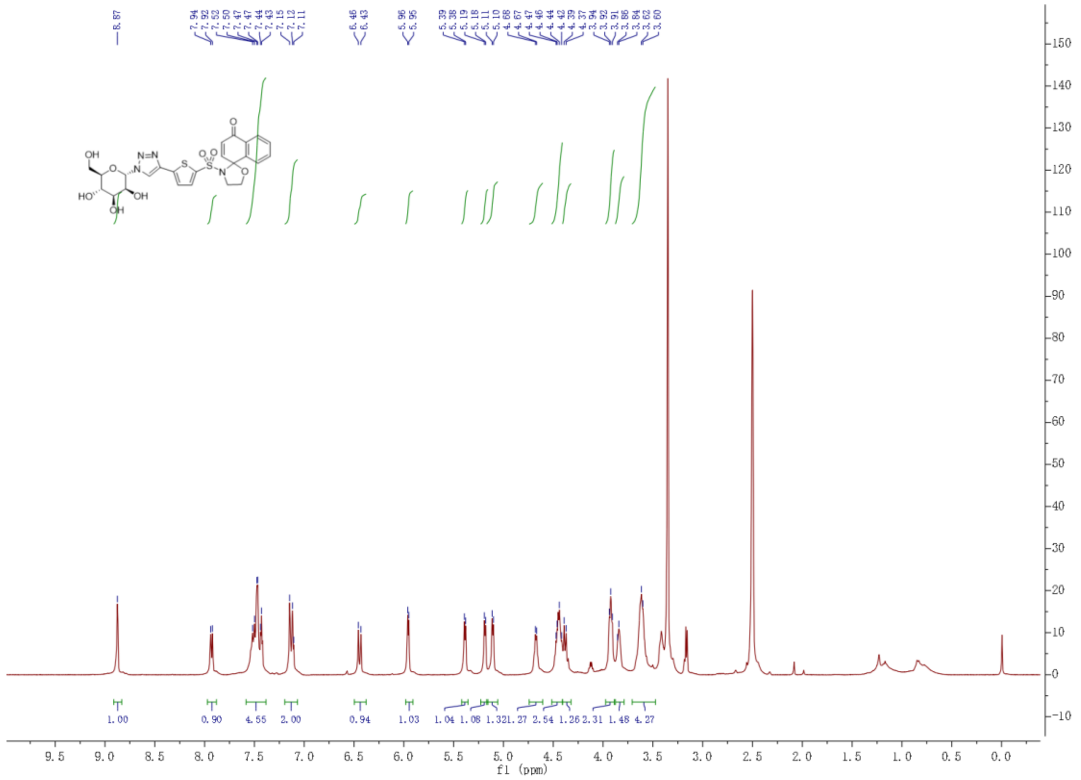

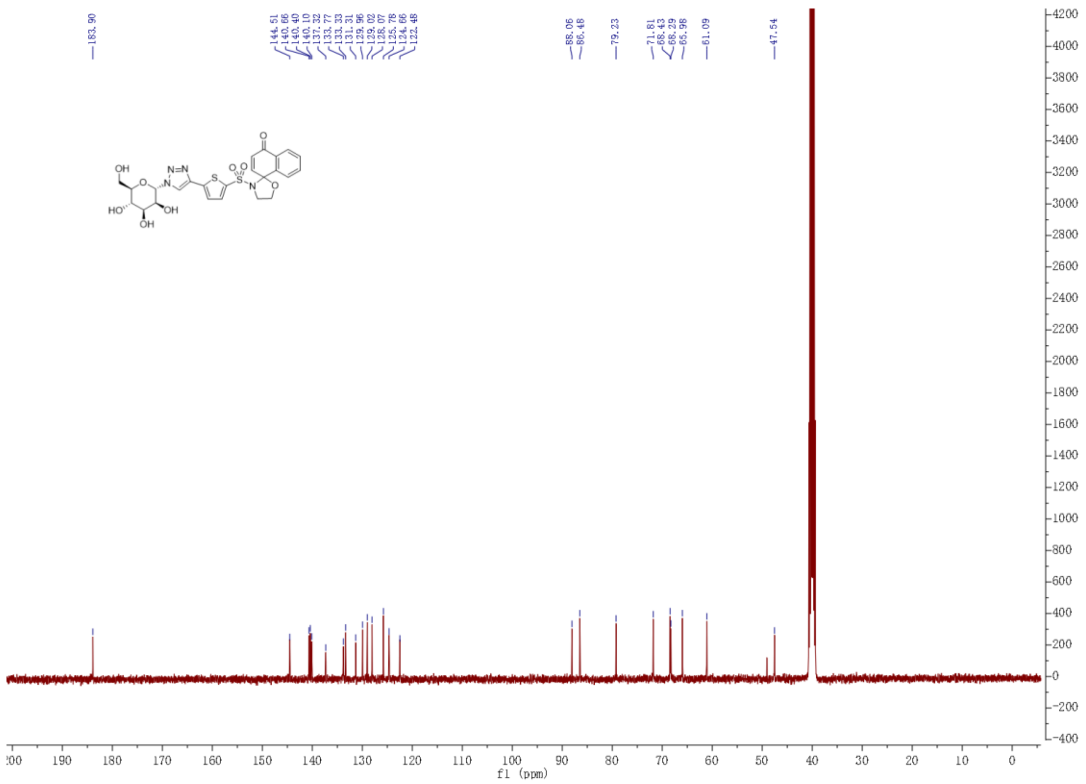

4-((6-(1-((2R,3R,4S,5S,6R)-3,4,5-Trihydroxy-6-(hydroxymethyl)tetrahydro-2*H*-pyran-2-yl)-1*H*-1,2,3-triazol-4-yl)pyridin-3-yl)sulfonyl)-1-oxa-4-azaspiro[4.5]deca-6,9-dien-8-one (8g)

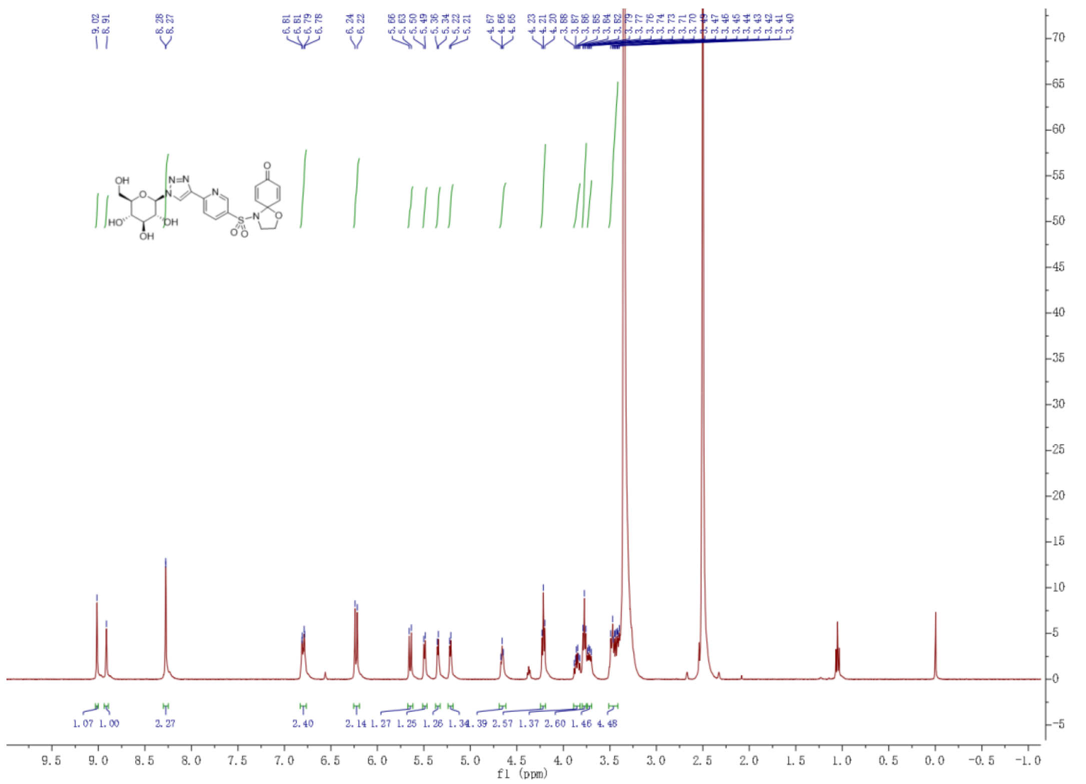

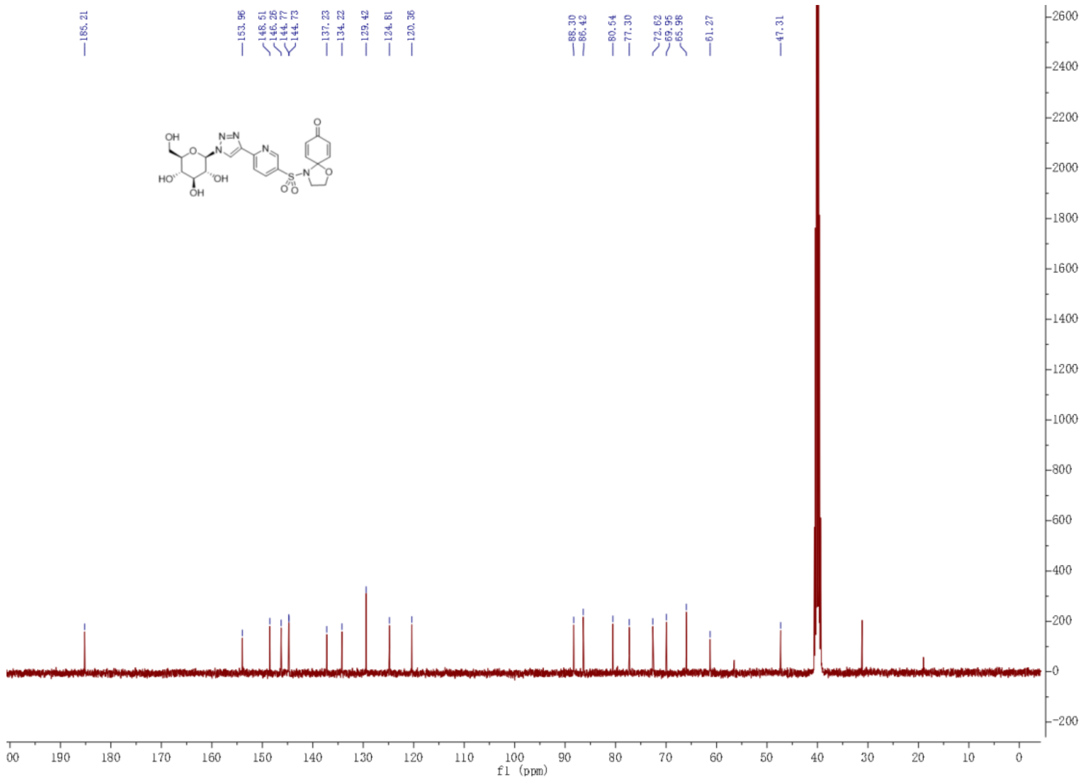

4-((6-(1-((2R,3S,4S,5S,6R)-3,4,5-Trihydroxy-6-(hydroxymethyl)tetrahydro-2*H*-pyran-2-yl)-1*H*-1,2

,3-triazol-4-yl)pyridin-3-yl)sulfonyl)-1-oxa-4-azaspiro[4.5]deca-6,9-dien-8-one (8h)

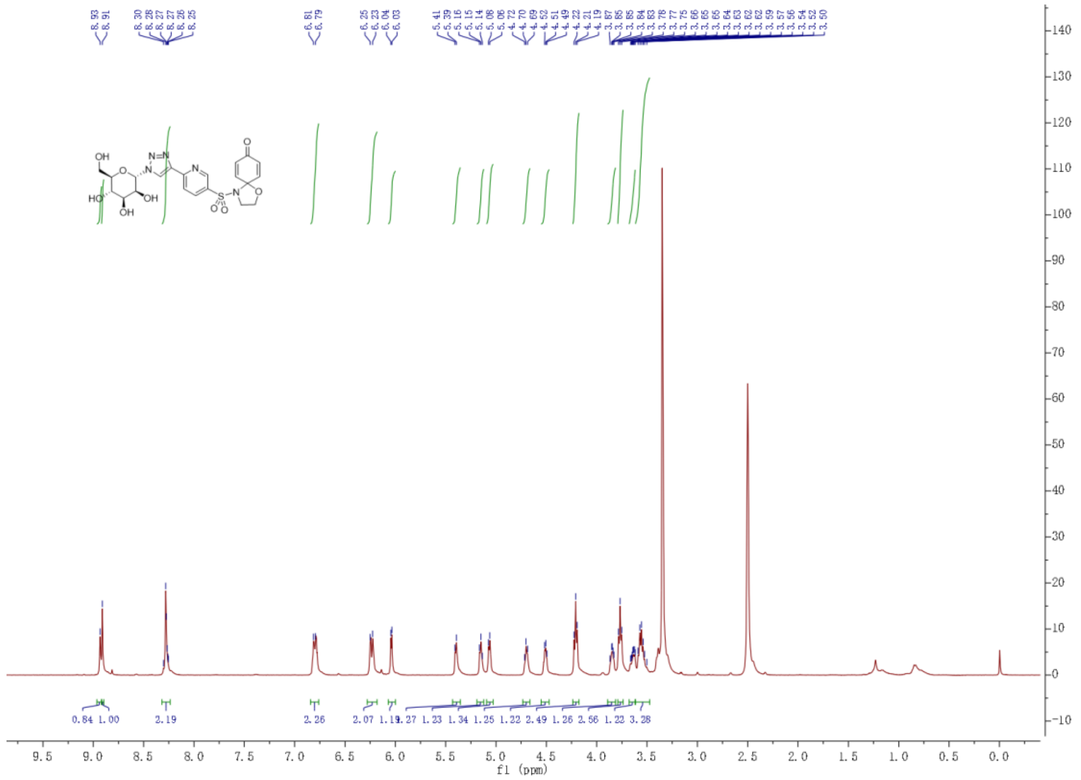

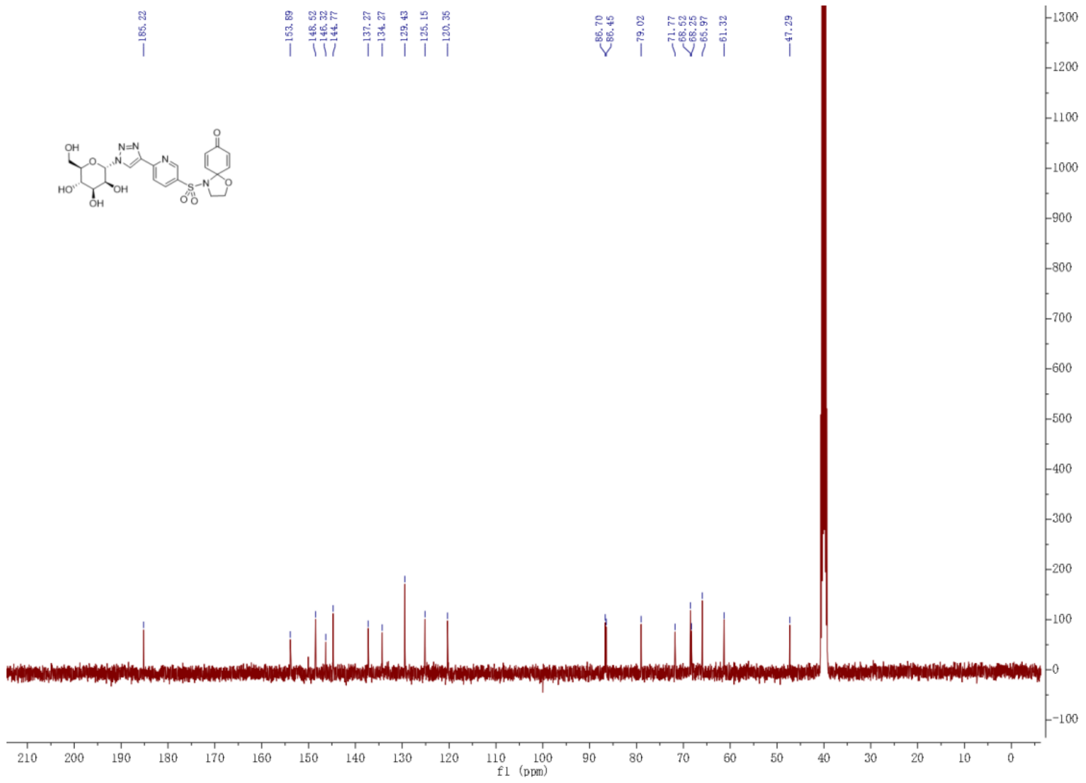

Supplement: Supplementary file 1 [file molecules-25-05459-s001.pdf]
